# Causal Effects of Exposure to Air Pollution on the Risk of Neurosurgical Multi-system Diseases: A Worldwide Study of Mendelian Randomization

**DOI:** 10.7150/ijms.115853

**Published:** 2025-07-28

**Authors:** Lirui Dai, Shu Jiang, Peizhi Zhou

**Affiliations:** Department of Neurosurgery, West China Hospital of Sichuan University, Sichuan University, Chengdu, Sichuan, China.

**Keywords:** air pollution, neurosurgical multi-system diseases, Mendelian randomization, genetic associations

## Abstract

**Background:** Epidemiological studies has investigated the correlation between ambient air pollution and neurosurgical multisystem diseases. Multiple studies have shown that air pollution significantly influences various neurological disorders. Nevertheless, the findings from these studies are inconsistent and contentious, leaving the causal relationships for many conditions unresolved. The study systematically investigates the underlying genetic causal relationships between air pollution and neurosurgical multisystem diseases, as well as to assess the implications of these associations.

**Methods:** Genetic instruments for particulate matter (PM) with aerodynamic diameter < 2.5 μm (PM_2.5_), < 2.5-10 μm (PM_2.5-10_), <10 μm (PM_10_), PM_2.5_ absorbance, nitrogen dioxide (NO_2_), nitrogen oxides (NOx) and 30 neurosurgical multi-system diseases were selected.

**Results:** In the European population, a noteworthy causal association was identified between NO_2_ and PM_2.5_ exposure and cerebral infarction (IVW: OR = 1.03, 95%CI: 1.01~1.06). Among African American or Afro-Caribbean individuals, NOx (IVW: OR = 0.63, 95%CI: 0.44~0.90) and NO_2_ (IVW: OR = 0.68, 95%CI: 0.54-0.87) are predisposed to trigger trigeminal neuralgia, while PM_2.5_ is related to 3 neurosurgical diseases, including epilepsy (IVW: OR = 0.89, 95%CI: 0.79~1.00), subarachnoid hemorrhage (IVW: OR = 0.75, 95%CI: 0.61~0.91), and diffuse brain injury (IVW: OR = 0.67, 95%CI: 0.47~0.96). In East Asian populations, a correlation has been observed between PM_2.5_ (IVW: OR = 0.99, 95%CI: 0.98~1.00) and PM_10_ (IVW: OR = 1.00, 95%CI: 1.00~1.00) exposure and the occurrence of cervical spondylosis. Additionally, there is a genetic susceptibility to pituitary adenoma and craniopharyngioma related to NO_2_ (IVW: OR = 1.24, 95%CI: 1.02~1.52) and PM_2.5_ absorbance (IVW: OR = 0.73, 95%CI: 0.59~0.90). In South Asian populations, there is a significant genetic susceptibility to the influences of PM_2.5-10_ (IVW: OR = 0.90, 95%CI: 0.83~0.97) on stroke incidence. In contrast, for populations in the Greater Middle East, air pollution is predominantly associated with cerebrovascular diseases. For example, PM_2.5-10_ shows a positive genetic predisposition towards stroke (IVW: OR = 1.02, 95%CI: 1.00~1.05) and subarachnoid hemorrhage (IVW: OR = 1.06, 95%CI: 1.00~1.12).

**Conclusion:** This study presents the first genetic evidence establishing a connection between air pollution and neurosurgical multisystem diseases. Our findings emphasize the importance of air quality in the context of these diseases, potentially offering new insights into the underlying mechanisms and informing future clinical research on air pollution-mediated neurosurgical conditions, particularly cerebrovascular and brain functional disorders.

## 1. Introduction

**Figure 1:**
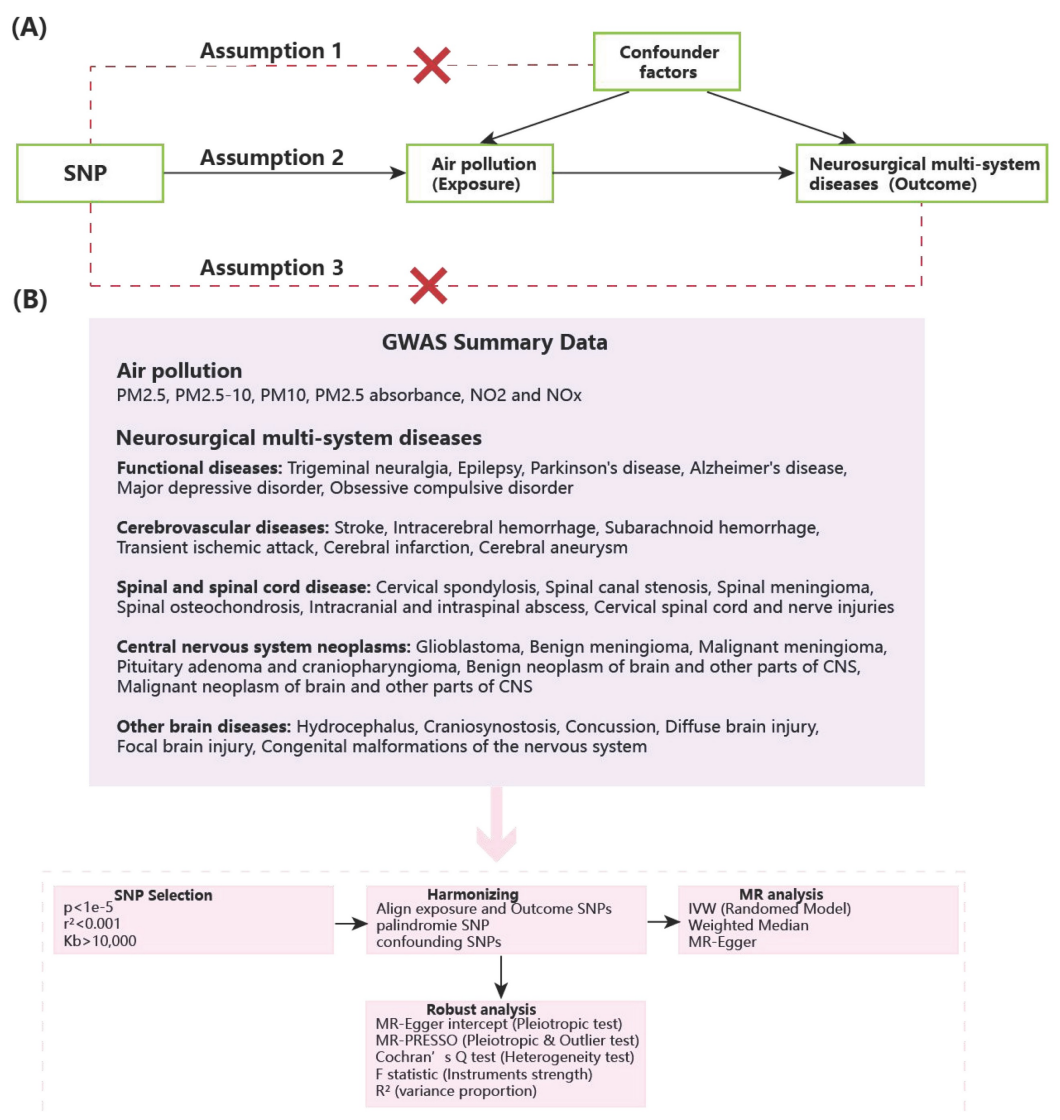
The flow chart of the MR analysis and study design. A. MR assumption. 1. SNPs are not associated with confounder; 2. SNPs are strongly associated with air pollution; 3. SNPs influence neurosurgical multi-system diseases solely through air pollution. B. Analysis flow chart.

Emerging evidence suggests that ambient air pollution, comprising complex mixtures of various PM and NOx 1, poses multiple adverse hazards to human health and may elevate the morbidity and mortality rates of cardiovascular, cerebrovascular, and oncological diseases 2, 3. Studies have showed that air pollution can enhance the risk of mortality from non-communicable diseases by nearly 20% 2. Notably, air pollution and the occurrence of neurosurgical multisystem diseases has underlying correlation. The neurosurgical multisystem diseases here are classified into five major categories based on clinical experience, including functional diseases, cerebrovascular diseases, Spinal and spinal cord diseases, central nervous system neoplasms and Other brain diseases, which facilitates our summary of the relationship between different types of neurosurgical diseases and environmental pollution (Details in Figure 1). Since the 1990s, epidemiological data from multiple research teams across North America, Asia, and Europe have demonstrated a correlation between air pollution and cerebrovascular disease mortality. Regions with elevated gaseous pollutants, including PM2.5 and ozone, have experienced a significant increase in cerebrovascular accidents 4. Furthermore, numerous studies have also explored the influence of air pollution on other neurological disorders. For example, Shreya Louis et al. found that epilepsy is closely associated with air pollution and temperature, while long-term and short-term exposure to air pollution, such as PM_2.5_, PM_10_, PM_2.5-10_ and NOx were significantly associated with ischemic stroke and transient ischemic attack 3. Ma et al. discovered that prolonged exposure to high concentrations of PM_2.5_ can lead to the accumulation of amyloid protein, thereby heightening the risk of cognitive impairment and influencing the neurobiological characteristics associated with Alzheimer's disease 5. Additionally, study has indicated that exposure to air pollution is relevant to increase of risk both benign 6 and malignant brain tumors 7. However, further investigation is required to elucidate the specific types of tumors involved and the underlying mechanisms of their development.

Therefore, the influence of ambient air pollution on neurosurgical multi-system diseases cannot be ignored 8-10, and these pollutants bring a heavy burden to the human body and aggravate the comorbidities 11. Nowadays, many prospective studies and epidemiological investigations have displayed that air pollution is highly relevant to a variety of neurological diseases, such as stroke 12, Alzheimer's disease 13, Parkinson's disease 14 and epilepsy 15. Due to the regional or population-specific focus of these studies, rather than utilizing a diverse array of clinical samples across various races and regions, the findings are subject to confounding factors and lack systematic generalizability. The aim of this study is to systematically survey the correlation between neurosurgical multi-system diseases and air pollution across different races and populations using Mendelian randomization analysis. Furthermore, it seeks to comprehensively summarize the impact of air pollution on neurological diseases, and provide research basis for guiding neurosurgeons to treat related diseases and local governments to control air pollution.

Our study indicates that genetic polymorphisms can enhance our comprehension of environmental health risks. To address the aforementioned methodological challenges, we utilized Mendelian randomization (MR) analysis 16. The MR approach, grounded in Mendel's second law of inheritance to mitigate confounding factors 17, utilizes genetic variation as instrumental variables (IVs) to evaluate the correlation between air pollution and neurosurgical multisystem diseases. We proceeded the two-sample MR analysis applying data from genome-wide association studies (GWAS) [18]and UK Biobank 19 data in this research. It is noteworthy that, in recent years, numerous researchers have employed MR techniques to probe underlying relationships between ambient air pollution and various tumors 7, adverse pregnancy 20, cardiovascular disease 21, autoimmune disease 22, chronic obstructive pulmonary disease 23, cognitive ability 24, and amyotrophic lateral sclerosis 25. However, to date, no researchers have applied MR methods to study the effects of air pollution on neurological diseases.

Therefore, this study aims to utilize comprehensive data on ambient air pollution and neurosurgical multisystem diseases to elucidate their causal relationship, thereby informing strategies to mitigate the influence of air pollution on neurological conditions through public health prevention and intervention measures.

## 2. Methods

### 2.1. Overall research design

Figure 1 elucidates the whole study design. We adopted MR analysis to survey the causal correlation between ambient air pollution and neurosurgical multisystem diseases. The study followed strictly to the STROBE-MR guidelines 26 and was meticulously designed based on three key hypotheses, including: 1. The IVs in this study are genetic variations closely related to ambient air pollution, specifically SNPs. 2. These genetic variations are not relevant to confounding variables and influence disease outcomes solely through the specific exposure under investigation. 3. The genetic variants impact neurological diseases exclusively via ambient air pollution, without involvement of alternative pathways 27. Previous studies have confirmed that particulate matter and nitrogen oxides can directly damage the central nervous system by disrupting the blood-brain barrier, causing neuroinflammation and oxidative stress 28. Therefore, this study mainly selected PM2.5, PM2.5-10, PM10, PM2.5 absorbance, NO2 and NOx for in-depth research. For O₃ and SO₂, due to significant data gaps in O₃ monitoring and a notable decrease in SO₂ concentration in recent years, which limited the epidemiological association analysis, they were not included in this study. The diseases discussed in this study include 30 neurological diseases.

### 2.2. Summary datasets and populations

**Figure 2:**
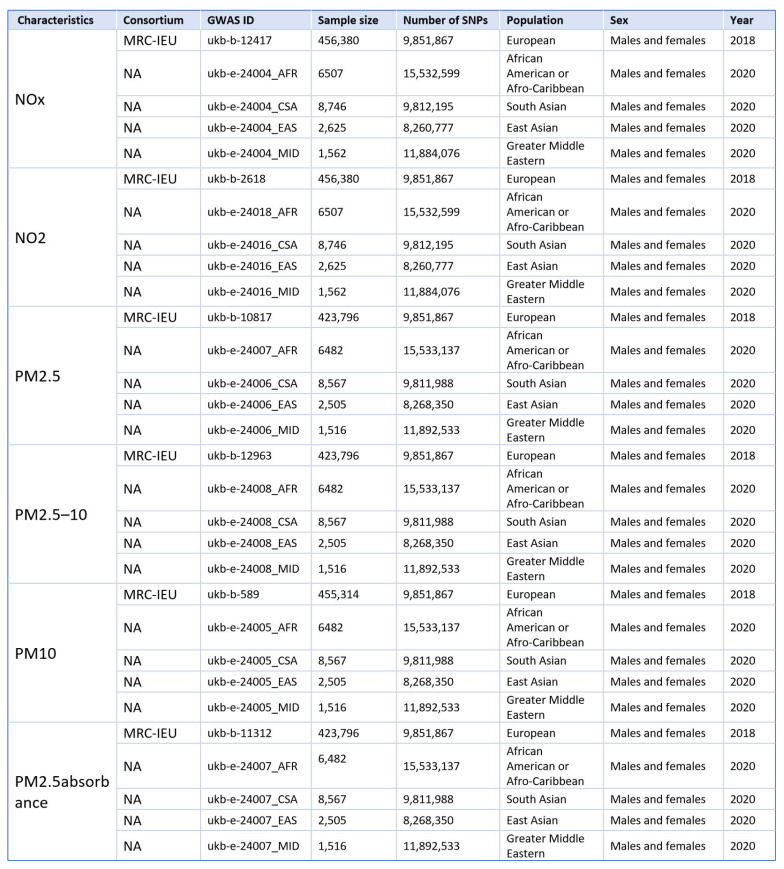
Details of GWAS in the present study (exposure).

We obtained ambient air pollution via IEU Open GWAS (https://gwas.mrcieu.ac.uk/) 29 database as exposure factors for populations of European, African American or Afro-Caribbean, South Asian, East Asian, and Greater Middle Eastern. Ambient air pollution is caused by various air pollution, such as PM_2.5_, PM_10_, PM_2.5-10_, NOx, suspended particles (TSP), carbon oxides (CO, CO_2_), hydrocarbons (CH_4_) and other harmful substances 30. Since the 1990s, PM_2.5_ is the most widely studied air pollution, followed by nitrogen oxides such as NO_2_ and NOx 31. In recent years, particles with an aerodynamic diameter of 2.5 to 10μm are also receiving increasing attention, and different pollution have different degrees of damage to human health 32. Based on this, we selected the above pollutants as the main exposure factors for MR analysis (Fig.2).

The GWAS database from Neale's lab provides the source of data on neurosurgical multisystem disorders, including Trigeminal neuralgia (195,847 cases), Epilepsy (458,310 cases), Parkinson's disease (480,018 cases), Alzheimer's disease (487,511 cases), Major depressive disorder (56,637 cases), Obsessive compulsive disorder (33,925 cases), Stroke (446,696 cases), Intracerebral hemorrhage (473,513 cases), Subarachnoid hemorrhage (473,255 cases), Transient ischemic attack (214,634 cases), Cerebral infarction (361,194 cases), Cerebral aneurysm (473,683 cases), Cervical spondylosis (484,598 cases), Spinal canal stenosis (454,787), Spinal meningioma (218,792 cases), Spinal osteochondrosis (164,865 cases), Intracranial and intraspinal abscess (217,626 cases), Cervical spinal cord and nerve injuries (215,730 cases), Glioblastoma (218,792 cases), Benign meningioma (218,792 cases), Malignant meningioma (218,792 cases), Pituitary adenoma and craniopharyngioma (218,792 cases), Benign neoplasm of brain and other parts of CNS (218,792 cases), Malignant neoplasm of brain and other parts of CNS (218,792 cases), Hydrocephalus (206,548 cases), Craniosynostosis (218,792 cases), Concussion (147,103), Diffuse brain injury (137,232 cases), Focal brain injury (137,641 cases) and Congenital malformations of the nervous system (218,792 cases) (Table 1). The above diseases in each major category are all common in clinical practice and also the most frequently encountered diseases in our daily operations in neurosurgery or in collaboration with the neurology department.

### 2.3. Selection of SNPs

To ascertain that ambient air pollution possesses a sufficient number of IVs to maintain statistical efficacy and meet the three hypothesis of MR analysis, we chose SNPs that demonstrated a strong relevance to air pollution (p<1e-5) (Table S1-S28). We employed thresholds of R²<0.001 and a distance greater than 10,000Kb to evaluate, identify, and exclude linkage disequilibrium (LD) effects, thereby ensuring the independence of the IVs. To mitigate the potential for horizontal pleiotropy and eliminate confounding variables, we utilized the PhenoScanner V2 database 33. In addition, our choice of IVs also excluded palindromic SNPs to guarantee that the influences on exposure and outcomes correspond to the same alleles. To satisfy another assumption of MR analysis, we utilized the coefficient of determination (R²) as a genetic instrument to quantify the proportion of variance explained for traits, calculated as R² =2MAF×(1-MAF) (β / SD)². The F statistic was employed to assess the existence of weak IV bias and to determine the stability of individual SNPs. When F>10, SNPs are deemed to be undisturbed by weak IV bias, manifesting that the selected SNPs can precisely forecast exposure. The formula is as follows: F= [(K+1-N) R^2^] / [K(R^2^-1)]) 34. K: variants, N: sample size (Fig.1).

### 2.4. Statistical analysis

In this study, inverse variance weighting (IVW) was primarily applied to estimate the relevance between air pollution and neurosurgical multi-system diseases, with weighted median (WM) and MR-Egger methods employed to further assess this correlation 35. Sensitivity analyses incorporated heterogeneity analysis, horizontal pleiotropy analysis, and leave-one-out analysis. Cochran's Q statistic was applied to evaluate the heterogeneity of each MR association. P<0.05 for Cochran's Q statistic indicates heterogeneity exists, necessitating the use of a random effects model as the major method for subsequent testing 36. MR-PRESSO method was assessed using MR-PRESSO outlier tests, corrected level pleiotropy (outlier test), and remarkable discrepancy in causal estimates before and after outlier adjustments 35. Subsequently, a leave-one-out analysis was conducted to assess whether individual SNPs yielded remarkable consequences, thereby systematically excluding confounding SNPs 37. Statistical analyses were proceeded applying R version 4.2.0 and the TwoSampleMR package version 0.6.3. A strong correlation was established when the results remained significant after applying the Bonferroni correction 38. Furthermore, a strong correlation was deemed present if more than two distinct MR analyses yielded P-values less than 0.05. Even in cases where only a single method, particularly the IVW analysis, produced a P<0.05, the correlation was still deemed remarkable.

## 3. Results

### 3.1. MR analysis

To survey the relationship between ambient air pollution and neurosurgical multisystem diseases, we carried out the first global-scale MR analysis. Based on the variations in ambient air pollution, we proceeded with the subsequent analyses.

### 3.2. Causal effects of NOx on neurosurgical multisystem diseases

**Figure 3:**
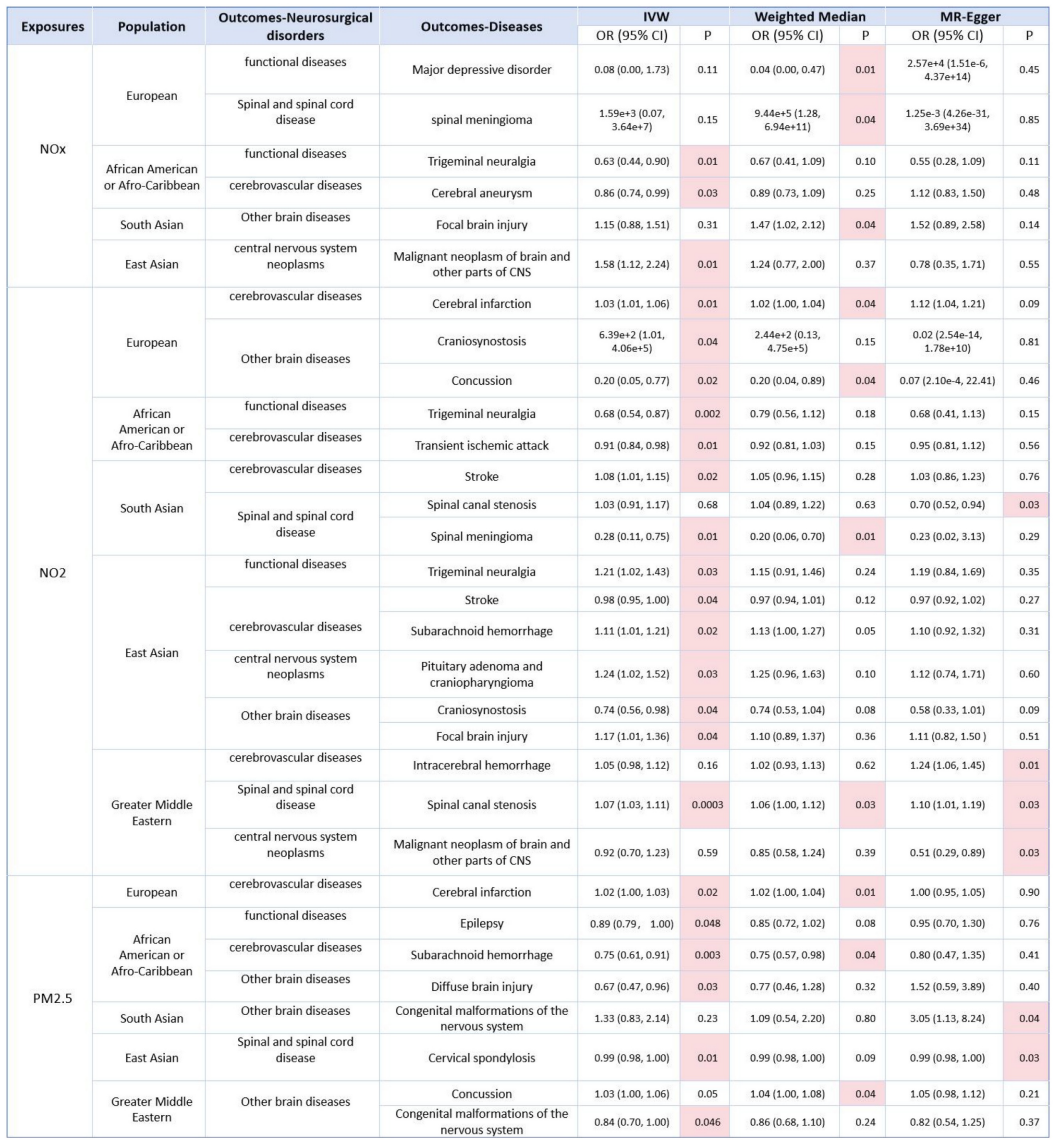
Causality of the risk for air pollution (NOx, NO_2_, PM_2.5_) in the world and Neurosurgical multisystem diseases outcomes (Positive result, p<0.05).

**Figure 4:**
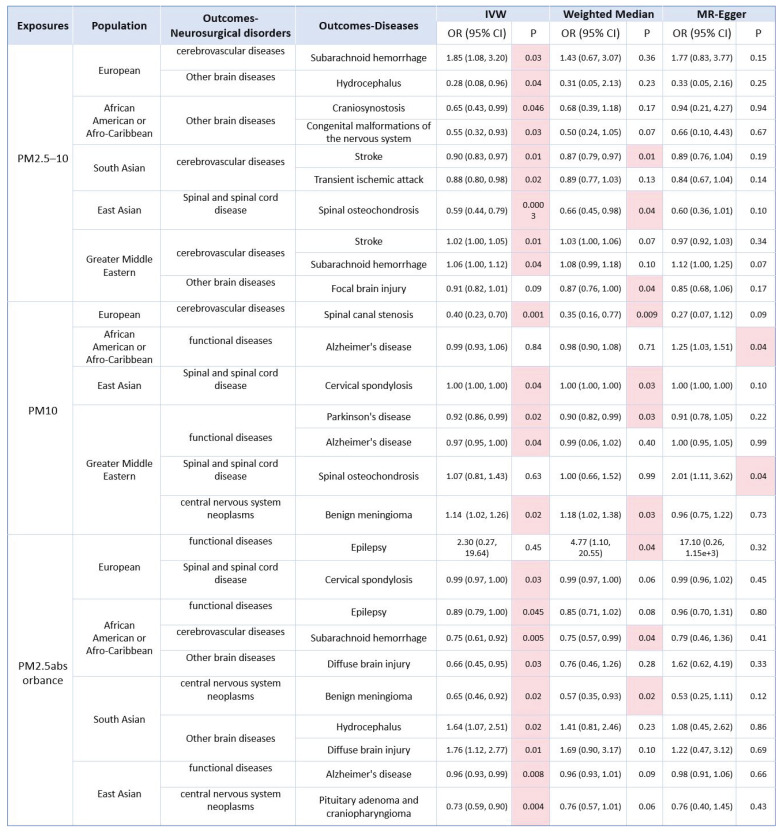
Causality of the risk for air pollution (PM_2.5-10_, PM_10_, PM_2.5_absorbance) in the world and Neurosurgical multisystem diseases outcomes (Positive result, p<0.05).

In the initial IVW, WM, and MR-Egger analyses, we utilized closely related SNPs as genetic instruments for investigating neurosurgical multisystem diseases (Fig.3 and Fig.4). Our consequences revealed a positive relativity between NOx and the dangerousness of spinal meningioma, and a negative relevance with the risk of major depressive disorder in the European population. NOx is negatively associated with the dangerousness of trigeminal neuralgia and cerebral aneurysm in African American or Afro-Caribbean population. NOx is positively correlation with the risk of focal brain injury in South Asian. And NOx is positively correlation with the risk of malignant neoplasm of brain and other parts of CNS in East Asian. However, no causal correlation has been discovered between NOx and other neurosurgical disorders in other populations (Fig.3, Fig.4 and Table S1-S5).

### 3.3. Causal effects of NO2 on neurosurgical multisystem diseases

As for NO_2_, we found that NO_2_ was positively associated with cerebral infarction and craniosynostosis, and negatively correlation with concussion in the European population, where the association between NO_2_ and cerebral infarction and concussion was more robust. NO_2_ was negatively associated with trigeminal neuralgia and transient ischemic attack in African American or Afro-Caribbean population. NO_2_ was positively correlation with stroke, and negatively correlation with spinal canal stenosis and spinal meningioma in the South Asian population, where the association between NO_2_ and spinal meningioma was more robust. As to East Asian population, NO_2_ was positively associated with trigeminal neuralgia (IVW: OR = 1.21, 95%CI: 1.02~1.43, P = 0.03), subarachnoid hemorrhage, pituitary adenoma and craniopharyngioma, focal brain injury, and negatively relevant to stroke and craniosynostosis, where the association between NO_2_ and subarachnoid hemorrhage was more robust. As to Greater Middle Eastern population, NO_2_ was positively associated with intracerebral hemorrhage, spinal canal stenosis, and negatively associated with malignant neoplasm of brain and other parts of CNS, where the relevance between NO_2_ and spinal canal stenosis was more robust. No causal correlation has been found between NO_2_ and other neurosurgical disorders in other populations (Fig.3, Fig.4 and Table S6-S10).

### 3.4. Causal effects of PM2.5 on neurosurgical multisystem diseases

As for PM_2.5_, we discovered that PM_2.5_ was positively correlation with cerebral infarction in the European population, where the association was robust. As for African American or Afro-Caribbean population, PM_2.5_ was negatively associated with epilepsy, subarachnoid hemorrhage, and diffuse brain injury, where the association between PM_2.5_ and subarachnoid hemorrhage was more robust. As for South Asian population, PM_2.5_ was positively associated with congenital malformations of the nervous system. As for East Asian population, PM_2.5_ was negatively associated with cervical spondylosis, where the association was robust. As for Greater Middle Eastern population, PM_2.5_ was positively correlation with concussion, and negatively correlation with congenital malformations of the nervous system. No causal correlation has been discovered between PM_2.5_ and other neurosurgical disorders in other populations (Fig.3, Fig.4 and Table S11-S15).

### 3.5. Causal effects of PM2.5-10 on neurosurgical multisystem diseases

As for PM_2.5-10_, we found that PM_2.5-10_ was positively correlation with subarachnoid hemorrhage, and negatively correlation with hydrocephalus in the European population. In African American or Afro-Caribbean population, PM_2.5-10_ was negatively associated with craniosynostosis and Congenital malformations of the nervous system. As for South Asian population, PM_2.5-10_ was negatively associated with stroke, and transient ischemic attack, where the association between PM_2.5-10_ and stroke was more robust. As for East Asian population, PM_2.5-10_ was negatively correlation with spinal osteochondrosis, where the correlation was robust. As to Greater Middle Eastern population, M_2.5-10_ was positively correlation with stroke and subarachnoid hemorrhage, and negatively correlation with focal brain injury. No causal correlation has been found between PM_2.5-10_ and other neurosurgical disorders in other populations (Fig.3, Fig.4 and Table S16-S20).

### 3.6. Causal effects of PM10 on neurosurgical multisystem diseases

As for PM_10_, we found that PM_10_ was negatively associated with spinal canal stenosis in the European population, where the association was robust. As for African American or Afro-Caribbean population, PM_10_ was positively relevant to Alzheimer's disease. As for East Asian population, PM_10_ was positively associated with cervical spondylosis, where the association was robust. As to Greater Middle Eastern, we found that PM_10_ was negatively associated with Parkinson's disease and Alzheimer's disease, where the association between PM_10_ and Parkinson's disease was more robust. In addition, PM_10_ was positively associated with spinal osteochondrosis and benign meningioma, where the association between PM_10_ and benign meningioma was more robust. No causal correlation has been found between PM_10_ and other neurosurgical disorders in other populations (Fig.3, Fig.4 and Table S21-S24).

### 3.7. Causal effects of PM2.5 absorbance on neurosurgical multisystem diseases

As for PM_2.5_ absorbance, we found that PM_2.5_ absorbance was positively correlation with epilepsy and negatively relevant to cervical spondylosis in the European population. As to African American or Afro-Caribbean population, PM_2.5_ absorbance was negatively associated with epilepsy, subarachnoid hemorrhage and diffuse brain injury, where the correlation between PM_2.5_ absorbance and subarachnoid hemorrhage was more robust. As for South Asian, PM_2.5_ absorbance was negatively relevant to benign meningioma, where the association was robust. Furthermore, PM_2.5_ absorbance was positively associated with hydrocephalus and diffuse brain injury. As for East Asian, we found that PM_2.5_ absorbance was negatively correlation with Alzheimer's disease and Pituitary adenoma and craniopharyngioma. No causal relevance has been found between PM_2.5_ absorbance and other neurosurgical disorders in other populations (Fig.3, Fig.4 and Table S25-S28).

### 3.8. Sensitivity analysis

Sensitivity analysis was used to weigh the dependability of the causal relevance between ambient air pollution and neurosurgical multisystem diseases. This analysis primarily comprised three components: heterogeneity, horizontal pleiotropy, and leave-one-out analysis. Heterogeneity was estimated via Cochran's Q test, P>0.05 manifesting no heterogeneity in the MR analysis. To guarantee the dependability of the conclusions, a random effects model was used for those with P<0.05. Additionally, the MR-Egger intercept was utilized to test for pleiotropy. P<0.05 represents pleiotropy, which showed results of the MR analysis are labile. The research indicates that instability occurs exclusively when there is a relevance between air pollution and neurosurgical diseases using the MR-Egger analysis method, whereas all other results remain robust (Table 2) (Table S29-S56). Overall, the sensitivity analysis conducted in this research corroborates the dependability of the given SNPs selected as genetic instruments, suggesting that air pollution constitutes a dangerous element for neurosurgical multisystem diseases.

## 4. Discussion

In recent years, ambient air pollution has emerged as an increasingly severe threat to human health, contributing to millions of premature deaths globally each year 39. While numerous researches have extensively discovered the relevance between air pollution and the cardiovascular and respiratory systems 40, 41, there remains a paucity of systematic investigations into its causal association with neurosurgical multisystem diseases. Consequently, it is urgently imperative to search underlying risk elements and to develop effective preventive measures aimed at mitigating the disadvantageous health consequences of air pollution. Numerous prospective and observational researches have investigated the correlation between neurological diseases and air pollution across various racial groups. For instance, Ma et al. demonstrated that prolonged PM_2.5_ exposure is relevant to cognitive decline and an elevated dangerousness of Alzheimer's disease, attributed to amyloid accumulation in the brains of Chinese patients 5. Conversely, Zhang et al. proceeded researches on the U.S. population and discovered no significant relevance between PM2.5 exposure and stroke. Furthermore, their findings indicated that stroke did not significantly alter or modulate the causal correlation between PM2.5 exposure and dementia 42. In conclusion, the aforementioned studies did not systematically explore the correlation between air pollution and neurosurgical multisystem diseases, and their findings were devoid of genetic evidence. Consequently, this study tries to address this problem by exploring the potential genetic associations between six air pollution factors—namely PM_2.5_, PM_2.5-10_, PM_10_, PM_2.5_ absorbance, NO_2_ and NOx, and multiple neurological disorders.

This study is distinguished by several key aspects: 1. It presents the inaugural genetic evidence establishing a causal correlation between air pollution and neurosurgical multisystem disease; 2. It identifies specific variations in the impact of air pollution on neurological diseases across different racial and population groups; 3. It posits that a P-value of less than 0.05 in more than two distinct MR analyses indicates a robust correlation. The specific analysis results are as follows: 3.1. NO_2_ exposure is genetically predicted to significantly increase the risk of cerebral infarction in European populations, subarachnoid hemorrhage in East Asian populations, and Spinal canal stenosis in Greater Middle Eastern populations, while reducing the dangerousness of concussion in European populations and spinal meningioma in South Asian populations. 3.2. PM_2.5_ is a dangerousness element for cerebral infarction in European populations, and a protective factor for subarachnoid hemorrhage in African American or Afro-Caribbean populations and cervical spondylosis in East Asian populations. 3.3. PM_2.5-10_ exposure may be negatively related to stroke in South Asian populations and Spinal osteochondrosis in East Asian populations. 3.4. PM_10_ exposure may be positively associated with cervical spondylosis in East Asian populations and benign meningiomas in Greater Middle Eastern populations, but negatively associated with Spinal canal stenosis in European populations and Parkinson's disease in Greater Middle Eastern populations. 3.5. There may be a remarkable negative relevance between PM_2.5_ absorbance and subarachnoid hemorrhage in African American or Afro-Caribbean population and benign meningioma in South Asian population. 4. Air pollution is associated with the following diseases in more than two races and populations: 4.1. NO_2_: trigeminal neuralgia, stroke, spinal stenosis and craniosynostosis; 4.2. PM_2.5_: congenital malformations of the nervous system; 4.3. PM_2.5-10_: subarachnoid hemorrhage and stroke; 4.4. PM_10_: Alzheimer's Disease; 4.5. PM_2.5_ absorbance: epilepsy and diffuse brain injury. 5. From the perspective of different subspecialties of neurosurgery, the diseases associated with air pollution three or more times are as follows: 5.1. functional diseases: trigeminal neuralgia and epilepsy; 5.2. cerebrovascular diseases: stroke and subarachnoid hemorrhage; 5.3. spinal and spinal cord disease: spinal canal stenosis and cervical spondylosis; 5.4. other brain diseases: craniosynostosis, diffuse brain injury, focal brain injury and congenital malformations of the nervous system. 6. The following diseases were not found to be associated with air pollution: obsessive compulsive disorder, intracranial and intraspinal abscess, cervical spinal cord and nerve injuries, glioblastoma, malignant meningioma and benign neoplasm of brain and other parts of CNS.

Regarding functional diseases, this study identified a remarkable negative relevance between trigeminal neuralgia and exposure to NOx and NO_2_ within the African American or Afro-Caribbean population, while a positive association was observed with NO_2_ exposure in the East Asian population. Previous study on the correlation between trigeminal neuralgia and air pollution has not addressed the correlation with NOx and NO_2_, making this study the first to investigate this specific direction. Researchers in China identified no relevance between short-term air pollution exposure and epilepsy 43. Conversely, a retrospective study conducted at a hospital indicated that air pollution, particularly elevated levels of PM_2.5_ and SO_2_, constitutes a risk factor for pediatric convulsions 44. The current study elucidated that PM_2.5_ and PM_2.5_ absorbance are negatively relevant to African American or Afro-Caribbean populations from a genetic perspective. However, the mechanisms by which SNPs influence seizure susceptibility during pollutant exposure remain poorly understood, and there are notable racial disparities in the findings. For Parkinson's disease (PD), a retrospective study conducted by Korean researchers identified a strong relevance between NO_2_ exposure and the incidence and progression of Parkinson's disease (PD) 9. In contrast, a study by Dutch researchers did not establish a remarkable relevance between air pollution exposure and the development of PD among local residents 45. Our study indicates a remarkable relevance between PM_10_ exposure and the occurrence of PD in the Greater Middle Eastern population. These findings could inform the implementation of targeted public health interventions. Numerous researches have demonstrated that PM_2.5_ may elevate the incidence of Alzheimer's disease, potentially through mechanisms involving the reduction of DNA methylation levels, alterations in epigenetic regulation, damage to the CNS, and disruption of intestinal microecological balance 46. The present study identifies a significant correlation between Alzheimer's disease and both PM_10_ and PM_2.5_ absorbance, thereby laying the groundwork for further investigation into the potential associations and underlying mechanisms linking this disease with air pollution.

Regarding cerebrovascular diseases, our study identified a remarkable relevance between NO_2_, PM_2.5_ and an elevated dangerousness of cerebral infarction in European populations. Notably, prior studies have not built a direct correlation between air pollution and cerebral infarction. Consequently, our findings offer novel insights into the etiology and progression of cerebral infarction, particularly within European cohorts. Additionally, existing literature suggests that air pollution exposure may heighten the dangerousness of ischemic stroke, especially among individuals with large artery and small vessel diseases. Short contact air pollution also enhances the risk of hemorrhagic stroke, but the effect of long-term exposure on hemorrhagic risk is unknown 12. Our study identified that various air pollutants exert distinct effects across different ethnic groups. For example, elevated concentrations of NO_2_ were related to an increased dangerousness of stroke in South Asian populations, whereas the same pollutant appeared to have a protective effect in East Asian populations. The underlying mechanisms for these differential effects require further investigation. Hwang et al. demonstrated a significant relevance between air pollution and mortality due to subarachnoid hemorrhage, particularly among female patients. This heightened susceptibility in women may be attributed to their lower smoking rates compared to the general population, as well as anatomical and physiological differences such as smaller airway dimensions, increased airway reactivity, and greater deposition of particulate matter. 47. Our research additionally identified a significant correlation between air pollution—specifically NO_2_, PM_2.5_, and PM_2.5-10_—and the incidence and progression of subarachnoid hemorrhage across nearly all ethnic groups.

Furthermore, we have, for the first time, elucidated a genetic basis for the causal relevance between air pollution and spinal cord diseases, including spinal stenosis and cervical spondylosis. This novel finding offers a foundational framework for future investigations into potential mechanisms and the formulation of preventive and control strategies.

Regarding central nervous system neoplasms, our consequences indicate a potential genetic relevance between air pollution and benign meningiomas. Specifically, we discovered a negative correlation between PM_10_ exposure and meningiomas in Greater Middle Eastern populations, and a positive relevance between PM_2.5_ exposure and meningiomas in South Asian populations. In contrast, Wu et al. conducted a cohort study on air pollution and benign brain tumors and found no remarkable relevance between air pollution and meningioma 48, This discrepancy may be attributed to factors such as racial differences and the limited sample size in their study, and our study was analyzed from a genetic perspective, which made it more credible and convincing. As for malignant brain tumors such as glioma, we have not yet found a genetic link between them and air pollution.

Regarding other neurological disorders, our study concentrated on the causal correlation between air pollution and craniosynostosis, as well as congenital malformations of the nervous system. Congenital malformations represent a significant reason of fetal death, infant mortality and morbidity 49. Our consequences indicate a strong relevance between PM_2.5_, PM_2.5-10_ and congenital malformations of the central nervous system. This is in accordance with previous studies that have been thought have a strong association between PM_10_ exposure during the overall congenital malformations. Subsequent exposure to PM_10_ throughout pregnancy was associated with congenital heart disease, neurodevelopmental abnormalities, and tetralogy of Fallot. In contrast, PM_2.5_ and NO_2_ exposure was not relevant to congenital conditions or malformations 49. Consequently, our study is the first to identify a relevance between PM_2.5_ and PM_2.5-10_ exposure and congenital malformations of the central nervous system, providing significant insights for disease prevention and control. As for craniosynostosis, we also reported it for the first time to provide ideas for follow-up work.

Air pollution has different impacts on the health of people in different regions, which may be caused by population stratification. Population stratification refers to the existence of subgroup structures (such as genetic background, socioeconomic status, race, etc.) in the study population, which leads to the distortion of exposure-disease associations 50. In the study of air pollution, if the population in highly polluted areas also has other high-risk characteristics (such as poverty and poor medical conditions), it may wrongly attribute the health differences entirely to the pollution itself. Low-income areas are usually more polluted, but residents may also face higher stress, poorer diet and medical resources. These factors together exacerbate health risks 51. Some genetic variations (such as GSTP1, TNF-α) may affect an individual's sensitivity to pollution. If the proportion of a specific ethnic group is high in a certain area, differences in "pollution resistance" may be observed 52.

Therefore, population stratification is an important source of deviation, but a comprehensive analysis needs to be conducted in combination with the characteristics of pollutants, climate and medical conditions. The future study design should give priority to controlling socioeconomic status, genetic background, or using instrumental variables (such as policy changes) to reduce confounding.

In this study, a comprehensive MR analysis was performed utilizing data from the GWAS database. The substantial sample size enhances the stability of the causal effect analysis and ensures consistency across various MR analytical methods. Additionally, the application of the MR-Egger analysis method augments the reliability and reproducibility of the investigation into the relevance between air pollution and multi-system diseases within the field of neurosurgery, while mitigating ethical concerns and research costs 53. This study offers compelling genetic basis supporting the causal relevance between air pollution and neurosurgical multi-system diseases, thereby offering an innovative theoretical foundation for the prevention and cure of air pollution-induced neurological conditions. Notably, our study population encompasses data from ethnic groups across multiple regions, enhancing the comprehensiveness and rigor of our conclusions. Although the stabilized of our analysis, it is vital to realize the deficiencies of this study. Firstly, the exposure factors mainly selected in this study are PM_2.5_, PM_2.5-10_, PM_10_, PM_2.5_ absorbance, NO_2_ and NOx, so there is a lack of research on some specific air pollutants, such as O_3_, NO and SO_2_, so this study cannot provide more detailed analytical data to determine their impact on neurosurgical multisystem diseases. Furthermore, a fundamental assumption of MR analysis is the linear correlation between genetic variation, exposure factors, and outcomes. However, prior epidemiological studies indicate a nonlinear correlation between air pollution and disease 54. Consequently, our study must rigorously account for potential nonlinear relationships or time-varying effects during the MR analysis. Despite certain drawbacks, our MR analysis yields valuable understandings into the genetic relevance between air pollution and neurosurgical multisystem disease. We believe that based on the consequences of this study, people will have a deeper insight of the underlying function of air pollution in neurological diseases in the future, so as to develop more effective prevention and control measures.

## 5. Recommendations for Future Investigations

Given the established causal correlation between ambient air pollution and neurosurgical multisystem diseases, several recommendations for future study are proposed. Firstly, it is imperative to elucidate the potential mechanisms through which air pollution interact with genetic elements, thereby increasing the dangerousness of neurosurgical multisystem diseases. This line of inquiry aims to identify and characterize the critical susceptibility windows for neurological diseases and to pinpoint underlying targets for intervention. Second, investigate the underlying interactions between air pollutants and genetic variants across different populations to determine whether specific genetic variants are more likely to be associated with particular neurological disorders. Third, as evidenced by this study, it is crucial to validate the causal correlation between air pollution and neurological diseases using data from multiple regions, ethnic groups, and datasets. Finally, large-scale prospective cohort studies will be essential to gather and monitor the long-term exposure of different populations to air pollution to build a more clearly causal correlation between exposure factors and outcomes in the future.

## 6. Conclusions

In conclusion, our study elucidates the environmental and societal ramifications of mitigating air pollution to diminish the dangerousness of multisystem diseases pertinent to neurosurgery. The implementation of robust air pollution prevention and cure way, the establishment of sustainable urban planning frameworks, the propelling of clean energy strategies, and the adoption of science-based occupational safeguards are pivotal in safeguarding public health. These consequences furnish critical evidence for policymakers, healthcare professionals, environmental organizations, and the general populace to advocate for and execute air quality enhancement initiatives on a global scale. Ultimately, it will benefit the whole world and improve human health.

## Supplementary Material

Supplementary tables.

## Figures and Tables

**Table 1:** Details of GWAS in the present study (outcome).

Characteristics		Consortium	GWAS ID	Sample size	Number of SNPs	Population	Sex	Year
Neurosurgical disorders	Diseases							
functional diseases	Trigeminal neuralgia	NA	finn-b-G6_TRINEU	195,847	16,380,408	European	Males and females	2021
	Epilepsy	NA	ebi-a-GCST90018840	458,310	24,186,492	European	NA	2021
	Parkinson's disease	NA	ebi-a-GCST90018894	480,018	24,194,622	European	NA	2021
	Alzheimer's disease	NA	ebi-a-GCST90027158	487,511	20,921,626	European	NA	2022
	Major depressive disorder	NA	ebi-a-GCST90086059	56,637	11,498,420	European	NA	2021
	Obsessive Compulsive Disorder	PGC	ieu-a-1189	33,925	8,409,517	European	Males and females	2017
cerebrovascular diseases	Stroke	NA	ebi-a-GCST005838	446,696	7,633,440	European	NA	2018
	Intracerebral hemorrhage	NA	ebi-a-GCST90018870	473,513	24,191,284	European	NA	2021
	Subarachnoid hemorrhage	NA	ebi-a-GCST90018923	473,255	24,191,735	European	NA	2021
	Transient ischemic attack	NA	finn-b-G6_TIA	214,634	16,380,437	European	Males and females	2021
	Cerebral infarction	NA	ukb-d-I63	361,194	10,889,323	European	Males and females	2018
	Cerebral aneurysm	NA	ebi-a-GCST90018815	473,683	24,191,145	European	NA	2021
Spinal and spinal cord diseases	Cervical spondylosis	NA	ebi-a-GCST90038693	484,598	9,587,836	European	NA	2021
	Spinal canal stenosis	NA	ebi-a-GCST90018922	454,787	24,182,979	European	NA	2021
	Spinal meningioma	NA	finn-b-CD2_BENIGN_MENINGES_SPINAL	218,792	16,380,466	European	Males and females	2021
	Spinal osteochondrosis	NA	finn-b-M13_SPINALOSTEOCHON	164,865	16,380,216	European	Males and females	2021
	Intracranial and intraspinal abscess	NA	finn-b-G6_CNSABSC	217,626	16,380,461	European	Males and females	2021
	Cervical spinal cord and nerve injuries	NA	finn-b-ST19_INJURY_NERVES_SPINAL_CORD_NECK_LEVEL	215,730	16,380,463	European	Males and females	2021
central nervous system neoplasms	Glioblastoma	NA	finn-b-C3_GBM	218,792	16,380,466	European	Males and females	2021
	Benign meningioma	NA	finn-b-CD2_BENIGN_MENINGES_CEREBRAL	218,792	16,380,466	European	Males and females	2021
	Malignant meningioma	NA	finn-b-C3_MENINGES	218,792	16,380,466	European	Males and females	2021
	Pituitary adenoma and craniopharyngioma	NA	finn-b-CD2_BENIGN_PITUITARY_CRANIPHAR	218,792	16,380,466	European	Males and females	2021
	Benign neoplasm of brain and other parts of CNS	NA	finn-b-CD2_BENIGN_BRAIN_CNS	218,792	16,380,466	European	Males and females	2021
	Malignant neoplasm of brain and other parts of CNS	NA	finn-b-C3_SPINAL_CORD_CRANIAL_AND_OTHER_CNS	218,792	16,380,466	European	Males and females	2021
Other brain diseases	Hydrocephalus	NA	finn-b-G6_HYDROCEPH	206,548	16,380,404	European	Males and females	2021
	Craniosynostosis	NA	finn-b-Q17_CRANIOSYNOSTOSIS	218,792	16,380,466	European	Males and females	2021
	Concussion	NA	finn-b-ST19_CONCUSSION	147,103	16,380,074	European	Males and females	2021
	Diffuse brain injury	NA	finn-b-ST19_DIFFU_BRAIN_INJURY	137,232	16,379,965	European	Males and females	2021
	Focal brain injury	NA	finn-b-ST19_FOCAL_BRAIN_INJURY	137,641	16,379,970	European	Males and females	2021
	Congenital malformations of the nervous system	NA	finn-b-Q17_CONGEN_MALFO_NERVOUS_SYSTEM	218,792	16,380,466	European	Males and females	2021

**Table 2:** Sensitivity analyses of MR-Egger intercept regression and Cochrane Q tests (Positive result).

Exposures	Population	Outcomes-Neurosurgical disorders	Outcomes-Diseases	Q_MR.Egger	Q_df_MR.Egger	Q_pval	Q_IVW	Q_df_IVW	Q_pval	Egger_intercept	se	pval
NOx	European	functional diseases	major depressive disorder	11.11	4	0.03	14.26	5	0.01	-0.1682	0.1582	0.3475
		spinal and spinal cord disease	spinal meningioma	4.88	4	0.30	5.12	5	0.40	0.2027	0.4585	0.6812
	African American or Afro-Caribbean	functional diseases	trigeminal neuralgia	6.38	15	0.97	6.61	16	0.98	0.0173	0.0361	0.6386
		cerebrovascular diseases	cerebral aneurysm	16.70	18	0.54	20.65	19	0.36	-0.0331	0.0166	0.0624
	South Asian	other brain diseases	focal brain injury	17.23	16	0.37	18.73	17	0.34	-0.0366	0.0310	0.2544
	East Asian	central nervous system neoplasms	malignant neoplasm of brain and other parts of cns	3.69	8	0.88	7.50	9	0.58	0.1753	0.0896	0.0862
NO_2_	European	cerebrovascular diseases	cerebral infarction	1.67	2	0.43	6.84	3	0.08	-0.0014	0.0006	0.1508
		other brain diseases	craniosynostosis	0.16	2	0.92	0.73	3	0.87	0.1699	0.2244	0.5279
		other brain diseases	concussion	0.59	2	0.74	0.73	3	0.87	0.175	0.0472	0.7466
	African American or Afro-Caribbean	functional diseases	trigeminal neuralgia	15.33	25	0.93	15.33	26	0.95	0.0002	0.0271	0.9956
		cerebrovascular diseases	transient ischemic attack	20.36	25	0.73	20.79	26	0.75	-0.0057	0.0086	0.5155
	South Asian	cerebrovascular diseases	stroke	12.10	16	0.74	12.41	17	0.77	0.0041	0.0073	0.5857
		spinal and spinal cord disease	spinal canal stenosis	22.31	21	0.38	30.18	22	0.11	0.0341	0.0125	0.0128
		spinal and spinal cord disease	spinal meningioma	10.36	18	0.92	10.38	19	0.94	0.0184	0.1102	0.8693
	East Asian	functional diseases	trigeminal neuralgia	5.65	9	0.77	5.65	10	0.84	0.0035	0.0415	0.9355
		cerebrovascular diseases	stroke	9.14	10	0.52	9.24	11	0.60	0.0022	0.0069	0.7603
		cerebrovascular diseases	subarachnoid hemorrhage	12.85	12	0.38	12.85	13	0.46	0.0010	0.0214	0.9640
		central nervous system neoplasms	pituitary adenoma and craniopharyngioma	12.07	9	0.21	12.47	10	0.25	0.0273	0.0501	0.5986
		other brain diseases	craniosynostosis	12.08	9	0.21	13.42	10	0.20	0.0673	0.0673	0.3437
		other brain diseases	focal brain injury	8.54	9	0.48	8.68	10	0.56	0.0135	0.0362	0.7174
	Greater Middle Eastern	cerebrovascular diseases	intracerebral hemorrhage	14.91	22	0.87	20.38	23	0.62	-0.0515	0.0220	0.0289
		spinal and spinal cord disease	spinal canal stenosis	17.14	22	0.76	17.71	23	0.77	-0.0085	0.0112	0.4576
		central nervous system neoplasms	malignant neoplasm of brain and other parts of cns	18.56	18	0.42	24.40	19	0.18	0.1918	0.0806	0.0285
PM2.5	European	cerebrovascular diseases	cerebral infarction	2.92	3	0.40	3.38	4	0.50	0.0002	0.0004	0.5486
	African American or Afro-Caribbean	functional diseases	epilepsy	20.18	22	0.57	20.39	23	0.62	-0.0061	0.0134	0.6543
		cerebrovascular diseases	subarachnoid hemorrhage	28.53	22	0.16	28.62	23	0.19	-0.0061	0.0230	0.7916
		other brain diseases	diffuse brain injury	19.83	21	0.53	23.20	22	0.39	-0.0759	0.0414	0.0805
	South Asian	other brain diseases	congenital malformations of the nervous system	19.82	24	0.71	23.26	25	0.56	-0.0929	0.0501	0.0760
	East Asian	spinal and spinal cord disease	cervical spondylosis	11.42	11	0.41	13.37	12	0.34	0.0002	0.0001	0.1987
	Greater Middle Eastern	other brain diseases	concussion	15.63	26	0.94	15.91	27	0.95	-0.0058	0.0110	0.6040
		other brain diseases	congenital malformations of the nervous system	20.45	26	0.77	20.46	27	0.81	0.0062	0.0663	0.9265
PM2.5-10	European	cerebrovascular diseases	subarachnoid hemorrhage	39.77	39	0.44	39.80	40	0.48	0.0017	0.0094	0.8589
		other brain diseases	hydrocephalus	32.60	36	0.63	32.65	37	0.67	-0.0046	0.0199	0.8173
	African American or Afro-Caribbean	other brain diseases	craniosynostosis	7.91	17	0.97	8.15	18	0.98	-0.0403	0.0812	0.6265
		other brain diseases	congenital malformations of the nervous system	13.89	17	0.67	13.93	18	0.73	-0.0203	0.1025	0.8451
	South Asian	cerebrovascular diseases	stroke	8.28	8	0.41	8.30	9	0.50	0.0015	0.0098	0.8780
		cerebrovascular diseases	transient ischemic attack	3.77	10	0.96	4.07	11	0.97	0.0101	0.0183	0.5926
	East Asian	spinal and spinal cord disease	spinal osteochondrosis	9.83	7	0.20	9.84	8	0.28	-0.0085	0.0870	0.9247
	Greater Middle Eastern	cerebrovascular diseases	stroke	16.73	21	0.73	20.81	22	0.53	0.0129	0.0064	0.0562
		cerebrovascular diseases	subarachnoid hemorrhage	20.84	25	0.70	21.92	26	0.69	-0.0167	0.0161	0.3093
		other brain diseases	focal brain injury	34.69	23	0.06	35.40	24	0.06	0.0213	0.0312	0.5008
PM10	European	cerebrovascular diseases	spinal canal stenosis	16.29	18	0.57	16.63	19	0.61	0.0080	0.0137	0.5650
	African American or Afro-Caribbean	functional diseases	alzheimer's disease	7.90	12	0.79	13.83	13	0.39	-0.0238	0.0098	0.0315
	East Asian	spinal and spinal cord disease	cervical spondylosis	3.18	8	0.92	4.30	9	0.89	-0.0002	0.00002	0.3194
	Greater Middle Eastern	functional diseases	parkinson's disease	14.17	14	0.44	14.22	15	0.51	0.0043	0.0182	0.8176
		functional diseases	alzheimer's disease	17.73	14	0.22	19.47	15	0.19	-0.0071	0.0060	0.2615
		spinal and spinal cord disease	spinal osteochondrosis	12.04	14	0.60	17.48	15	0.29	-0.1668	0.0715	0.0351
		central nervous system neoplasms	benign meningioma	9.05	14	0.83	11.50	15	0.72	0.0453	0.0289	0.1396
PM2.5absorbance	European	functional diseases	epilepsy	5.97	2	0.05	9.43	3	0.02	-0.0618	0.0574	0.3941
		spinal and spinal cord disease	cervical spondylosis	0.18	2	0.91	0.19	3	0.98	-0.0004	0.0003	0.9173
	African American or Afro-Caribbean	functional diseases	epilepsy	20.00	21	0.52	20.28	22	0.57	-0.0071	0.0136	0.6078
		cerebrovascular diseases	subarachnoid hemorrhage	28.45	21	0.13	28.51	22	0.16	-0.0051	0.0238	0.8310
		other brain diseases	diffuse brain injury	18.85	20	0.53	22.86	21	0.35	-0.0847	0.0423	0.0590
	South Asian	central nervous system neoplasms	benign meningioma	13.87	13	0.38	14.27	14	0.43	0.0244	0.0399	0.5524
		other brain diseases	hydrocephalus	4.14	13	0.99	5.25	14	0.98	0.0501	0.0478	0.3130
		other brain diseases	diffuse brain injury	8.61	13	0.80	9.38	14	0.81	0.0448	0.0510	0.3957
	East Asian	functional diseases	alzheimer's disease	7.06	9	0.63	7.52	10	0.68	-0.0056	0.0082	0.5161
		central nervous system neoplasms	pituitary adenoma and craniopharyngioma	2.35	9	0.98	2.37	10	0.99	-0.0106	0.0637	0.8720
