# Supplementary material for: Causal Effects of Exposure to Air Pollution on the Risk of Neurosurgical Multi-system Diseases: A Worldwide Study of Mendelian Randomization
Source: Int J Med Sci. 2025 Jul 28;22(14):3565–80. doi: 10.7150/ijms.115853 (PMC12434832; doi:10.7150/ijms.115853)
Supplement: Supplementary file 1 — Supplementary tables. [file ijmsv22p3565s1.pdf]

Table S1 Causality of the risk for NOx in European and Neurosurgical multisystem diseases outcomes ($p < 1 \times 10^{-5}$.).

Outcome		IVW		Weighted Median		MR-Egger	
		OR (95% CI)	P	OR (95% CI)	P	OR (95% CI)	P
NOx European	Trigeminal neuralgia	0.81 (0.01, 4.67)	0.92	0.18 (0.00, 2.96)	0.51	27.92 (0.00, 5.70e+12)	0.81
	Epilepsy	0.41 (0.06, 2.91)	0.37	1.08 (0.14, 8.36)	0.93	46.15 (0.00, 2.40e+6)	0.53
	Parkinson's disease	1.14 (0.16, 7.97)	0.90	1.83 (0.15, 22.82)	0.64	30.76 (0.00, 3.17e+5)	0.51
	Alzheimer's disease	1.64 (0.92, 2.91)	0.09	1.99 (0.97, 4.08)	0.06	4.62 (0.41, 52.08)	0.82
	Major depressive disorder	0.08 (0.00, 1.73)	0.11	0.04 (0.00, 0.47)	0.01	2.57e+4 (1.51e-6, 4.37e+14)	0.45
	Obsessive Compulsive Disorder	0.73 (0.07, 8.20)	0.80	0.96 (0.05, 18.41)	0.98	0.00 (3.39e-8, 82.37)	0.31
	Stroke	0.71 (0.39, 1.29)	0.26	0.91 (0.44, 1.91)	0.81	0.54 (0.04, 8.36)	0.68
	Intracerebral hemorrhage	0.60 (0.06, 6.28)	0.67	0.81 (0.05, 12.24)	0.88	589.43 (0.01, 3.84e+7)	0.32
	Subarachnoid hemorrhage	8.20 (0.19, 363.54)	0.28	18.25 (0.71, 466.30)	0.08	7.75e+6 (0.99, 6.05e+13)	0.12
	Transient ischemic attack	0.30 (0.03, 2.67)	0.28	0.26 (0.04, 1.87)	0.18	0.82 (5.38e-7, 1.26e+6)	0.98
	Cerebral infarction	1.01 (1.00, 1.03)	0.07	1.01 (0.99, 1.03)	0.39	0.97 (0.92, 1.03)	0.39
	Cerebral aneurysm	0.45 (0.01, 15.05)	0.65	0.90 (0.03, 24.47)	0.95	0.06 (3.44e-11, 1.17e+8)	0.81
	Cervical spondylosis	0.99 (0.97, 1.00)	0.06	0.99 (0.97, 1.00)	0.10	1.03 (0.97, 1.09)	0.36
	Spinal canal stenosis	1.81 (0.61, 5.35)	0.28	2.49 (0.59, 10.45)	0.21	7.59 (0.04, 1394.37)	0.49
	spinal meningioma	1.59e+3 (0.07, 3.64e+7)	0.15	9.44e+5 (1.28, 6.94e+11)	0.04	1.25e-3 (4.26e-31, 3.69e+34)	0.85
	Spinal osteochondrosis	2.54 (4.75e-7, 1.35e+4)	0.83	133.78 (2.28e-3, 7.85e+6)	0.38	8.30e+7 (5.52e-16, 1.25e+31)	0.54

	Intracranial and intraspinal abscess	6.66 (7.37e-4, 6.03e+4)	0.68	0.02 (7.73e-8, 3.82e+3)	0.52	5.10e+14 (4.38e-9, 5.94e+37)	0.28
	Cervical spinal cord and nerve injuries	150.19 (0.17, 1.31e+5)	0.15	864.21 (0.11, 6.51e+6)	0.14	7.28e+5 (4.21e-12, 1.26e+23)	0.54
	Glioblastoma	39.01 (5.04e-4, 3.02e+6)	0.52	16.36 (1.44e-5, 1.86e+7)	0.69	4.88e+6 (1.99e-22, 1.20e+35)	0.67
	Benign meningioma	4.52 (0.18, 112.85)	0.36	1.55 (0.03, 93.79)	0.83	4176.11 (3.14e-5, 5.55e+11)	0.43
	Malignant meningioma	18.66 (0.23, 1.51e+3)	0.19	7.45 (0.03, 1.91e+3)	0.48	3.03e+4 (3.04e-8, 3.02e+16)	0.50
	Pituitary adenoma and craniopharyngioma	0.43 (3.57e-3, 52.46)	0.73	2.09 (8.38e-3, 519.19)	0.79	9.39e-8 (1.38e-19, 6.38e+4)	0.31
	Benign neoplasm of brain and other parts of CNS	0.62 (7.19e-3, 53.79)	0.83	2.47 (0.02, 3.32e+2)	0.72	0.02 (6.21e-15, 6.87e+10)	0.81
	Malignant neoplasm of brain and other parts of CNS	0.02 (1.02e-15, 45.42)	0.33	0.01 (1.04e-6, 2.01e+2)	0.38	0.27 (1.48e-20, 4.92e+18)	0.96
	Hydrocephalus	2.70 (5.07e-2, 144.11)	0.62	0.95 (6.68e-3, 135.84)	0.98	6.05e-4 (4.36e-14, 8.40e+6)	0.57
	Craniosynostosis	0.09 (3.64e-4, 24.10)	0.40	0.15 (1.26e-4, 1.72e+2)	0.59	0.00 (6.78e-20, 6.54e+11)	0.67
	Concussion	0.83 (0.27, 2.57)	0.75	0.92 (0.22, 3.78)	0.90	17.80 (0.02, 1.30e+4)	0.44
	Diffuse brain injury	1.59 (0.01, 1.92e+2)	0.85	0.69 (2.87e-3, 1.68e+2)	0.90	6.24e-6 (1.61e-18, 2.42e+7)	0.46
	Focal brain injury	0.69 (2.43e-2, 19.37)	0.82	0.82 (0.01, 51.63)	0.93	0.03 (7.43e-11, 8.66e+6)	0.73
	Congenital malformations of the nervous system	1.81 (2.05e-3, 1.60e+3)	0.86	18.71 (4.86e-3, 7.20e+4)	0.49	1.68e-8 (1.09e-25, 2.57e+9)	0.43

Table S2 Causality of the risk for NOx in African American or

Afro-Caribbean and Neurosurgical multisystem diseases outcomes ($p < 1 \times 10^{-5}$.).

Outcome		IVW		Weighted Median		MR-Egger	
		OR (95% CI)	P	OR (95% CI)	P	OR (95% CI)	P
NOx African American or Afro-Caribbean	Trigeminal neuralgia	0.63 (0.44, 0.90)	0.01	0.67 (0.41, 1.09)	0.10	0.55 (0.28, 1.09)	0.11
	Epilepsy	1.01 (0.91, 1.13)	0.82	1.02 (0.87, 1.20)	0.78	1.13 (0.90, 1.41)	0.31
	Parkinson's disease	1.09 (0.93, 1.28)	0.31	1.16 (0.93, 1.45)	0.18	1.22 (0.89, 1.68)	0.23
	Alzheimer's disease	0.98 (0.92, 1.03)	0.42	0.97 (0.91, 1.05)	0.47	1.03 (0.93, 1.14)	0.58
	Major depressive disorder	0.73 (0.07, 8.20)	0.80	0.96 (0.05, 18.41)	0.98	1.23 (0.75, 2.02)	0.43
	Obsessive Compulsive Disorder	1.00 (0.76, 1.30)	0.98	1.09 (0.81, 1.47)	0.56	1.17 (0.69, 1.97)	0.57
	Stroke	1.02 (0.97, 1.07)	0.46	1.00 (0.93, 1.07)	0.99	0.95 (0.87, 1.04)	0.31
	Intracerebral hemorrhage	0.90 (0.77, 1.05)	0.17	0.99 (0.80, 1.22)	0.92	1.02 (0.75, 1.38)	0.92
	Subarachnoid hemorrhage	0.88 (0.74,1.05)	0.16	0.84 (0.66, 1.07)	0.16	0.80 (0.55, 1.16)	0.25
	Transient ischemic attack	1.12 (0.99, 1.26)	0.06	1.13 (0.96, 1.32)	0.15	1.05 (0.83, 1.32)	0.70
	Cerebral infarction	1.00 (1.00, 1.00)	0.63	1.00 (1.00, 1.00)	0.27	1.00 (1.00, 1.00)	0.88
	Cerebral aneurysm	0.86 (0.74, 0.99)	0.03	0.89 (0.73, 1.09)	0.25	1.12 (0.83, 1.50)	0.48
	Cervical spondylosis	1.00 (1.00, 1.00)	0.28	1.00 (1.00, 1.00)	0.17	1.00 (1.00, 1.00)	0.71
	Spinal canal stenosis	0.99 (0.89, 1.10)	0.89	1.02 (0.89, 1.17)	0.80	1.01 (0.82, 1.26)	0.90
	spinal meningioma	1.48 (0.59, 3.74)	0.40	1.27 (0.36, 4.48)	0.71	1.25 (0.22, 7.24)	0.81
	Spinal osteochondrosis	1.35 (0.64, 2.84)	0.43	1.42 (0.47, 4.25)	0.53	2.82 (0.68, 11.71)	0.17
	Intracranial and intraspinal abscess	0.81 (0.31, 2.14)	0.67	0.41 (0.12, 1.39)	0.15	0.29 (0.05, 1.79)	0.20
	Cervical spinal cord and nerve injuries	1.06 (0.56, 1.99)	0.86	1.40 (0.59, 3.32)	0.44	2.31 (0.69, 7.75)	0.19
	Glioblastoma	0.61 (0.21, 1.74)	0.36	0.31 (0.07, 1.42)	0.13	0.54 (0.07, 3.98)	0.55
	Benign meningioma	0.91 (0.68,	0.55	0.93 (0.62,	0.75	0.91 (0.51,	0.76

		1.23)		1.42)		1.62)	
	Malignant meningioma	1.14 (0.76, 1.72)	0.53	1.41 (0.81, 2.44)	0.23	1.01 (0.45, 2.28)	0.98
	Pituitary adenoma and craniopharyngioma	1.19 (0.77, 1.86)	0.43	1.52 (0.85, 2.74)	0.16	1.25 (0.52, 2.98)	0.63
	Benign neoplasm of brain and other parts of CNS	0.99 (0.71, 1.38)	0.96	1.14 (0.72, 1.80)	0.59	1.24 (0.66, 2.35)	0.51
	Malignant neoplasm of brain and other parts of CNS	1.56 (0.76, 3.18)	0.22	1.28 (0.47, 3.49)	0.62	2.21 (0.57, 8.63)	0.27
	Hydrocephalus	0.92 (0.63, 1.33)	0.65	0.89 (0.53, 1.51)	0.67	1.18 (0.58, 2.41)	0.65
	Craniosynostosis	0.69 (0.42, 1.14)	0.15	0.73 (0.37, 1.46)	0.38	0.61 (0.23, 1.58)	0.32
	Concussion	1.00 (0.88, 1.14)	0.99	0.97 (0.82, 1.13)	0.67	0.98 (0.77, 1.26)	0.90
	Diffuse brain injury	0.86 (0.51, 1.45)	0.56	0.60 (0.35, 1.03)	0.06	0.41 (0.16, 1.04)	0.08
	Focal brain injury	0.98 (0.65, 1.49)	0.93	1.24 (0.80, 1.93)	0.33	1.30 (0.58, 2.92)	0.53
	Congenital malformations of the nervous system	0.65 (0.33, 1.26)	0.20	0.96 (0.40, 2.29)	0.93	0.51 (0.14, 1.89)	0.33

Table S3 Causality of the risk for NOx in South Asian and Neurosurgical

multisystem diseases outcomes ($p < 1 \times 10^{-5}$.).

Outcome		IVW		Weighted Median		MR-Egger	
		OR (95% CI)	P	OR (95% CI)	P	OR (95% CI)	P
NOx South Asian	Trigeminal neuralgia	0.95 (0.70, 1.28)	0.73	0.93 (0.63, 1.37)	0.71	1.18 (0.65, 2.13)	0.59
	Epilepsy	1.04 (0.93, 1.16)	0.46	1.08 (0.93, 1.26)	0.33	1.11 (0.88, 1.41)	0.38
	Parkinson's disease	1.05 (0.89, 1.24)	0.54	1.00 (0.79, 1.25)	0.97	0.89 (0.65, 1.24)	0.51
	Alzheimer's disease	1.01 (0.96, 1.06)	0.81	0.99 (0.93, 1.07)	0.89	0.97 (0.88, 1.08)	0.08
	Major depressive disorder	0.91 (0.75, 1.11)	0.36	0.92 (0.70, 1.21)	0.55	0.81 (0.44, 1.50)	0.51
	Obsessive Compulsive Disorder	0.92 (0.71, 1.18)	0.49	1.01 (0.73, 1.41)	0.94	1.41 (0.88, 2.25)	0.17
	Stroke	1.01 (0.94, 1.08)	0.81	0.98 (0.90, 1.06)	0.55	0.97 (0.85, 1.12)	0.70
	Intracerebral hemorrhage	1.11 (0.95, 1.31)	0.19	1.00 (0.80, 1.24)	0.97	0.88 (0.63, 1.24)	0.48
	Subarachnoid hemorrhage	1.06 (0.89, 1.27)	0.51	1.00 (0.79, 1.26)	0.97	1.12 (0.76, 1.66)	0.57
	Transient ischemic attack	1.06 (0.95, 1.17)	0.30	1.01 (0.88, 1.17)	0.84	0.99 (0.81, 1.21)	0.92
	Cerebral infarction	1.00 (1.00, 1.00)	0.38	1.00 (1.00, 1.00)	0.89	1.00 (1.00, 1.00)	0.57
	Cerebral aneurysm	1.04 (0.89, 1.22)	0.62	1.05 (0.84, 1.31)	0.67	1.34 (0.93, 1.94)	0.13
	Cervical spondylosis	1.00 (1.00, 1.00)	0.22	1.00 (1.00, 1.00)	0.26	1.00 (1.00, 1.00)	0.56
	Spinal canal stenosis	1.02 (0.90, 1.15)	0.81	1.02 (0.88, 1.20)	0.76	1.08 (0.83, 1.39)	0.58
	spinal meningioma	1.25 (0.57, 2.72)	0.57	1.18 (0.41, 3.39)	0.76	2.55 (0.55, 11.83)	0.25
	Spinal osteochondrosis	1.37 (0.74, 2.57)	0.32	0.99 (0.41, 2.43)	0.99	0.57 (0.16, 1.95)	0.38
	Intracranial and intraspinal abscess	1.37 (0.67, 2.79)	0.38	0.97 (0.36, 2.63)	0.95	0.83 (0.20, 3.35)	0.79
	Cervical spinal cord and nerve injuries	1.35 (0.79, 2.29)	0.27	1.28 (0.63, 2.57)	0.50	1.09 (0.38, 3.12)	0.87
	Glioblastoma	0.76 (0.31, 1.83)	0.53	1.33 (0.40, 4.48)	0.64	1.12 (0.20, 6.44)	0.90
	Benign meningioma	1.03 (0.80, 1.33)	0.80	0.89 (0.62, 1.27)	0.52	0.91 (0.55, 1.49)	0.71

	Malignant meningioma	1.21 (0.85, 1.71)	0.29	1.26 (0.76, 2.07)	0.38	1.01 (0.50, 2.05)	0.97
	Pituitary adenoma and craniopharyngioma	0.84 (0.61, 1.15)	0.27	0.83 (0.53, 1.29)	0.40	0.98 (0.53, 1.81)	0.94
	Benign neoplasm of brain and other parts of CNS	0.89 (0..68, 1.18)	0.43	0.90 (0.60, 1.34)	0.59	1.01 (0.58, 1.75)	0.97
	Malignant neoplasm of brain and other parts of CNS	1.07 (0.48, 2.36)	0.87	1.18 (0.45, 3.05)	0.74	0.97 (0.19, 4.86)	0.97
	Hydrocephalus	1.14 (0.78, 1.67)	0.50	1.00 (0.63, 1.58)	0.99	1.23 (0.56, 2.69)	0.61
	Craniosynostosis	1.19 (0.78, 1.81)	0.41	1.09 (0.60, 1.99)	0.78	1.14 (0.50, 2.60)	0.76
	Concussion	1.05 (0.95, 1.15)	0.38	1.06 (0.93, 1.20)	0.41	1.06 (0.87, 1.30)	1.06
	Diffuse brain injury	0.89 (0.64, 1.23)	0.47	0.99 (0.61, 1.60)	0.97	1.26 (0.66, 2.42)	0.49
	Focal brain injury	1.15 (0.88, 1.51)	0.31	1.47 (1.02, 2.12)	0.04	1.52 (0.89, 2.58)	0.14
	Congenital malformations of the nervous system	1.04 (0.62, 1.77)	0.87	1.54 (0.73, 3.26)	0.26	0.99 (0.35, 2.79)	0.98

Table S4 Causality of the risk for NOx in East Asian and Neurosurgical multisystem diseases outcomes ($p < 1 \times 10^{-5}$.).

Outcome		IVW		Weighted Median		MR-Egger	
		OR (95% CI)	P	OR (95% CI)	P	OR (95% CI)	P
NOx East Asian	Trigeminal neuralgia	1.02 (0.86, 1.21)	0.84	1.13 (0.89, 1.43)	0.31	1.37 (0.93, 2.04)	0.15
	Epilepsy	1.03 (0.96, 1.10)	0.47	1.01 (0.92, 1.10)	0.89	0.95 (0.80, 1.12)	0.53
	Parkinson's disease	0.99 (0.90, 1.10)	0.87	1.02 (0.89,1.17)	0.76	0.82 (0.66, 1.03)	0.12
	Alzheimer's disease	0.98 (0.95, 1.01)	0.16	0.98 (0.94, 1.02)	0.34	0.98 (0.91, 1.05)	0.52
	Major depressive disorder	0.97 (0.90, 1.06)	0.53	1.02 (0.91, 1.15)	0.73	1.13 (0.90, 1.42)	0.33
	Obsessive Compulsive Disorder	1.05 (0.91, 1.22)	0.51	0.99 (0.82, 1.19)	0.91	1.07 (0.75, 1.53)	0.72
	Stroke	1.01 (0.96, 1.05)	0.83	1.02 (0.98, 1.07)	0.26	1.07 (0.96, 1.19)	0.27
	Intracerebral hemorrhage	1.06 (0.96, 1.17)	0.22	1.08 (0.94, 1.23)	0.28	1.14 (0.89, 1.46)	0.32
	Subarachnoid hemorrhage	0.97 (0.83, 1.13)	0.71	0.93 (0.78, 1.10)	0.41	0.71 (0.51, 0.99)	0.07
	Transient ischemic attack	1.05 (0.98, 1.13)	0.15	1.05 (0.97, 1.13)	0.22	1.11 (0.94, 1.31)	0.25
	Cerebral infarction	1.00 (1.00, 1.00)	0.18	1.00 (1.00, 1.00)	0.83	1.00 (1.00, 1.00)	0.78
	Cerebral aneurysm	0.96 (0.86, 1.07)	0.49	0.95 (0.83, 1.09)	0.47	0.85 (0.62, 1.16)	0.33
	Cervical spondylosis	1.00 (1.00, 1.00)	0.34	1.00 (1.00, 1.00)	0.69	1.00 (1.00, 1.00)	0.93
	Spinal canal stenosis	1.03 (0.98, 1.09)	0.25	1.02 (0.94, 1.09)	0.67	1.01 (0.89, 1.14)	0.89
	spinal meningioma	0.72 (0.46, 1.14)	0.16	0.72 (0.39, 1.31)	0.28	0.60 (0.22, 1.68)	0.36
	Spinal osteochondrosis	0.89 (0.60, 1.34)	0.59	0.79 (0.47, 1.32)	0.36	0.49 (0.21, 1.17)	0.15
	Intracranial and intraspinal abscess	0.90 (0.60, 1.35)	0.61	1.01 (0.56, 1.84)	0.97	1.54 (0.61, 3.91)	0.39
	Cervical spinal cord and nerve injuries	1.19 (0.88, 1.62)	0.26	1.18 (0.77, 1.80)	0.45	1.41 (0.70, 2.83)	0.36
	Glioblastoma	1.04 (0.62, 1.75)	0.87	1.27 (0.61, 2.62)	0.52	2.06 (0.64, 6.62)	0.26
	Benign meningioma	0.91 (0.79, 1.05)	0.21	0.92 (0.74, 1.13)	0.41	0.80 (0.58, 1.12)	0.23

	Malignant meningioma	1.01 (0.81, 1.25)	0.93	0.95 (0.72, 1.26)	0.73	0.75 (0.47, 1.20)	0.27
	Pituitary adenoma and craniopharyngioma	0.97 (0.80, 1.18)	0.76	0.96 (0.75, 1.24)	0.78	0.84 (0.53, 1.33)	0.48
	Benign neoplasm of brain and other parts of CNS	1.03 (0.81, 1.30)	0.81	1.02 (0.81, 1.30)	0.85	0.79 (0.47, 1.33)	0.40
	Malignant neoplasm of brain and other parts of CNS	1.58 (1.12, 2.24)	0.01	1.24 (0.77, 2.00)	0.37	0.78 (0.35, 1.71)	0.55
	Hydrocephalus	1.03 (0.86, 1.25)	0.74	1.08 (0.84, 1.39)	0.56	0.93 (0.59, 1.45)	0.75
	Craniosynostosis	1.08 (0.80, 1.46)	0.60	1.18 (0.85, 1.64)	0.32	1.58 (0.82, 3.07)	0.21
	Concussion	1.00 (0.94, 1.06)	0.95	0.95 (0.88, 1.03)	0.22	0.91 (0.80, 1.04)	0.21
	Diffuse brain injury	0.89 (0.73, 1.09)	0.28	0.90 (0.69, 1.19)	0.47	0.77 (0.48, 1.24)	0.31
	Focal brain injury	1.07 (0.92, 1.24)	0.39	1.06 (0.86, 1.30)	0.60	1.06 (0.75, 1.49)	0.75
	Congenital malformations of the nervous system	1.06 (0.75, 1.49)	0.75	1.34 (0..87, 2.07)	0.19	1.53 (0.71, 3.32)	0.31

Outcome	IVW	Weighted Median	MR-Egger

Table S5 Causality of the risk for NOx in Greater Middle Eastern and Neurosurgical multisystem diseases outcomes ($p < 1 \times 10^{-5}$.).

		OR (95% CI)	P	OR (95% CI)	P	OR (95% CI)	P
NOx Greater Middle Eastern	Trigeminal neuralgia	1.07 (0.90, 1.28)	0.43	1.06 (0.86, 1.30)	0.60	1.18 (0.78, 1.79)	0.45
	Epilepsy	0.98 (0.92, 1.05)	0.56	0.97 (0.90, 1.05)	0.51	0.97 (0.82, 1.15)	0.72
	Parkinson's disease	0.97 (0.89, 1.06)	0.50	1.02 (0.92, 1.14)	0.67	1.05 (0.84, 1.30)	0.69
	Alzheimer's disease	1.00 (0.97, 1.03)	0.96	0.99 (0.96, 1.02)	0.65	1.01 (0.94, 1.08)	0.79
	Major depressive disorder	1.03 (0.94, 1.13)	0.51	1.05 (0.93, 1.18)	0.42	0.99 (0.68, 1.43)	0.95
	Obsessive Compulsive Disorder	0.98 (0.88, 1.10)	0.77	0.94 (0.80, 1.11)	0.47	1.03 (0.76, 1.41)	0.84
	Stroke	0.99 (0.96, 1.01)	0.32	0.98 (0.95, 1.02)	0.32	0.99 (0.93, 1.05)	0.71
	Intracerebral hemorrhage	0.99 (0.90, 1.08)	0.76	0.97 (0.86, 1.09)	0.58	1.20 (0.97, 1.48)	0.10
	Subarachnoid hemorrhage	1.01 (0.93, 1.10)	0.76	1.02 (0.90, 1.14)	0.80	1.08 (0.87, 1.34)	0.50
	Transient ischemic attack	1.01 (0.97, 1.06)	0.57	1.01 (0.95, 1.08)	0.75	1.03 (0.93, 1.15)	0.57
	Cerebral infarction	1.00 (1.00, 1.00)	0.90	1.00 (1.00, 1.00)	0.98	1.00 (1.00, 1.00)	0.80
	Cerebral aneurysm	0.98 (0.91, 1.06)	0.59	0.93 (0.84, 1.04)	0.23	1.04 (0.85, 1.27)	0.73
	Cervical spondylosis	1.00 (1.00, 1.00)	0.27	1.00 (1.00, 1.00)	0.28	1.00 (1.00, 1.00)	0.26
	Spinal canal stenosis	0.99 (0.95, 1.03)	0.65	0.99 (0.94, 1.05)	0.86	1.07 (0.97, 1.18)	0.21
	spinal meningioma	0.75 (0.52, 1.08)	0.13	0.70 (0.43, 1.14)	0.15	0.79 (0.34, 1.84)	0.59
	Spinal osteochondrosis	1.10 (0.80, 1.51)	0.57	1.05 (0.68, 1.62)	0.84	0.82 (0.39, 1.73)	0.61
	Intracranial and intraspinal abscess	1.16 (0.74, 1.83)	0.52	0.97 (0.56, 1.69)	0.92	0.61 (0.22, 1.70)	0.36
	Cervical spinal cord and nerve injuries	0.83 (0.65, 1.07)	0.15	0.82 (0.57, 1.19)	0.30	0.82 (0.45, 1.49)	0.53
	Glioblastoma	0.76 (0.50, 1.17)	0.22	0.56 (0.31, 1.02)	0.06	0.83 (0.30, 2.31)	0.72
	Benign meningioma	1.04 (0.89, 1.21)	0.65	0.97 (0.81, 1.16)	0.71	0.98 (0.68, 1.43)	0.93
	Malignant meningioma	1.07 (0.89, 1.29)	0.48	0.99 (0.78, 1.27)	0.96	1.05 (0.67, 1.64)	0.85

	Pituitary adenoma and craniopharyngioma	0.97 (0.83, 1.12)	0.65	1.04 (0.85, 1.28)	0.69	1.05 (0.74, 1.48)	0.79
	Benign neoplasm of brain and other parts of CNS	0.93 (0.82, 1.06)	0.30	1.01 (0.84, 1.22)	0.92	1.19 (0.88, 1.62)	0.27
	Malignant neoplasm of brain and other parts of CNS	1.10 (0.83, 1.46)	0.50	1.10 (0.73, 1.66)	0.65	1.10 (0.57, 2.11)	0.79
	Hydrocephalus	0.98 (0.85, 1.14)	0.82	0.92 (0.74, 1.15)	0.46	0.81 (0.58, 1.14)	0.25
	Craniosynostosis	0.93 (0.72, 1.20)	0.58	0.99 (0.73, 1.35)	0.95	1.16 (0.63, 2.12)	0.64
	Concussion	1.03 (0.99, 1.07)	0.20	1.02 (0.96, 1.08)	0.55	0.99 (0.90, 1.10)	0.92
	Diffuse brain injury	0.87 (0.75, 1.02)	0.09	0.90 (0.72, 1.12)	0.34	1.03 (0.72, 1.48)	0.87
	Focal brain injury	0.92 (0.80, 1.04)	0.18	0.89 (0.74, 1.07)	0.22	0.88 (0.65, 1.20)	0.43
	Congenital malformations of the nervous system	0.85 (0.66, 1.09)	0.19	0.86 (0.61, 1.23)	0.41	0.59 (0.33, 1.05)	0.10

Outcome		IVW		Weighted Median		MR-Egger	
		OR (95% CI)	P	OR (95% CI)	P	OR (95% CI)	P

Table S6 Causality of the risk for NO_2 in European and Neurosurgical multisystem diseases outcomes ($p < 1 \times 10^{-5}$.).

NO_2 European	Trigeminal neuralgia	4.53 (0.05, 442.66)	0.52	11.38 (0.05, 2.47e+3)	0.38	0.00 (1.98e-12, 2.14e+5)	0.54
	Epilepsy	0.40 (0.06, 2.64)	0.34	0.72 (0.12, 4.50)	0.73	38.04 (0.05, 3.12e+4)	0.40
	Parkinson's disease	5.19 (0.13, 2.13e+2)	0.39	4.29 (0.19, 95,12)	0.36	1.26e+4 (0.00, 2.87e+11)	0.35
	Alzheimer's disease	1.30 (0.54, 3.14)	0.56	1.53 (0.70, 3.37)	0.29	0.18 (0.00, 12.60)	0.51
	Major depressive disorder	0.01 (4.29e-3, 2.72)	0.18	0.08 (4.43e-3, 1.31)	0.08	1.93e-9 (3.98e-15, 9.35e-4)	0.10
	Obsessive Compulsive Disorder	3.78 (0.26, 55.10)	0.33	2.76 (0.13, 58.01)	0.51	5.63 (1.12e-5, 2.83e+6)	0.82
	Stroke	1.59 (0.82, 3.07)	0.17	1.32 (0.59, 2.92)	0.50	7.07 (0.44, 112.54)	0.30
	Intracerebral hemorrhage	0.88 (0.03, 26.49)	0.94	0.22 (0.02, 3.11)	0.26	8.05e-5 (3.10e-9, 2.09)	0.21
	Subarachnoid hemorrhage	1.28 (0.06, 27.28)	0.87	0.46 (0.03, 7.27)	0.58	0.16 (5.63e-8, 4.80e+5)	0.83
	Transient ischemic attack	1.82 (0.39, 8.55)	0.45	2.09 (0.37, 11.83)	0.41	202.69 (0.36, 1.13e+5)	0.24
	Cerebral infarction	1.03 (1.01, 1.06)	0.01	1.02 (1.00, 1.04)	0.04	1.12 (1.04, 1.21)	0.09
	Cerebral aneurysm	0.55 (0.04, 7.34)	0.65	0.33 (0.04, 2.74)	0.31	0.10 (8.91e-7, 1.16e+4)	0.74
	Cervical spondylosis	0.99 (0.98, 1.01)	0.24	0.99 (0.98, 1.01)	0.28	0.99 (0.93, 1.06)	0.83
	Spinal canal stenosis	0.36 (0.11, 1.24)	0.11	0.37 (0.09, 1.41)	0.14	0.62 (0.00, 155.45)	0.88
	spinal meningioma	0.04 (3.24e-7, 5.80e+3)	0.60	6.02e-3 (6.99e-9, 5.19e+3)	0.46	1.33e+8 (1.87e-14, 9.46e+29)	0.54
	Spinal osteochondrosis	0.03 (4.71e-5, 1.90e+3)	0.53	0.16 (2.25e-6, 1.18e+4)	0.75	3.52e+8 (7.42e-13, 1.67e+29)	0.52
	Intracranial and intraspinal abscess	1.43 (2.27e-7, 9.04e+6)	0.96	69.49 (8.00e-5, 6.04e+7)	0.54	5.32e+9 (1.06e-23, 2.68e+42)	0.62
	Cervical spinal cord and nerve injuries	30.53 (9.39e-3, 9.93e+4)	0.41	80.89 (8.49e-3, 7.71e+5)	0.35	8.70e-3 (6.26e-18. 1.21e+13)	0.81
	Glioblastoma	0.72 (1.25e-6, 4.10e+5)	0.96	0.94 (1.16e-7, 7.60e+6)	0.99	2.24e-14 (4.44e-39, 1.13e+11)	0.39

	Benign meningioma	0.12 (2.46e-3, 5.39)	0.27	0.03 (3.04e-4, 2.56)	0.12	0.01 (1.87e-11, 9.02e+5)	0.63
	Malignant meningioma	0.03 (2.39e-6, 3.30e+2)	0.45	1.97e-3 (2.71e-6, 1.43)	0.06	1.07e-9 (3.60e-28, 3.15e+9)	0.44
	Pituitary adenoma and craniopharyngioma	11.99 (7.78e-3, 1.85e+4)	0.51	21.85 (0.05, 9.44e+3)	0.32	3.71e+7 (6.30e-7, 2.18e+21)	0.39
	Benign neoplasm of brain and other parts of CNS	44.05 (0.50, 3.85e+3)	0.10	50.11 (0.27, 9.18e+3)	0.14	0.01 (1.84e-11, 2.44e+5)	0.58
	Malignant neoplasm of brain and other parts of CNS	82.23 (9.27e-3, 7.29e+5)	0.34	53.42 (9.47e-4, 3.01e+6)	0.48	3.47e+10 (5.17e-7, 2.33e+27)	0.34
	Hydrocephalus	5.45 (0.05, 6.41e+2)	0.49	19.94 (0.09, 4.52e+3)	0.28	1.72 (2.12e-9, 1.39e+9)	0.96
	Craniosynostosis	6.39e+2 (1.01, 4.06e+5)	0.04	2.44e+2 (0.13, 4.75e+5)	0.15	0.02 (2.54e-14, 1.78e+10)	0.81
	Concussion	0.20 (0.05, 0.77)	0.02	0.20 (0.04, 0.89)	0.04	0.07 (2.10e-4, 22.41)	0.46
	Diffuse brain injury	1.28e+2 (0.82, 2.02e+4)	0.06	70.27 (0.10, 4.72e+4)	0.20	0.13 (1.04e-11, 1.64e+9)	0.88
	Focal brain injury	13.27 (3.01e-3, 5.86e+4)	0.55	78.26 (0.35, 1.76e+4)	0.11	1.20e-3 (5.04e-22, 2.87e+15)	0.78
	Congenital malformations of the nervous system	4.93e-4 (1.42e-7, 1.70)	0.07	9.12e-5 (6.15e-9, 1.35)	0.06	5.49e-4 (4.11e-19, 7..33e+11)	0.71

Outcome		IVW		Weighted Median		MR-Egger	
		OR (95% CI)	P	OR (95% CI)	P	OR (95% CI)	P
NO_2	Trigeminal neuralgia	0.68 (0.54,	0.002	0.79 (0.56,	0.18	0.68 (0.41,	0.15

Table S7 Causality of the risk for NO_2 in African American or Afro-Caribbean and Neurosurgical multisystem diseases outcomes ($p < 1 \times 10^{-5}$.).

African American or Afro-Caribbean		0.87)		1.12)		1.13)	
	Epilepsy	1.00 (0.92, 1.08)	0.99	0.99 (0.88, 1.11)	0.81	0.99 (0.84, 1.17)	0.92
	Parkinson's disease	0.96 (0.85, 1.08)	0.51	0.93 (0.77, 1.12)	0.43	0.83 (0.64, 1.08)	0.17
	Alzheimer's disease	1.01 (0.98, 1.05)	0.48	1.00 (0.94, 1.05)	0.91	0.98 (0.90, 1.06)	0.63
	Major depressive disorder	1.06 (0.93, 1.22)	0.37	1.01 (0.83, 1.22)	0.95	0.92 (0.63, 1.34)	0.66
	Obsessive Compulsive Disorder	1.06 (0.89. 1.26)	0.50	1.02 (0.80, 1.30)	0.88	0.89 (0.62, 1.27)	0.52
	Stroke	1.02 (0.98, 1.07)	0.26	1.02 (0.96, 1.07)	0.57	1.02 (0.93, 1.12)	0.69
	Intracerebral hemorrhage	1.04 (0.93, 1.17)	0.46	1.04 (0.87, 1.23)	0.67	0.98 (0.78, 1.24)	0.86
	Subarachnoid hemorrhage	0.91 (0.80, 1.04)	0.16	0.87 (0.72, 1.05)	0.14	0.88 (0.67, 1.17)	0.39
	Transient ischemic attack	0.91 (0.84, 0.98)	0.01	0.92 (0.81, 1.03)	0.15	0.95 (0.81, 1.12)	0.56
	Cerebral infarction	1.00 (1.00, 1.00)	0.11	1.00 (1.00, 1.00)	0.55	1.00 (1.00, 1.00)	0.40
	Cerebral aneurysm	0.94 (0.84, 1.04)	0.25	0.94 (0.80, 1.11)	0.49	0.84 (0.68, 1.04)	0.12
	Cervical spondylosis	1.00 (1.00, 1.00)	0.59	1.00 (1.00, 1.00)	0.91	1.00 (1.00, 1.00)	0.82
	Spinal canal stenosis	1.02 (0.95, 1.09)	0.52	0.99 (0.89, 1.10)	0.84	1.01 (0.87, 1.17)	0.89
	spinal meningioma	0.76 (0.41, 1.43)	0.40	0.82 (0.34, 2.02)	0.67	0.65 (0.17, 2.48)	0.53
	Spinal osteochondrosis	1.31 (0.80, 2.16)	0.28	1.19 (0.59, 2.41)	0.63	0.71 (0.25, 2.03)	0.53
	Intracranial and intraspinal abscess	1.34 (0.76, 2.37)	0.30	1.65 (0.73, 3.74)	0.23	1.37 (0.42, 4.49)	0.61
	Cervical spinal cord and nerve injuries	1.05 (0.69, 1.61)	0.81	1.24 (0.67, 2.28)	0.49	1.17 (0.48, 2.85)	0.73
	Glioblastoma	0.83 (0.41, 1.68)	0.60	1.11 (0.35, 3.55)	0.86	1.51 (0.34, 6.74)	0.59
	Benign meningioma	1.04 (0.85, 1.27)	0.70	1.12 (0.85, 1.48)	0.43	1.19 (0.78, 1.82)	0.42
	Malignant meningioma	1.15 (0.88, 1.50)	0.31	1.25 (0.85, 1.83)	0.26	1.27 (0.72, 2.23)	0.42
	Pituitary adenoma and craniopharyngioma	0.98 (0.77, 1.26)	0.90	1.16 (0.81, 1.66)	0.41	1.18 (0.69, 1.99)	0.56

	Benign neoplasm of brain and other parts of CNS	1.02 (0.80, 1.29)	0.87	1.09 (0.80, 1.50)	0.58	1.47 (0.90, 2.39)	0.13
	Malignant neoplasm of brain and other parts of CNS	1.18 (0.73, 1.90)	0.50	0.97 (0.47, 1.99)	0.94	0.92 (0.34, 2.53)	0.88
	Hydrocephalus	0.84 (0.66, 1.07)	0.17	0.77 (0.53, 1.13)	0.18	0.13 (0.67, 1.89)	0.66
	Craniosynostosis	0.84 (0.59, 1.19)	0.33	0.87 (0.52, 1.46)	0.60	0.68 (0.32, 1.43)	0.32
	Concussion	0.99 (0.93, 1.06)	0.83	1.02 (0.91, 1.14)	0.74	1.11 (0.95, 1.28)	0.19
	Diffuse brain injury	0.98 (0.75, 1.27)	0.86	0.81 (0.55, 1.20)	0.29	0.82 (0.47, 1.42)	0.48
	Focal brain injury	0.91 (0.74, 1.12)	0.39	0.93 (0.68, 1.27)	0.64	1.02 (0.66, 1.58)	0.93
	Congenital malformations of the nervous system	0.97 (0.62, 1.50)	0.87	0.82 (0.44, 1.53)	0.53	0.52 (0.21, 1.27)	0.16

Outcome		IVW		Weighted Median		MR-Egger	
		OR (95% CI)	P	OR (95% CI)	P	OR (95% CI)	P
NO_2 South Asian	Trigeminal neuralgia	1.12 (0.77, 1.62)	0.55	1.01 (0.59, 1.70)	0.98	1.58 (0.58, 4.31)	0.38

Table S8 Causality of the risk for NO_2 in South Asian and Neurosurgical multisystem diseases outcomes (p < 1 × 10-5.).

	Epilepsy	0.90 (0.79, 1.03)	0.13	0.87 (0.72, 1.06)	0.17	0.96 (0.65, 1.41)	0.84
	Parkinson's disease	1.06 (0.87, 1.30)	0.56	1.00 (0.75, 1.33)	0.99	1.67 (0.99, 2.85)	0.07
	Alzheimer's disease	0.98 (0.91, 1.04)	0.49	0.99 (0.91, 1.07)	0.81	1.10 (0.92, 1.31)	0.32
	Major depressive disorder	1.01 (0.81, 1.27)	0.91	0.95 (0.71, 1.28)	0.76	0.77 (0.32, 1.90)	0.58
	Obsessive Compulsive Disorder	1.02 (0.77, 1.35)	0.91	1.10 (0.75, 1.64)	0.62	0.76 (0.35, 1.65)	0.50
	Stroke	1.08 (1.01, 1.15)	0.02	1.05 (0.96, 1.15)	0.28	1.03 (0.86, 1.23)	0.76
	Intracerebral hemorrhage	0.93 (0.76, 1.14)	0.48	0.86 (0.65, 1.14)	0.29	0.97 (0.55, 1.72)	0.93
	Subarachnoid hemorrhage	1.12 (0.91, 1.38)	0.30	1.28 (0.95, 1.73)	0.11	1.31 (0.72, 2.37)	0.38
	Transient ischemic attack	1.01 (0.89, 1.15)	0.86	0.99 (0.84, 1.16)	0.88	0.77 (0.56, 1.06)	0.13
	Cerebral infarction	1.00 (1.00, 1.00)	0.09	1.00 (1.00, 1.00)	0.34	1.00 (1.00, 1.00)	0.95
	Cerebral aneurysm	1.02 (0.84, 1.25)	0.82	0.96 (0.73, 1.26)	0.75	1.05 (0.56, 1.96)	0.89
	Cervical spondylosis	1.00 (1.00, 1.00)	0.26	1.00 (1.00, 1.00)	0.63	1.00 (1.00, 1.00)	0.71
	Spinal canal stenosis	1.03 (0.91, 1.17)	0.68	1.04 (0.89, 1.22)	0.63	0.70 (0.52, 0.94)	0.03
	spinal meningioma	0.28 (0.11, 0.75)	0.01	0.20 (0.06, 0.70)	0.01	0.23 (0.02, 3.13)	0.29
	Spinal osteochondrosis	1.03 (0.46, 2.29)	0.95	0.42 (0.13, 1.33)	0.14	1.03 (0.11, 9.53)	0.98
	Intracranial and intraspinal abscess	0.87 (0.34, 2.22)	0.77	0.57 (0.15, 2.13)	0.40	2.34 (0.18, 30.35)	0.52
	Cervical spinal cord and nerve injuries	0.97 (0.49, 1.91)	0.94	0.63 (0.25, 1.56)	0.32	0.36 (0.06, 2.19)	0.28
	Glioblastoma	0.54 (0.18, 1.62)	0.27	0.44 (0.10, 2.02)	0.29	0.27 (0.01, 5.11)	0.39
	Benign meningioma	0.88 (0.65, 1.21)	0.43	0.76 (0.50, 1.15)	0.19	0.77 (0.33, 1.79)	0.55
	Malignant meningioma	0.86 (0.57, 1.30)	0.48	0.71 (0.40, 1.27)	0.25	0.91 (0.30, 2.79)	0.87
	Pituitary adenoma and craniopharyngioma	1.06 (0.72, 1.57)	0.75	0.83 (0.48, 1.42)	0.49	0.93 (0.33, 2.64)	0.90
	Benign neoplasm of brain and other parts	0.72 (0.51, 1.02)	0.07	0.79 (0.49, 1.28)	0.34	0.93 (0.36, 2.41)	0.88

	of CNS						
	Malignant neoplasm of brain and other parts of CNS	1.01 (0.45, 2.26)	0.97	1.39 (0.48, 4.05)	0.54	1.19 (0.13, 11.02)	0.88
	Hydrocephalus	0.80 (0.53, 1.21)	0.29	1.02 (0.56, 1.87)	0.94	1.62 (0.54, 4.85)	0.40
	Craniosynostosis	0.70 (0.42, 1.18)	0.18	0.90 (0.41, 1.98)	0..79	2.23 (0.54, 9.12)	0.28
	Concussion	0.99 (0.89, 1.10)	0.85	1.04 (0.89, 1.23)	0.59	1.22 (0.91, 1.64)	0.20
	Diffuse brain injury	1.15 (0.70, 1.91)	0.58	1.13 (0.61, 2.09)	0.69	0.75 (0.19, 3.00)	0.69
	Focal brain injury	0.71 (0.49, 1.01)	0.06	0.77 (0.47, 1.25)	0.29	0.45 (0.17, 1.19)	0.12
	Congenital malformations of the nervous system	1.42 (0.72, 2.81)	0.31	1.06 (0.40, 2.77)	0.91	3.00 (0.47, 19.34)	0.26

Outcome		IVW		Weighted Median		MR-Egger	
		OR (95% CI)	P	OR (95% CI)	P	OR (95% CI)	P
NO_2 East Asian	Trigeminal neuralgia	1.21 (1.02, 1.43)	0.03	1.15 (0.91, 1.46)	0.24	1.19 (0.84, 1.69)	0.35
	Epilepsy	0.10 (0.97, 1.10)	0.27	1.03 (0.95, 1.12)	0.44	1.01 (0.89. 1.14)	0.88

Table S9 Causality of the risk for NO_2 in East Asian and Neurosurgical multisystem diseases outcomes (p < 1 × 10-5.).

	Parkinson's disease	0.98 (0.90, 1.06)	0.57	1.05 (0.94, 1.17)	0.39	0.94 (0.81, 1.10)	0.47
	Alzheimer's disease	0.98 (0.96, 1.01)	0.25	1.00 (0.96, 1.04)	0.89	1.01 (0.96, 1.06)	0.80
	Major depressive disorder	0.97 (0.92, 1.04)	0.42	0.95 (0.87, 1.04)	0.26	0.90 (0.78, 1.05)	0.22
	Obsessive Compulsive Disorder	1.01 (0.91, 1.12)	0.84	0.97 (0.86, 1.11)	0.67	0.93 (0.75, 1.15)	0.52
	Stroke	0.98 (0.95, 1.00)	0.04	0.97 (0.94, 1.01)	0.12	0.97 (0.92, 1.02)	0.27
	Intracerebral hemorrhage	0.95 (0.87, 1.05)	0.31	0.95 (0.84, 1.08)	0.45	1.03 (0.85, 1.24)	0.77
	Subarachnoid hemorrhage	1.11 (1.01, 1.21)	0.02	1.13 (1.00, 1.27)	0.05	1.10 (0.92, 1.32)	0.31
	Transient ischemic attack	1.05 (0.99, 1.11)	0.08	1.04 (0.96, 1.12)	0.36	1.02 (0.91, 1.14)	0.77
	Cerebral infarction	1.00 (1.00, 1.00)	0.17	1.00 (1.00, 1.00)	0.58	1.00 (1.00, 1.00)	0.38
	Cerebral aneurysm	0.98 (0.88, 1.08)	0.65	0.95 (0.84, 1.09)	0.48	0.98 (0.76, 1.27)	0.89
	Cervical spondylosis	1.00 (1.00, 1.00)	0.70	1.00 (1.00, 1.00)	0.80	1.00 (1.00, 1.00)	0.19
	Spinal canal stenosis	0.98 (0.94, 1.02)	0.36	0.97 (0.92, 1.03)	0.38	0.95 (0.88, 1.03)	0.25
	spinal meningioma	0.99 (0.59, 1.65)	0.96	0.81 (0.45, 1.47)	0.49	0.81 (0.27, 2.40)	0.71
	Spinal osteochondrosis	1.29 (0.91, 1.85)	0.16	1.34 (0.82, 2.18)	0.25	1.40 (0.68, 2.87)	0.39
	Intracranial and intraspinal abscess	0.97 (0.63, 1.51)	0.90	0.81 (0.45, 1.47)	0.49	0.86 (0.34, 2.18)	0.75
	Cervical spinal cord and nerve injuries	0.82 (0.61, 1.12)	0.21	0.86 (0.57, 1.30)	0.48	0.66 (0.36, 1.22)	0.22
	Glioblastoma	0.99 (0.60, 1.64)	0.96	1.01 (0.49, 2.07)	0.98	0.92 (0.33, 2.54)	0.87
	Benign meningioma	1.07 (0.93, 1.24)	0.34	1.03 (0.83, 1.27)	0.78	1.26 (0.94, 1.69)	0.16
	Malignant meningioma	1.05 (0.83, 1.32)	0.71	0.95 (0.71, 1.27)	0.73	1.48 (0.97, 2.25)	0.10
	Pituitary adenoma and craniopharyngioma	1.24 (1.02, 1.52)	0.03	1.25 (0.96, 1.63)	0.10	1.12 (0.74, 1.71)	0.60
	Benign neoplasm of brain and other parts of CNS	1.09 (0.93, 1.29)	0.27	1.05 (0.85, 1.31)	0.63	1.03 (0.74, 1.42)	0.88
	Malignant neoplasm of	1.13 (0.71,	0.60	0.86 (0.53,	0.33	0.90 (0.34,	0.83

	brain and other parts of CNS	1.79)		1.40)		2.37)	
	Hydrocephalus	1.13 (0.94, 1.35)	0.19	1.17 (0.93, 1.47)	0.19	1.10 (0.76, 1.57)	0.63
	Craniosynostosis	0.74 (0.56, 0.98)	0.04	0.74 (0.53, 1.04)	0.08	0.58 (0.33, 1.01)	0.09
	Concussion	1.00 (0.95, 1.05)	0.92	0.99 (0.93, 1.06)	0.85	0.95 (0.86, 1.06)	0.40
	Diffuse brain injury	0.87 (0.72, 1.05)	0.15	0.85 (0.65, 1.10)	0.21	0.85 (0.58, 1.25)	0.44
	Focal brain injury	1.17 (1.01, 1.36)	0.04	1.10 (0.89, 1.37)	0.36	1.11 (0.82, 1.50)	0.51
	Congenital malformations of the nervous system	1.00 (0.74, 1.36)	0.99	1.10 (0.72, 1.69)	0.65	1.09 (0.57, 2.11)	0.80

Outcome		IVW		Weighted Median		MR-Egger	
		OR (95% CI)	P	OR (95% CI)	P	OR (95% CI)	P
NO_2 Greater Middle Eastern and Ne	Trigeminal neuralgia	0.97 (0.85, 1.11)	0.68	1.03 (0.86, 1.24)	0.75	1.08 (0.81, 1.45)	0.62
	Epilepsy	1.02 (0.97, 1.07)	0.40	0.99 (0.93, 1.06)	0.84	1.05 (0.94, 1.18)	0.39
	Parkinson's disease	1.03 (0.96, 1.09)	0.44	1.08 (0.99, 1.17)	0.10	1.12 (0.97, 1.29)	0.13

Table S10 Causality of the risk for NO_2 in Greater Middle Eastern and Neurosurgical multisystem diseases outcomes (p < 1 × 10-5.).

	Alzheimer's disease	0.99 (0.97, 1.01)	0.30	0.98 (0.96, 1.01)	0.25	1.03 (0.98, 1.07)	0.24
	Major depressive disorder	1.02 (0.94, 1.10)	0.64	1.02 (0.92, 1.14)	0.71	0.97 (0.72, 1.29)	0.82
	Obsessive Compulsive Disorder	1.01 (0.92, 1.11)	0.79	0.97 (0.86, 1.10)	0.64	0.94 (0.76, 1.16)	0.58
	Stroke	1.01 (0.99, 1.03)	0.33	1.01 (0.99, 1.05)	0.33	1.04 (0.98, 1.10)	0.27
	Intracerebral hemorrhage	1.05 (0.98, 1.12)	0.16	1.02 (0.93, 1.13)	0.62	1.24 (1.06, 1.45)	0.01
	Subarachnoid hemorrhage	1.00 (0.92, 1.08)	0.95	1.04 (0.93, 1.15)	0.50	0.95 (0.79, 1.15)	0.63
	Transient ischemic attack	0.99 (0.95, 1.03)	0.72	0.99 (0.94, 1.05)	0.82	1.03 (0.95. 1.13)	0.47
	Cerebral infarction	1.00 (1.00, 1.00)	0.06	1.00 (1.00, 1.00)	0.80	1.00 (1.00, 1.00)	0.10
	Cerebral aneurysm	1.01 (0.94, 1.08)	0.87	0.98 (0.89, 1.08)	0.63	1.22 (1.01, 1.48)	0.06
	Cervical spondylosis	1.00 (1.00, 1.00)	0.46	1.00 (1.00, 1.00)	0.46	1.00 (1.00, 1.00)	0.25
	Spinal canal stenosis	1.07 (1.03, 1.11)	0.0003	1.06 (1.00, 1.12)	0.03	1.10 (1.01, 1.19)	0.03
	spinal meningioma	1.15 (0.83, 1.59)	0.40	1.03 (0.66, 1.60)	0.90	0.97 (0.48, 1.96)	0.93
	Spinal osteochondrosis	1.09 (0.84, 1.42)	0.50	1.21 (0.84, 1.74)	0.31	1.22 (0.69, 2.15)	0.51
	Intracranial and intraspinal abscess	1.14 (0.84, 1.55)	0.39	1.45 (0.92, 2.29)	0.11	1.10 (0.56, 2.18)	0.78
	Cervical spinal cord and nerve injuries	0.81 (0.61, 1.08)	0.15	0.82 (0.60, 1.13)	0.22	0.90 (0.47, 1.71)	0.75
	Glioblastoma	1.06 (0.73, 1.53)	0.77	0.90 (0.54, 1.49)	0.68	1.20 (0.54, 2.66)	0.67
	Benign meningioma	1.03 (0.91, 1.16)	0.67	0.95 (0.82, 1.10)	0.48	1.08 (0.82, 1.43)	0.59
	Malignant meningioma	0.98 (0.85, 1.13)	0.78	0.98 (0.81, 1.19)	0.86	1.25 (0.92, 1.69)	0.17
	Pituitary adenoma and craniopharyngioma	1.07 (0.92, 1.25)	0.37	1.22 (1.00, 1.48)	0.05	1.39 (1.01, 1.91)	0.06
	Benign neoplasm of brain and other parts of CNS	1.03 (0.92, 1.16)	0.59	1.04 (0.88, 1.22)	0.67	0.94 (0.73, 1.21)	0.65
	Malignant neoplasm of brain and other parts of CNS	0.92 (0.70, 1.23)	0.59	0.85 (0.58, 1.24)	0.39	0.51 (0.29, 0.89)	0.03

	Hydrocephalus	1.07 (0.94, 1.23)	0.30	1.10 (0.92, 1.33)	0.30	0.97 (0.72, 1.31)	0.86
	Craniosynostosis	1.14 (0.96, 1.36)	0.14	1.20 (0.94, 1.54)	0.14	1.19 (0.82, 1.75)	0.37
	Concussion	1.00 (0.97, 1.04)	0.95	0.98 (0.93, 1.03)	0.39	1.03 (0.95, 1.12)	0.43
	Diffuse brain injury	1.00 (0.87, 1.14)	0.95	0.92 (0.76, 1.12)	0.42	1.38 (1.02, 1.86)	0.05
	Focal brain injury	0.92 (0.82, 1.02)	0.11	0.91 (0.78, 1.07)	0.25	0.95 (0.75, 1.21)	0.70
	Congenital malformations of the nervous system	1.06 (0.84, 1.33)	0.62	0.89 (0.64, 1.23)	0.47	0.78 (0.48, 1.26)	0.33

Outcome		IVW		Weighted Median		MR-Egger	
		OR (95% CI)	P	OR (95% CI)	P	OR (95% CI)	P
PM2.5 European	Trigeminal neuralgia	0.78 (0.15, 4.13)	0.77	0.77 (0.13, 4.39)	0.77	0.72 (0.07, 7.79)	0.80
	Epilepsy	0.61 (0.28, 1.32)	0.21	0.62 (0.38, 1.02)	0.06	0.63 (0.21, 1.93)	0.45
	Parkinson's disease	1.40 (0.57, 3.45)	0.46	1.44 (0.51, 4.10)	0.49	1.75 (0.53, 5.80)	0.41
	Alzheimer's disease	0.99 (0.61, 1.61)	0.96	0.77 (0.41, 1.42)	0.40	0.87 (0.35, 2.18)	0.78

Table S11 Causality of the risk for PM2.5 in European and Neurosurgical multisystem diseases outcomes ($p < 1 \times 10-5.$).

	Major depressive disorder	0.26 (2.6e-3, 25.58)	0.56	0.02 (1.01e-3, 0.22)	0.002	1.99e-3 (1.14e-15, 3.47e+9)	0.69
	Obsessive Compulsive Disorder	0.56 (0.09, 3.65)	0.55	0.62 (0.06, 6.22)	0.69	0.63 (0.04, 9.92)	0.77
	Stroke	0.99 (0.69, 1.43)	0.97	0.94 (0.68, 1.28)	0.68	0.83 (0.53, 1.31)	0.47
	Intracerebral hemorrhage	1.13 (0.62, 2.06)	0.68	1.11 (0.57, 2.14)	0.77	1.07 (0.49, 2.33)	0.87
	Subarachnoid hemorrhage	1.03 (0.41, 2.63)	0.94	1.05 (0.49, 2.21)	0.91	1.20 (0.32, 4.55)	0.80
	Transient ischemic attack	1.13 (0.64, 1.98)	0.67	1.08 (0.62, 1.89)	0.78	0.97 (0.45, 2.10)	0.94
	Cerebral infarction	1.02 (1.00, 1.03)	0.02	1.02 (1.00, 1.04)	0.01	1.00 (0.95, 1.05)	0.90
	Cerebral aneurysm	0.82 (0.50, 1.34)	0.43	0.81 (0.50, 1.31)	0.38	0.71 (0.35, 1.45)	0.40
	Cervical spondylosis	1.00 (0.99, 1.02)	0.65	1.00 (0.99, 1.02)	0.50	1.03 (0.98, 1.08)	0.27
	Spinal canal stenosis	0.92 (0.41, 2.03)	0.83	0.82 (0.45, 1.49)	0.52	0.64 (0.23, 1.79)	0.44
	spinal meningioma	0.37 (2.56e-3, 54.41)	0.70	0.42 (2.74e-3, 63.25)	0.73	0.49 (3.76e-4, 629.11)	0.85
	Spinal osteochondrosis	0.56 (0.02, 17.81)	0.75	0.32 (0.01, 15.61)	0.56	0.21 (0.00, 18.22)	0.53
	Intracranial and intraspinal abscess	0.38 (3.86e-3, 48.00)	0.70	0.11 (1.45e-3, 8.62)	0.32	0.02 (1.10e-4, 4.18)	0.23
	Cervical spinal cord and nerve injuries	0.20 (0.01, 3.69)	0.28	0.13 (5.29e-3, 3.03)	0.20	0.06 (1.50e-3, 2.73)	0.22
	Glioblastoma	1.47 (0.01, 200.74)	0.88	1.07 (0.01, 227.57)	0.98	1.01 (0.00, 567.77)	0.99
	Benign meningioma	1.48 (0.37, 5.92)	0.58	1.73 (0.38, 7.85)	0.48	1.81 (0.30, 10.75)	0.55
	Malignant meningioma	1.83 (0.24, 14.13)	0.56	1.15 (0.15, 8.90)	0.90	0.75 (0.06, 9,96)	0.84
	Pituitary adenoma and craniopharyngioma	0.36 (0.04, 3.50)	0.38	0.69 (0.10, 4.63)	0.70	1.16 (0.08, 17.53)	0.92
	Benign neoplasm of brain and other parts of CNS	2.21 (0.25, 19.43)	0.47	3.08 (0.51, 18.43)	0.22	8.51 (0.84, 86.50)	0.14
	Malignant neoplasm of	2.64 (0.09,	0.57	6.62 (0.16,	0.32	21.45 (0.29,	0.23

	brain and other parts of CNS	73.90)		280.71)		1.57e+3)	
	Hydrocephalus	1.55 (0.29, 8.38)	0.61	1.12 (0.16, 7.73)	0.91	0.53 (0.06, 4.64)	0.60
	Craniosynostosis	0.48 (0.05, 4.66)	0.52	0.45 (0.04, 5.33)	0.52	0.20 (0.01. 3.85)	0.35
	Concussion	0.78 (0.47, 1.30)	0.35	0.81 (0.48, 1.35)	0.42	0.81 (0.39, 1.68)	0.59
	Diffuse brain injury	2.32 (0.13, 41.75)	0.57	3.51 (0.45, 27.34)	0.23	3.46 (0.06, 209.60)	0.59
	Focal brain injury	0.64 (0.16, 2.65)	0.54	0.53 (0.10, 2.75)	0.45	0.45 (0.07, 2.81)	0.44
	Congenital malformations of the nervous system	0.85 (0.05, 14.91)	0.91	0.92 (0.04, 19.78)	0.96	0.90 (0.02, 35.63)	0.96

Outcome		IVW		Weighted Median		MR-Egger	
		OR (95% CI)	P	OR (95% CI)	P	OR (95% CI)	P
PM2.5 African American or Afro-Caribbean	Trigeminal neuralgia	0.85 (0.62, 1.16)	0.31	0.89 (0.53, 1.47)	0.64	1.11 (0.47, 2.60)	0.81
	Epilepsy	0.89 (0.79, 1.00)	0.048	0.85 (0.72, 1.02)	0.08	0.95 (0.70, 1.30)	0.76
	Parkinson's disease	1.05 (0.89, 1.25)	0.55	1.01 (0.76, 1.33)	0.96	0.88 (0.57, 1.36)	0.57
	Alzheimer's disease	1.02 (0.97, 1.07)	0.47	1.01 (0.94, 1.09)	0.72	0.96 (0.86, 1.08)	0.55
	Major depressive	0.91 (0.78,	0.22	0.87 (0.71,	0.17	0.83 (0.51,	0.45

Table S12 Causality of the risk for PM2.5 in African American or Afro-Caribbean and Neurosurgical multisystem diseases outcomes (p < 1 × 10-5.).

	disorder	1.06)		1.06)		1.33)	
	Obsessive Compulsive Disorder	0.99 (0.78, 1.26)	0.97	1.02 (0.72, 1.44)	0.91	1.16 (0.64, 2.08)	0.63
	Stroke	1.03 (0.98, 1.09)	0.23	1.02 (0.95, 1.11)	0.57	1.01 (0.87, 1.17)	0.89
	Intracerebral hemorrhage	1.04 (0.88, 1.22)	0.68	1.02 (0.79, 1.31)	0.90	0.96 (0.62, 1.49)	0.87
	Subarachnoid hemorrhage	0.75 (0.61, 0.91)	0.003	0.75 (0.57, 0.98)	0.04	0.80 (0.47, 1.35)	0.41
	Transient ischemic attack	0.97 (0.88, 1.08)	0.58	1.04 (0.90, 1.21)	0.60	1.04 (0.79, 1.37)	0.79
	Cerebral infarction	1.00 (1.00, 1.00)	0.11	1.00 (1.00, 1.00)	0.20	1.00 (1.00, 1.00)	0.57
	Cerebral aneurysm	0.98 (0.83, 1.15)	0.78	0.96 (0.75, 1.22)	0.75	1.09 (0.68, 1.72)	0.73
	Cervical spondylosis	1.00 (1.00, 1.00)	0.43	1.00 (1.00, 1.00)	0.66	1.00 (1.00, 1.00)	0.96
	Spinal canal stenosis	1.00 (0.92, 1.10)	0.92	1.00 (0.87, 1.15)	0.99	1.05 (0.83, 1.33)	0.68
	spinal meningioma	0.67 (0.30, 1.54)	0.35	1.12 (0.32, 3.95)	0.87	2.80 (0.31, 25.19)	0.37
	Spinal osteochondrosis	0.88 (0.45, 1.71)	0.70	0.71 (0.25, 2.06)	0.53	0.68 (0.12, 4.02)	0.68
	Intracranial and intraspinal abscess	0.64 (0.30, 1.36)	0.24	0.48 (0.16, 1.47)	0.20	0.41 (0.05, 3.09)	0.40
	Cervical spinal cord and nerve injuries	0.74 (0.41, 1.37)	0.34	0.58 (0.26, 1.28)	0.18	0.46 (0.09, 2.37)	0.36
	Glioblastoma	0.80 (0.31, 2.05)	0.64	1.00 (0.27, 3.76)	0.99	0.19 (0.02, 2.30)	0.20
	Benign meningioma	0.95 (0.71, 1.27)	0.74	0.90 (0.59, 1.37)	0.61	0.98 (0.44, 2.19)	0.97
	Malignant meningioma	1.02 (0.72, 1.46)	0.90	0.97 (0.55, 1.69)	0.90	1.33 (0.51, 3.45)	0.57
	Pituitary adenoma and craniopharyngioma	1.16 (0.83, 1.62)	0.38	0.97 (0.59, 1.60)	0.91	0.82 (0.34, 2.01)	0.68
	Benign neoplasm of brain and other parts of CNS	0.91 (0.66, 1.24)	0.54	0.70 (0.45, 1.10)	0.12	0.67 (0.28, 1.58)	0.37
	Malignant neoplasm of brain and other parts of CNS	1.10 (0.55, 2.20)	0.79	0.84 (0.31, 2.30)	0.73	0.81 (0.12, 5.43)	0.83
	Hydrocephalus	0.87 (0.63, 1.21)	0.42	0.83 (0.50, 1.35)	0.45	0.61 (0.25, 1.47)	0.28

	Craniosynostosis	0.90 (0.58, 1.41)	0.65	0.74 (0.40, 1.40)	0.36	0.74 (0.22, 2.44)	0.63
	Concussion	0.94 (0.86, 1.04)	0.23	0.95 (0.83, 1.08)	0.40	1.22 (0.95, 1.57)	0.13
	Diffuse brain injury	0.67 (0.47, 0.96)	0.03	0.77 (0.46, 1.28)	0.32	1.52 (0.59, 3.89)	0.40
	Focal brain injury	1.11 (0.84, 1.47)	0.45	1.28 (0.85, 1.93)	0.24	1.50 (0.71, 3.15)	0.30
	Congenital malformations of the nervous system	0.87 (0.40, 1.90)	0.73	0.77 (0.30, 1.95)	0.58	0.22 (0.03, 1.70)	0.16

Outcome		IVW		Weighted Median		MR-Egger	
		OR (95% CI)	P	OR (95% CI)	P	OR (95% CI)	P
PM2.5 South Asian	Trigeminal neuralgia	0.80 (0.61, 1.04)	0.11	0.80 (0.55, 1.17)	0.25	0.75 (0.43, 1.32)	0.34
	Epilepsy	1.00 (0.90, 1.10)	0.92	1.02 (0.89, 1.17)	0.79	1.10 (0.89, 1.37)	0.38
	Parkinson's disease	0.95 (0.82, 1.09)	0.47	0.95 (0.78, 1.16)	0.60	0.77 (0.58, 1.02)	0.08
	Alzheimer's disease	0.99 (0.93, 1.05)	0.76	1.06 (0.99, 1.14)	0.10	0.99 (0.88, 1.11)	0.87
	Major depressive disorder	0.97 (0.84, 1.12)	0.68	1.04 (0.85, 1.29)	0.69	1.02 (0.64, 1.63)	0.94

Table S13 Causality of the risk for PM2.5 in South Asian and Neurosurgical multisystem diseases outcomes ($p < 1 \times 10-5.$).

	Obsessive Compulsive Disorder	1.03 (0.81, 1.29)	0.83	1.09 (0.81, 1.46)	0.58	0.71 (0.43, 1.16)	0.18
	Stroke	0.97 (0.93, 1.02)	0.21	0.99 (0.93, 1.05)	0.69	0.94 (0.85, 1.04)	0.24
	Intracerebral hemorrhage	1.08 (0.94, 1.24)	0.27	1.12 (0.92, 1.36)	0.28	1.03 (0.76, 1.39)	0.87
	Subarachnoid hemorrhage	1.13 (0.94, 1.35)	0.19	1.24 (1.00, 1.53)	0.05	1.09 (0.73, 1.62)	0.68
	Transient ischemic attack	1.06 (0.95, 1.18)	0.29	1.05 (0.91, 1.21)	0.48	1.20 (0.96, 1.49)	0.12
	Cerebral infarction	1.00 (1.00, 1.00)	0.09	1.00 (1.00, 1.00)	0.31	1.00 (1.00, 1.00)	0.31
	Cerebral aneurysm	0.93 (0.80, 1.07)	0.30	0.97 (0.79, 1.18)	0.75	0.90 (0.62, 1.30)	0.59
	Cervical spondylosis	1.00 (1.00, 1.00)	0.49	1.00 (1.00, 1.00)	0.68	1.00 (1.00, 1.00)	0.60
	Spinal canal stenosis	1.10 (1.00, 1,21)	0.06	1.06 (0.95, 1.18)	0.33	1.03 (0.84, 1.25)	0.81
	spinal meningioma	1.29 (0.64, 2.57)	0.48	1.19 (0.45, 3.11)	0.73	1.46 (0.34, 6.25)	0.61
	Spinal osteochondrosis	1.46 (0.80, 2.66)	0.22	1.04 (0.45, 2.38)	0.93	0.85 (0.24, 3.02)	0.81
	Intracranial and intraspinal abscess	1.05 (0.56, 1.98)	0.88	0.90 (0.37, 2.22)	0.82	0.35 (0.09, 1.31)	0.13
	Cervical spinal cord and nerve injuries	0.65 (0.41, 1.05)	0.08	0.59 (0.30, 1.16)	0.12	0.46 (0.17, 1.24)	0.14
	Glioblastoma	1.11 (0.45, 2.75)	0.81	1.21 (0.38, 3.89)	0.75	2.56 (0.39, 16.89)	0.34
	Benign meningioma	0.94 (0.72, 1.23)	0.67	1.20 (0.84, 1.71)	0.31	0.62 (0.36, 1.06)	0.09
	Malignant meningioma	0.97 (0.72, 1.31)	0.84	1.10 (0.69, 1.75)	0.68	0.71 (0.38, 1.34)	0.31
	Pituitary adenoma and craniopharyngioma	0.87 (0.66, 1.15)	0.33	0.77 (0.51, 1.16)	0.21	0.61 (0.34, 1.09)	0.11
	Benign neoplasm of brain and other parts of CNS	0.90 (0.70, 1.15)	0.38	0.92 (0.65, 1.30)	0.64	0.81 (0.48, 1.36)	0.44
	Malignant neoplasm of brain and other parts of CNS	1.55 (0.91, 2.66)	0.11	1.51 (0.69, 3.32)	0.31	0.50 (0.16, 1.54)	0.24
	Hydrocephalus	1.12 (0.78, 1.61)	0.53	1.18 (0.78, 1.79)	0.44	1.85 (0.89, 3.85)	0.11
	Craniosynostosis	1.18 (0.80, 1.74)	0.39	1.04 (0.60, 1.81)	0.88	1.21 (0.53, 2.77)	0.66

	Concussion	1.08 (0.99, 1.16)	0.09	1.06 (0.95, 1.18)	0.29	1.06 (0.90, 1.25)	0.49
	Diffuse brain injury	0.82 (0.61, 1.11)	0.20	0.86 (0.56, 1.32)	0.48	0.91 (0.49, 1.70)	0.78
	Focal brain injury	1.04 (0.82, 1.32)	0.76	1.02 (0.72, 1.44)	0.92	1.60 (0.98, 2.61)	0.07
	Congenital malformations of the nervous system	1.33 (0.83, 2.14)	0.23	1.09 (0.54, 2.20)	0.80	3.05 (1.13, 8.24)	0.04

Outcome		IVW		Weighted Median		MR-Egger	
		OR (95% CI)	P	OR (95% CI)	P	OR (95% CI)	P
PM2.5 East Asian	Trigeminal neuralgia	0.94 (0.79, 1.12)	0.50	0.96 (0.75, 1.23)	0.77	1.07 (0.72, 1.58)	0.76
	Epilepsy	1.00 (0.94, 1.07)	0.96	0.95 (0.87, 1.04)	0.30	0.92 (0.80, 1.06)	0.30
	Parkinson's disease	0.95 (0.87, 1.03)	0.21	0.97 (0.86, 1.09)	0.62	0.89 (0.75, 1.06)	0.23
	Alzheimer's disease	0.98 (0.96, 1.01)	0.13	0.98 (0.95, 1.02)	0.36	0.98 (0.93, 1.03)	0.41
	Major depressive disorder	0.98 (0.92, 1.05)	0.65	1.02 (0.93, 1.12)	0.72	0.91 (0.79, 1.04)	0.18
	Obsessive Compulsive Disorder	0.94 (0.85, 1.04)	0.24	0.99 (0.87, 1.14)	0.93	1.05 (0.85, 1.29)	0.66

Table S14 Causality of the risk for PM2.5 in East Asian and Neurosurgical multisystem diseases outcomes (p < 1 × 10-5.).

	Stroke	1.00 (0.97, 1.02)	0.91	1.00 (0.97, 1.03)	0.91	1.00 (0.95, 1.05)	0.98
	Intracerebral hemorrhage	1.01 (0.92, 1.12)	0.74	0.98 (0.86, 1.12)	0.75	1.14 (0.91, 1.41)	0.28
	Subarachnoid hemorrhage	0.97 (0.88, 1.06)	0.49	0.94 (0.82, 1.08)	0.38	0.91 (0.74, 1.12)	0.38
	Transient ischemic attack	1.04 (0.97, 1.12)	0.24	1.04 (0.96, 1.13)	0.39	0.99 (0.85, 1.17)	0.94
	Cerebral infarction	1.00 (1.00, 1.00)	0.46	1.00 (1.00, 1.00)	0.77	1.00 (1.00, 1.00)	0.38
	Cerebral aneurysm	0.97 (0.88, 1.06)	0.49	0.98 (0.86, 1.11)	0.71	1.04 (0.82, 1.32)	0.73
	Cervical spondylosis	0.99 (0.98, 1.00)	0.01	0.99 (0.98, 1.00)	0.09	0.99 (0.98, 1.00)	0.03
	Spinal canal stenosis	0.98 (0.94, 1.03)	0.39	0.97 (0.91, 1.03)	0.35	0.97 (0.88, 1.07)	0.59
	spinal meningioma	0.98 (0.61, 1.58)	0.95	0.93 (0.48, 1.80)	0.82	0.44 (0.16, 1.22)	0.15
	Spinal osteochondrosis	1.08 (0.78, 1.48)	0.64	0.99 (0.64, 1.52)	0.95	0.81 (0.40, 1.64)	0.57
	Intracranial and intraspinal abscess	1.03 (0.61, 1.74)	0.92	0.82 (0.45, 1.50)	0.53	0.86 (0.25, 2.93)	0.81
	Cervical spinal cord and nerve injuries	0.86 (0.63, 1.17)	0.34	0.85 (0.55, 1.31)	0.46	0.72 (0.36, 1.43)	0.37
	Glioblastoma	1.06 (0.63, 1.78)	0.84	1.53 (0.75, 3.14)	0.24	1.70 (0.53, 5.42)	0.39
	Benign meningioma	1.01 (0.87, 1.17)	0.89	1.07 (0.87, 1.32)	0.53	0.99 (0.71, 1.37)	0.93
	Malignant meningioma	0.91 (0.75, 1.10)	0.33	0.92 (0.68, 1.23)	0.56	0.72 (0.46, 1.11)	0.17
	Pituitary adenoma and craniopharyngioma	0.91 (0.76, 1.09)	0.31	0.96 (0.75, 1.23)	0.74	1.08 (0.72, 1.63)	0.71
	Benign neoplasm of brain and other parts of CNS	1.09 (0.93, 1.28)	0.30	1.06 (0.84, 1.34)	0.63	0.93 (0.64, 1.34)	0.70
	Malignant neoplasm of brain and other parts of CNS	1.24 (0.87, 1.77)	0.22	1.08 (0.65, 1.82)	0.76	0.65 (0.29, 1.42)	0.30
	Hydrocephalus	1.11 (0.93, 1.34)	0.25	1.06 (0.81, 1.39)	0.68	1.10 (0.73, 1.68)	0.65
	Craniosynostosis	1.03 (0.76, 1.39)	0.85	1.10 (0.77, 1.57)	0.58	1.12 (0.56, 2.24)	0.76
	Concussion	1.03 (0.96, 1.10)	0.41	0.99 (0.92, 1.07)	0.89	1.03 (0.88, 1.19)	0.73

	Diffuse brain injury	0.95 (0.78, 1.16)	0.61	0.87 (0.67, 1.13)	0.29	0.72 (0.47, 1.11)	0.17
	Focal brain injury	1.09 (0.94, 1.27)	0.27	1.13 (0.90, 1.40)	0.29	1.14 (0.81, 1.61)	0.46
	Congenital malformations of the nervous system	1.24 (0.90, 1.70)	0.19	0.95 (0.59, 1.53)	0.83	0.86 (0.42, 1.73)	0.67

Outcome		IVW		Weighted Median		MR-Egger	
		OR (95% CI)	P	OR (95% CI)	P	OR (95% CI)	P
PM2.5 Greater Middle Eastern	Trigeminal neuralgia	0.95 (0.86, 1.05)	0.33	0.96 (0.83, 1.10)	0.54	1.18 (0.93, 1.51)	0.19
	Epilepsy	1.01 (0.97, 1.05)	0.66	1.02 (0.96, 1.07)	0.58	1.07 (0.96, 1.19)	0.24
	Parkinson's disease	0.97 (0.92, 1.02)	0.29	0.99 (0.92, 1.07)	0.87	1.05 (0.91, 1.21)	0.50
	Alzheimer's disease	1.00 (0.98, 1.02)	0.86	1.00 (0.97, 1.02)	0.84	0.98 (0.93, 1.03)	0.40
	Major depressive disorder	1.04 (0.97, 1.13)	0.29	1.08 (0.98, 1.19)	0.13	0.86 (0.61, 1.22)	0.41
	Obsessive Compulsive Disorder	0.92 (0.84, 1.01)	0.08	0.92 (0.81, 1.05)	0.23	1.01 (0.77, 1.33)	0.94
	Stroke	1.01 (0.99, 1.03)	0.43	1.01 (0.98, 1.04)	0.53	0.95 (0.89, 1.01)	0.12

Table S15 Causality of the risk for PM2.5 in Greater Middle Eastern and Neurosurgical multisystem diseases outcomes (p < 1 × 10-5.).

	Intracerebral hemorrhage	1.02 (0.97, 1.08)	0.38	1.04 (0.97, 1.12)	0.28	1.06 (0.91, 1.23)	0.45
	Subarachnoid hemorrhage	1.00 (0.93, 1.07)	0.96	1.00 (0.92, 1.09)	0.93	0.85 (0.70, 1.03)	0.10
	Transient ischemic attack	0.99 (0.96, 1.03)	0.73	1.00 (0.96, 1.05)	0.88	1.02 (0.93, 1.11)	0.73
	Cerebral infarction	1.00 (1.00, 1.00)	0.27	1.00 (1.00, 1.00)	0.77	1.00 (1.00, 1.00)	0.82
	Cerebral aneurysm	0.97 (0.92, 1.02)	0.27	0.96 (0.90, 1.03)	0.25	0.98 (0.84, 1.16)	0.85
	Cervical spondylosis	1.00 (1.00, 1.00)	0.78	1.00 (1.00, 1.00)	0.90	1.00 (1.00, 1.00)	0.11
	Spinal canal stenosis	0.98 (0.95, 1.02)	0.34	1.00 (0.95, 1.05)	0.96	1.00 (0.92, 1.10)	0.94
	spinal meningioma	0.95 (0.73, 1.23)	0.69	0.84 (0.59, 1.20)	0.34	1.11 (0.59, 2.09)	0.74
	Spinal osteochondrosis	1.00 (0.81, 1.23)	0.99	0.83 (0.62, 1.12)	0.23	1.44 (0.87, 2.39)	0.17
	Intracranial and intraspinal abscess	1.02 (0.80, 1.29)	0.89	0.97 (0.68, 1.37)	0.86	1.28 (0.71, 2.29)	0.42
	Cervical spinal cord and nerve injuries	0.98 (0.80, 1.19)	0.82	1.00 (0.78, 1.29)	0.99	0.95 (0.58, 1.54)	0.82
	Glioblastoma	1.26 (0.93, 1.69)	0.13	1.18 (0.77, 1.82)	0.44	0.85 (0.41, 1.74)	0.65
	Benign meningioma	0.96 (0.88, 1.04)	0.32	0.93 (0.82, 1.05)	0.21	1.01 (0.82, 1.24)	0.92
	Malignant meningioma	0.99 (0.88, 1.11)	0.85	0.98 (0.84, 1.14)	0.77	1.15 (0.87, 1.51)	0.33
	Pituitary adenoma and craniopharyngioma	0.99 (0.89, 1.10)	0.83	1.03 (0.89. 1.20)	0.68	0.98 (0.76, 1.26)	0.85
	Benign neoplasm of brain and other parts of CNS	0.94 (0.86, 1.03)	0.19	0.97 (0.85, 1.11)	0.67	0.97 (0.78, 1.22)	0.82
	Malignant neoplasm of brain and other parts of CNS	0.97 (0.79, 1.19)	0.78	1.00 (0.74, 1.35)	0.99	0.85 (0.51, 1.39)	0.52
	Hydrocephalus	1.02 (0.91, 1.15)	0.73	1.02 (0.88, 1.19)	0.78	1.03 (0.77, 1.38)	0.85
	Craniosynostosis	0.97 (0.84, 1.12)	0.69	0.92 (0.75, 1.13)	0.42	1.18 (0.84, 1.66)	0.35
	Concussion	1.03 (1.00, 1.06)	0.05	1.04 (1.00, 1.08)	0.04	1.05 (0.98, 1.12)	0.21
	Diffuse brain injury	1.00 (0.89, 1.13)	0.96	0.98 (0.84, 1.15)	0.82	1.01 (0.76, 1.34)	0.96

	Focal brain injury	1.03 (0.93, 1.15)	0.53	1.05 (0.92, 1.19)	0.46	0.94 (0.73, 1.20)	0.61
	Congenital malformations of the nervous system	0.84 (0.70, 1.00)	0.046	0.86 (0.68, 1.10)	0.24	0.82 (0.54, 1.25)	0.37
Outcome		IVW		Weighted Median		MR-Egger	
		OR (95% CI)	P	OR (95% CI)	P	OR (95% CI)	P
PM2.5–10 European	Trigeminal neuralgia	1.84 (0.55, 6.10)	0.32	1.15 (0.19, 6.96)	0.88	0.74 (0.12, 4.55)	0.75
	Epilepsy	1.28 (0.87, 1.90)	0.22	1.23 (0.75, 2.00)	0.41	1.27 (0.74, 2.20)	0.39
	Parkinson's disease	1.50 (0.72, 3.15)	0.28	1.64 (0.57, 4.76)	0.36	1.39 (0.43, 4.46)	0.58
	Alzheimer's disease	1.09 (0.85, 1.40)	0.50	1.07 (0.75, 1.52)	0.71	1.13 (0.66, 1.93)	0.67
	Major depressive disorder	2.77 (0.95, 8.05)	0.06	2.21 (0.46, 10.59)	0.32	66.92 (0.61, 7.30e+3)	0.09
	Obsessive Compulsive Disorder	0.74 (0.22, 2.47)	0.63	0.95 (0.17, 5.18)	0.95	1.26 (0.06, 25.72)	0.88
	Stroke	1.13 (0.81, 1.58)	0.46	1.13 (0.76, 1.68)	0.53	1.72 (0.64, 4.63)	0.30
	Intracerebral hemorrhage	0.89 (0.54, 1.49)	0.67	1.11 (0.57, 2.19)	0.75	1.22 (0.61, 2.44)	0.57

Table S16 Causality of the risk for PM2.5–10 in European and Neurosurgical multisystem diseases outcomes ($p < 1 \times 10-5$.).

	Subarachnoid hemorrhage	1.85 (1.08, 3.20)	0.03	1.43 (0.67, 3.07)	0.36	1.77 (0.83, 3.77)	0.15
	Transient ischemic attack	0.82 (0.54, 1.25)	0.36	0.93 (0.51, 1.70)	0.82	1.11 (0.59, 2.08)	0.75
	Cerebral infarction	1.00 (0.99, 1.00)	0.11	1.00 (0.99, 1.01)	0.44	1.00 (0.99, 1.01)	0.99
	Cerebral aneurysm	0.96 (0.64, 1.44)	0.85	0.86 (0.53, 1.40)	0.55	0.76 (0.45, 1.29)	0.32
	Cervical spondylosis	1.00 (0.99, 1.01)	0.24	1.00 (0.99, 1.01)	0.33	1.00 (0.99, 1.01)	0.74
	Spinal canal stenosis	0.96 (0.66, 1.39)	0.81	1.04 (0.57, 1.92)	0.89	1.21 (0.67, 2.19)	0.53
	spinal meningioma	2.75 (0.11, 72.04)	0.54	0.40 (0.00, 52.95)	0.71	0.79 (0.01, 1.19e+2)	0.93
	Spinal osteochondrosis	0.79 (0.06, 9.70)	0.85	0.40 (0.01, 21.24)	0.65	0.79 (0.02, 36.04)	0.91
	Intracranial and intraspinal abscess	0.45 (0.03, 7.73)	0.58	2.21 (0.02, 1.96e+2)	0.73	0.80 (0.01, 59.90)	0.92
	Cervical spinal cord and nerve injuries	2.51 (0.30, 21.17)	0.40	4.86 (0.14, 1.67e+2)	0.38	10.69 (0.42, 2.73e+2)	0.16
	Glioblastoma	2.71 (0.08, 94.42)	0.58	6.10 (0.02, 1.86e+3)	0.54	0.56 (2.57e-3, 1.22e+2)	0.83
	Benign meningioma	0.73 (0.26, 2.06)	0.55	0.55 (0.11, 2.87)	0.48	1.57 (0.33, 7.50)	0.58
	Malignant meningioma	0.57 (0.15, 2.20)	0.42	0.42 (0.05, 3.51)	0.42	1.44 (0.19, 11.04)	0.73
	Pituitary adenoma and craniopharyngioma	0.85 (0.24, 2.95)	0.79	1.13 (0.16, 8.00)	0.90	0.45 (0.07, 2.99)	0.42
	Benign neoplasm of brain and other parts of CNS	0.62 (0.19, 2.01)	0.43	0.64 (0.10, 3.90)	0.63	0.65 (0.11, 3.93)	0.64
	Malignant neoplasm of brain and other parts of CNS	0.64 (0.06, 7.18)	0.72	0.52 (0.01, 19.32)	0.72	0.53 (0.01, 20.99)	0.74
	Hydrocephalus	0.28 (0.08, 0.96)	0.04	0.31 (0.05, 2.13)	0.23	0.33 (0.05, 2.16)	0.25
	Craniosynostosis	0.63 (0.10, 4.05)	0.63	0.78 (0.06, 10.22)	0.85	0.26 (0.02, 4.33)	0.35
	Concussion	1.19 (0.82, 1.72)	0.37	0.97 (0.56, 1.68)	0.92	1.14 (0.65, 2.02)	0.65
	Diffuse brain injury	1.09 (0.29, 4.12)	0.89	2.41 (0.29, 19.82)	0.41	5.78 (0.78, 42.93)	0.10

	Focal brain injury	3.27 (0.96, 11.11)	0.06	1.44 (0.29, 7.20)	0.66	2.70 (0.41, 17.61)	0.31
	Congenital malformations of the nervous system	1.92 (0.17, 21.47)	0.60	1.53 (0.06, 38.28)	0.79	1.97 (0.05, 80.24)	0.72

Outcome		IVW		Weighted Median		MR-Egger	
		OR (95% CI)	P	OR (95% CI)	P	OR (95% CI)	P
PM2.5 - 10 African American or Afro-Caribbean	Trigeminal neuralgia	1.02 (0.76, 1.38)	0.89	0.91 (0.60, 1.36)	0.64	1.06 (0.36, 3.12)	0.92
	Epilepsy	1.00 (0.89, 1.11)	0.97	0.95 (0.81, 1.11)	0.50	1.24 (0.83, 1.85)	0.31
	Parkinson's disease	1.03 (0.87, 1.21)	0.76	1.12 (0.89, 1.40)	0.34	1.34 (0.74, 2.43)	0.34
	Alzheimer's disease	0.99 (0.94, 1.04)	0.73	0.98 (0.91, 1.05)	0.55	1.00 (0.84, 1.20)	0.98
	Major depressive disorder	1.01 (0.86, 1.19)	0.90	0.99 (0.79, 1.25)	0.95	0.77 (0.34, 1.73)	0.53
	Obsessive Compulsive Disorder	1.19 (0.94, 1.50)	0.16	1.14 (0.81, 1.61)	0.47	1.39 (0.58, 3.33)	0.48
	Stroke	0.96 (0.92, 1.01)	0.14	0.94 (0.88, 1.01)	0.09	0.83 (0.70, 0.99)	0.05
	Intracerebral hemorrhage	1.06 (0.88, 1.28)	0.54	1.04 (0.81, 1.32)	0.78	1.44 (0.72, 2.87)	0.31

Table S17 Causality of the risk for PM2.5 - 10 in African American or Afro-Caribbean and Neurosurgical multisystem diseases outcomes ($p < 1 \times 10^{-5}$.).

	Subarachnoid hemorrhage	0.90 (0.75, 1.08)	0.26	0.95 (0.75, 1.21)	0.68	1.20 (0.62, 2.33)	0.60
	Transient ischemic attack	0.94 (0.84, 1.06)	0.31	0.92 (0.80, 1.06)	0.23	1.02 (0.67, 1.56)	0.91
	Cerebral infarction	1.00 (1.00, 1.00)	0.43	1.00 (1.00, 1.00)	0.23	1.00 (1.00, 1.00)	0.93
	Cerebral aneurysm	0.90 (0.77, 1.05)	0.19	0.91 (0.73, 1.14)	0.42	1.36 (0.75, 2.47)	0.32
	Cervical spondylosis	1.00 (1.00, 1.00)	0.14	1.00 (1.00, 1.00)	0.87	1.00 (1.00, 1.00)	0.82
	Spinal canal stenosis	0.96 (0.87, 1.05)	0.34	0.97 (0.86, 1.10)	0.67	1.20 (0.88, 1.64)	0.26
	spinal meningioma	1.65 (0.75, 3.61)	0.21	2.28 (0.80, 6.50)	0.12	6.39 (0.38, 106.54)	0.21
	Spinal osteochondrosis	0.84 (0.45, 1.58)	0.59	1.17 (0.50, 2.71)	0.72	2.01 (0.21, 19.28)	0.55
	Intracranial and intraspinal abscess	0.98 (0.48, 2.01)	0.97	0.98 (0.36, 2.68)	0.97	0.75 (0.06, 9.74)	0.83
	Cervical spinal cord and nerve injuries	1.22 (0.72, 2.08)	0.46	0.97 (0.48, 1.95)	0.93	0.25 (0.04, 1.73)	0.18
	Glioblastoma	2.18 (0.89, 5.37)	0.09	2.97 (0.82, 10.67)	0.10	28.59 (1.16, 7.07e+2)	0.06
	Benign meningioma	1.07 (0.82, 1.41)	0.60	0.91 (0.62, 1.35)	0.65	2.34 (0.92, 5.95)	0.09
	Malignant meningioma	0.84 (0.60, 1.17)	0.30	0.80 (0.50, 1.30)	0.37	1.99 (0.59, 6.70)	0.28
	Pituitary adenoma and craniopharyngioma	1.13 (0.82, 1.54)	0.46	1.21 (0.78, 1.89)	0.39	1.63 (0.53, 5.07)	0.41
	Benign neoplasm of brain and other parts of CNS	1.15 (0.87, 1.53)	0.32	1.05 (0.71, 1.55)	0.80	1.69 (0.62, 4.64)	0.32
	Malignant neoplasm of brain and other parts of CNS	1.28 (0.65, 2.51)	0.47	1.58 (0.63, 3.96)	0.33	0.30 (0.03, 3.21)	0.33
	Hydrocephalus	0.96 (0.70, 1.31)	0.78	1.06 (0.67, 1.67)	0.81	0.89 (0.28, 2.78)	0.84
	Craniosynostosis	0.65 (0.43, 0.99)	0.046	0.68 (0.39, 1.18)	0.17	0.94 (0.21, 4.27)	0.94
	Concussion	0.94 (0.86, 1.02)	0.15	0.88 (0.78, 1.00)	0.05	0.75 (0.54, 1.03)	0.09
	Diffuse brain injury	0.89 (0.63, 1.26)	0.51	0.83 (0.52, 1.31)	0.42	0.53 (0.15, 1.83)	0.33

	Focal brain injury	0.98 (0.76, 1.28)	0.91	0.91 (0.63, 1.33)	0.64	0.43 (0.17, 1.12)	0.10
	Congenital malformations of the nervous system	0.55 (0.32, 0.93)	0.03	0.50 (0.24, 1.05)	0.07	0.66 (0.10, 4.43)	0.67

Outcome		IVW		Weighted Median		MR-Egger	
		OR (95% CI)	P	OR (95% CI)	P	OR (95% CI)	P
PM2.5–10 South Asian	Trigeminal neuralgia	0.91 (0.66, 1.27)	0.60	0.75 (0.47, 1.19)	0.23	0.86 (0.43, 1.74)	0.68
	Epilepsy	0.97 (0.85, 1.11)	0.67	1.06 (0.88, 1.28)	0.53	1.11 (0.85, 1.44)	0.45
	Parkinson's disease	1.15 (0.96, 1.38)	0.12	1.13 (0.89, 1.43)	0.31	1.17 (0.83, 1.65)	0.38
	Alzheimer's disease	1.00 (0.94, 1.07)	0.96	1.00 (0.92, 1.09)	0.99	1.01 (0.89, 1.14)	0.90
	Major depressive disorder	1.27 (0.82, 1.98)	0.28	1.21 (0.70, 2.08)	0.50	1.50 (0.55, 4.13)	0.46
	Obsessive Compulsive Disorder	1.11 (0.80, 1.55)	0.52	0.97 (0.65, 1.43)	0.86	0.87 (0.49, 1.54)	0.66
	Stroke	0.90 (0.83, 0.97)	0.01	0.87 (0.79, 0.97)	0.01	0.89 (0.76, 1.04)	0.19
	Intracerebral hemorrhage	0.94 (0.76, 1.18)	0.61	1.07 (0.80, 1.42)	0.65	1.03 (0.67, 1.58)	0.89
	Subarachnoid	1.14 (0.92,	0.23	1.03 (0.77,	0.86	1.32 (0.89,	0.19

Table S18 Causality of the risk for PM2.5–10 in South Asian and Neurosurgical multisystem diseases outcomes ($p < 1 \times 10^{-5}$.).

	hemorrhage	1.41)		1.37)		1.97)	
	Transient ischemic attack	0.88 (0.80, 0.98)	0.02	0.89 (0.77, 1.03)	0.13	0.84 (0.67, 1.04)	0.14
	Cerebral infarction	1.00 (1.00, 1.00)	0.45	1.00 (1.00, 1.00)	0.36	1.00 (1.00, 1.00)	0.89
	Cerebral aneurysm	1.01 (0.78, 1.30)	0.94	0.96 (0.68, 1.35)	0.81	0.85 (0.52, 1.39)	0.54
	Cervical spondylosis	1.01 (0.78, 1.30)	0.94	0.96 (0.68, 1.35)	0.80	0.85 (0.52, 1.39)	0.54
	Spinal canal stenosis	1.04 (0.92, 1.17)	0.55	0.99 (0.86, 1.14)	0.90	0.97 (0.76, 1.22)	0.78
	spinal meningioma	0.60 (0.25, 1.41)	0.24	0.51 (0.17, 1.56)	0.24	0.88 (0.15, 5.13)	0.89
	Spinal osteochondrosis	0.88 (0.38, 2.06)	0.77	1.17 (0.41, 3.28)	0.77	0.53 (0.09, 3.14)	0.50
	Intracranial and intraspinal abscess	0.83 (0.38, 1.83)	0.65	0.74 (0.26, 2.07)	0.56	0.23 (0.05, 1.16)	0.10
	Cervical spinal cord and nerve injuries	1.07 (0.60, 1.94)	0.81	1.09 (0.51, 2.32)	0.83	1.03 (0.31, 3.45)	0.96
	Glioblastoma	0.94 (0.35, 2.52)	0.91	1.03 (0.26, 4.05)	0.97	0.91 (0.12, 6.74)	0.93
	Benign meningioma	0.88 (0.62, 1.27)	0.50	1.02 (0.70, 1.48)	0.93	0.71 (0.33, 1.51)	0.39
	Malignant meningioma	1.08 (0.75, 1.57)	0.67	1.01 (0.62, 1.65)	0.97	0.79 (0.37, 1.69)	0.56
	Pituitary adenoma and craniopharyngioma	0.88 (0.62, 1.24)	0.47	0.94 (0.58, 1.52)	0.80	0.85 (0.40, 1.79)	0.68
	Benign neoplasm of brain and other parts of CNS	1.15 (0.85, 1.57)	0.37	1.16 (0.76, 1.79)	0.49	1.38 (0.73, 2.62)	0.35
	Malignant neoplasm of brain and other parts of CNS	1.53 (0.79, 2.97)	0.21	2.10 (0.86, 5.12)	0.10	2.96 (0.76, 11.54)	0.15
	Hydrocephalus	1.05 (0.74, 1.48)	0.78	1.00 (0.62, 1.61)	0.99	0.83 (0.41, 1.67)	0.61
	Craniosynostosis	1.26 (0.79, 2.01)	0.32	1.26 (0.70, 2.27)	0.44	1.43 (0.55, 3.70)	0.48
	Concussion	1.06 (0.96, 1.17)	0.23	1.07 (0.94, 1.22)	0.32	1.12 (0.92, 1.37)	0.29
	Diffuse brain injury	0.89 (0.59, 1.34)	0.57	1.15 (0.69, 1.93)	0.59	0.66 (0.28, 1.56)	0.37
	Focal brain injury	0.97 (0.65, 1.43)	0.87	0.99 (0.62, 1.57)	0.96	1.04 (0.45, 2.41)	0.93

	Congenital malformations of the nervous system	1.17 (0.65, 2.10)	0.61	0.94 (0.41, 2.17)	0.88	1.17 (0.35, 3.90)	0.80

Table S19 Causality of the risk for PM2.5－10 in East Asian and Neurosurgical multisystem diseases outcomes ($p < 1 \times 10^{-5}$.).

Outcome		IVW		Weighted Median		MR-Egger	
		OR (95% CI)	P	OR (95% CI)	P	OR (95% CI)	P
PM2.5－10 East Asian	Trigeminal neuralgia	1.02 (0.90, 1.15)	0.77	1.04 (0.89, 1.21)	0.65	0.97 (0.79, 1.20)	0.80
	Epilepsy	1.01 (0.95, 1.08)	0.66	1.05 (0.98, 1.14)	0.17	1.02 (0.92, 1.14)	0.69
	Parkinson's disease	1.01 (0.94, 1.09)	0.73	1.07 (0.98, 1.17)	0.14	1.06 (0.95, 1.20)	0.34
	Alzheimer's disease	0.99 (0.97, 1.01)	0.45	0.99 (0.97, 1.02)	0.71	1.01 (0.98, 1.05)	0.46
	Major depressive disorder	1.06 (0.99, 1.14)	0.12	1.08 (0.98, 1.20)	0.13	1.10 (0.96, 1.25)	0.21
	Obsessive Compulsive Disorder	0.96 (0.88, 1.05)	0.42	0.98 (0.87, 1.10)	0.70	0.92 (0.78, 1.07)	0.33
	Stroke	1.01 (0.99, 1.04)	0.21	1.00 (0.97, 1.03)	0.95	1.01 (0.97, 1.06)	0.59
	Intracerebral hemorrhage	1.00 (0.92, 1.07)	0.91	0.99 (0.89, 1.10)	0.78	0.92 (0.81, 1.05)	0.24
	Subarachnoid hemorrhage	1.02 (0.94, 1.10)	0.70	1.00 (0.90, 1.12)	0.97	0.98 (0.86, 1.13)	0.80
	Transient ischemic attack	0.99 (0.95, 1.03)	0.66	1.00 (0.95, 1.06)	0.89	1.00 (0.93, 1.07)	0.96
	Cerebral infarction	1.00 (1.00, 1.00)	0.15	1.00 (1.00, 1.00)	0.49	1.00 (1.00, 1.00)	0.65
	Cerebral aneurysm	1.00 (0.91, 1.10)	0.94	1.00 (0.88, 1.14)	0.99	1.15 (0.97, 1.37)	0.15
	Cervical spondylosis	1.00 (1.00, 1.00)	0.20	1.00 (1.00, 1.00)	0.12	1.00 (1.00, 1.00)	0.27
	Spinal canal stenosis	1.01 (0.98, 1.05)	0.46	1.02 (0.97, 1.07)	0.39	1.04 (0.98, 1.11)	0.24
	spinal meningioma	1.04 (0.74, 1.46)	0.83	0.97 (0.63, 1.48)	0.88	0.71 (0.42, 1.22)	0.26
	Spinal osteochondrosis	0.59 (0.44, 0.79)	0.0003	0.66 (0.45, 0.98)	0.04	0.60 (0.36, 1.01)	0.10
	Intracranial and intraspinal abscess	1.06 (0.79, 1.42)	0.69	1.10 (0.74, 1.65)	0.63	1.19 (0.72, 1.94)	0.52
	Cervical spinal cord and nerve injuries	1.03 (0.83, 1.28)	0.77	1.11 (0.85, 1.44)	0.45	0.89 (0.62, 1.29)	0.57
	Glioblastoma	0.81 (0.56, 1.16)	0.24	0.91 (0.56, 1.48)	0.70	0.94 (0.51, 1.73)	0.84
	Benign meningioma	1.00 (0.85, 1.16)	0.97	0.93 (0.81, 1.06)	0.27	0.84 (0.66, 1.05)	0.17
	Malignant meningioma	0.99 (0.82,	0.89	0.99 (0.92,	0.89	0.88 (0.64,	0.46

		1.19)		1.19)		1.21)	
	Pituitary adenoma and craniopharyngioma	0.95 (0.82, 1.10)	0.51	1.01 (0.85, 1.21)	0.88	1.01 (0.78, 1.32)	0.92
	Benign neoplasm of brain and other parts of CNS	0.98 (0.85, 1.12)	0.76	0.97 (0.82, 1.15)	0.72	0.89 (0.71, 1.12)	0.36
	Malignant neoplasm of brain and other parts of CNS	1.15 (0.86, 1.56)	0.35	1.11 (0.80, 1.53)	0.53	0.91 (0.55, 1.49)	0.71
	Hydrocephalus	1.02 (0.89, 1.17)	0.77	0.99 (0.83, 1.18)	0.90	0.37 (1.17, 0.95)	0.19
	Craniosynostosis	0.89 (0.75, 1.05)	0.17	0.90 (0.74, 1.11)	0.33	0.91 (0.68, 1.21)	0.53
	Concussion	1.01 (0.97, 1.04)	0.73	1.00 (0.96, 1.05)	0.92	0.99 (0.93, 1.05)	0.80
	Diffuse brain injury	0.90 (0.78, 1.02)	0.11	0.90 (0.76, 1.07)	0.23	0.91 (0.72, 1.14)	0.44
	Focal brain injury	1.00 (0.90, 1.11)	0.97	1.01 (0.86, 1.19)	0.86	1.04 (0.87, 1.25)	0.67
	Congenital malformations of the nervous system	0.90 (0.73, 1.12)	0.35	0.82 (0.62, 1.08)	0.16	0.89 (0.61, 1.28)	0.54

Outcome	IVW	Weighted Median	MR-Egger

Table S20 Causality of the risk for PM2.5 - 10 in Greater Middle Eastern and Neurosurgical multisystem diseases outcomes ($p < 1 \times 10-5.$).

		OR (95% CI)	P	OR (95% CI)	P	OR (95% CI)	P
PM2.5‐10 Greater Middle Eastern	Trigeminal neuralgia	1.05 (0.95, 1.16)	0.34	1.03 (0.89, 1.19)	0.67	1.06 (0.86, 1.30)	0.60
	Epilepsy	0.99 (0.95, 1.03)	0.59	0.99 (0.94, 1.04)	0.68	1.01 (0.93, 1.10)	0.82
	Parkinson's disease	0.98 (0.92, 1.04)	0.51	0.95 (0.92, 1.04)	0.25	0.96 (0.84, 1.09)	0.53
	Alzheimer's disease	1.00 (0.97, 1.02)	0.71	1.00 (0.97, 1.03)	0.84	1.00 (0.94, 1.05)	0.87
	Major depressive disorder	0.98 (0.91, 1.05)	0.58	1.01 (0.91, 1.11)	0.86	1.20 (0.79, 1.81)	0.40
	Obsessive Compulsive Disorder	0.96 (0.87, 1.06)	0.45	0.98 (0.85, 1.13)	0.79	0.85 (0.59, 1.21)	0.38
	Stroke	1.02 (1.00, 1.05)	0.01	1.03 (1.00, 1.06)	0.07	0.97 (0.92, 1.03)	0.34
	Intracerebral hemorrhage	0.99 (0.94, 1.05)	0.82	1.00 (0.92, 1.08)	0.97	0.92 (0.82, 1.02)	0.12
	Subarachnoid hemorrhage	1.06 (1.00, 1.12)	0.04	1.08 (0.99, 1.18)	0.10	1.12 (1.00, 1.25)	0.07
	Transient ischemic attack	0.99 (0.96, 1.03)	0.70	1.01 (0.96, 1.06)	0.71	1.02 (0.95, 1.09)	0.62
	Cerebral infarction	1.00 (1.00, 1.00)	0.61	1.00 (1.00, 1.00)	0.80	1.00 (1.00, 1.00)	0.10
	Cerebral aneurysm	1.01 (0.96, 1.06)	0.83	1.05 (0.97, 1.13)	0.26	1.04 (0.95, 1.14)	0.42
	Cervical spondylosis	1.00 (1.00, 1.00)	0.47	1.00 (1.00, 1.00)	0.41	1.00 (1.00, 1.00)	0.80
	Spinal canal stenosis	0.99 (0.96, 1.02)	0.38	1.01 (0.96, 1.05)	0.81	0.96 (0.90, 1.02)	0.21
	spinal meningioma	0.86 (0.65, 1.14)	0.28	0.74 (0.50, 1.09)	0.12	0.58 (0.33, 1.01)	0.07
	Spinal osteochondrosis	0.96 (0.78, 1.19)	0.72	0.92 (0.68, 1.25)	0.58	1.00 (0.65, 1.54)	0.99
	Intracranial and intraspinal abscess	1.11 (0.87, 1.41)	0.39	0.95 (0.65, 1.39)	0.78	1.10 (0.67, 1.80)	0.71
	Cervical spinal cord and nerve injuries	0.96 (0.80, 1.17)	0.70	0.90 (0.68, 1.19)	0.47	0.82 (0.56, 1.22)	0.34
	Glioblastoma	0.93 (0.66, 1.30)	0.66	1.02 (0.64, 1.63)	0.92	0.94 (0.47, 1.89)	0.86
	Benign meningioma	0.98 (0.90, 1.06)	0.58	0.93 (0.82, 1.06)	0.30	0.88 (0.74, 1.05)	0.16
	Malignant meningioma	0.96 (0.85, 1.07)	0.44	0.89 (0.75, 1.05)	0.16	0.95 (0.75, 1.19)	0.65

	Pituitary adenoma and craniopharyngioma	0.94 (0.84, 1.04)	0.22	0.94 (0.80, 1.11)	0.48	0.87 (0.70, 1.08)	0.21
	Benign neoplasm of brain and other parts of CNS	1.02 (0.90, 1.15)	0.75	1.09 (0.95, 1.25)	0.22	1.18 (0.93, 1.50)	0.18
	Malignant neoplasm of brain and other parts of CNS	1.07 (0.88, 1.32)	0.49	1.00 (0.74, 1.34)	0.98	1.16 (0.76, 1.78)	0.49
	Hydrocephalus	0.96 (0.86, 1.06)	0.40	0.97 (0.84, 1.12)	0.70	0.94 (0.76, 1.17)	0.59
	Craniosynostosis	1.04 (0.90, 1.20)	0.59	1.06 (0.86, 1.31)	0.59	1.02 (0.76, 1.37)	0.91
	Concussion	0.98 (0.94, 1.02)	0.31	0.98 (0.93, 1.02)	0.30	0.97 (0.90, 1.05)	0.50
	Diffuse brain injury	1.01 (0.89, 1.14)	0.88	0.96 (0.81, 1.14)	0.65	1.03 (0.80, 1.34)	0.80
	Focal brain injury	0.91 (0.82, 1.01)	0.09	0.87 (0.76, 1.00)	0.04	0.85 (0.68, 1.06)	0.17
	Congenital malformations of the nervous system	0.97 (0.81, 1.17)	0.78	1.02 (0.79, 1.31)	0.89	1.12 (0.77, 1.61)	0.56

Outcome		IVW		Weighted Median		MR-Egger	
		OR (95% CI)	P	OR (95% CI)	P	OR (95% CI)	P

Table S21 Causality of the risk for PM10 in European and Neurosurgical multisystem diseases outcomes (p < 1 × 10-5.).

PM10 European	Trigeminal neuralgia	2.65 (0.38, 18.44)	0.32	2.61 (0.15, 43.96)	0.51	1.62 (0.01, 2.70e+2)	0.86
	Epilepsy	0.82 (0.37, 1.85)	0.64	0.73 (0.26, 2.02)	0.54	0.92 (0.10, 8.06)	0.94
	Parkinson's disease	0.56 (0.19, 1.69)	0.31	0.75 (0.18, 3.11)	0.70	0.22 (0.01, 3.92)	0.32
	Alzheimer's disease	1.25 (0.79, 1.98)	0.34	1.39 (0.87, 2.24)	0.17	0.51 (0.16, 1.61)	0.27
	Major depressive disorder	0.76 (0.24, 2.39)	0.64	0.64 (0.15, 2.73)	0.54	1.03 (0.02, 67.03)	0.99
	Obsessive Compulsive Disorder	2.72 (0.70, 10.56)	0.15	5.11 (0.74, 35.31)	0.10	2.06 (0.04, 97.92)	0.72
	Stroke	1.17 (0.79, 1.73)	0.43	0.79 (0.48, 1.31)	0.36	0.80 (0.25, 2.52)	0.71
	Intracerebral hemorrhage	1.24 (0.48, 3.21)	0.66	0.92 (0.26, 3.28)	0.89	0.79 (0.06, 9.66)	0.85
	Subarachnoid hemorrhage	1.54 (0.51, 4.68)	0.44	2.46 (0.61, 10.01)	0.21	0.42 (0.02, 7.74)	0.56
	Transient ischemic attack	0.72 (0.40, 1.32)	0.29	0.90 (0.40, 2.00)	0.79	2.13 (0.45, 10.10)	0.35
	Cerebral infarction	1.01 (1.00, 1.01)	0.25	1.00 (0.99, 1.01)	0.55	1.00 (0.98, 1.02)	0.97
	Cerebral aneurysm	0.73 (0.29, 1.81)	0.49	0.76 (0.21, 2.81)	0.68	1.60 (0.13, 20.05)	0.72
	Cervical spondylosis	1.00 (0.99, 1.00)	0.44	1.00 (0.99, 1.01)	0.90	0.99 (0.97, 1.01)	0.49
	Spinal canal stenosis	0.40 (0.23, 0.70)	0.001	0.35 (0.16, 0.77)	0.009	0.27 (0.07, 1.12)	0.09
	spinal meningioma	0.87 (6.55e-3, 1.16e+2)	0.96	9.19 (1.10e-2, 7.64e+3)	0.52	1.14 (3.92e-6, 3.32e+5)	0.98
	Spinal osteochondrosis	10.26 (0.20, 5.25e+2)	0.25	15.45 (0.08, 3.12e+3)	0.31	1.18e+2 (4.56e-3, 3.03e+6)	0.37
	Intracranial and intraspinal abscess	17.13 (0.15, 1.98e+3)	0.24	1.23e+2 (0.17, 8.93e+4)	0.15	8.97e-2 (4.10e-7, 1.96e+4)	0.71
	Cervical spinal cord and nerve injuries	0.06 (2.18e-3, 1.72)	0.10	0.10 (1.04e-3, 10.57)	0.34	0.09 (1.64e5, 4.87e+2)	0.59
	Glioblastoma	11.12 (4.36e-2, 2.83e+3)	0.39	1.81e+2 (0.11, 3.12e+5)	0.17	0.05 (3.10e-8, 7.31e+4)	0.68

	Benign meningioma	1.56 (0.24, 10.05)	0.64	3.17 (0.34, 29.77)	0.31	0.14 (0.01, 16.71)	0.43
	Malignant meningioma	2.96 (0.31, 28.42)	0.35	0.87 (0.05, 16.50)	0.92	0.07 (2.35e-4, 19.87)	0.37
	Pituitary adenoma and craniopharyngioma	0.73 (0.10, 5.14)	0.75	0.76 (0.05, 10.51)	0.84	0.52 (3.34e-3, 79.60)	0.80
	Benign neoplasm of brain and other parts of CNS	0.61 (0.11, 3.54)	0.58	0.79 (0.07, 8.63)	0.84	5.48 (0.06, 5.07e+2)	0.47
	Malignant neoplasm of brain and other parts of CNS	5.35 (0.12, 2.33e+2)	0.38	3.29 (0.02, 6.95e+2)	0.66	2.59e+2 (1.53e-2, 4.38e+6)	0.28
	Hydrocephalus	0.49 (0.07, 3.46)	0.48	0.30 (0.02, 4.37)	0.38	0.02 (1.57e-4, 3.66)	0.16
	Craniosynostosis	1.01 (0.07, 14.43)	0.99	1.49 (0.04, 63.00)	0.83	4.37 (3.96e-3, 4.82e+3)	0.68
	Concussion	0.97 (0.52, 1.83)	0.93	1.18 (0.51, 2.74)	0.70	1.53 (0.29, 7.97)	0.62
	Diffuse brain injury	1.36 (0.15, 12.75)	0.79	3.69 (0.18, 77.62)	0.40	0.07 (2.20e-4, 20.10)	0.36
	Focal brain injury	5.87 (0.87, 39.65)	0.07	9.90 (0.95, 1.01e+2)	0.05	3.51 (0.02, 5.42e+2)	0.63
	Congenital malformations of the nervous system	2.42 (0.08, 69.17)	0.61	1.10 (1.00e-3, 1.20e+2)	0.97	2.56 (3.66e-4, 1.78e+4)	0.84

Outcome		IVW		Weighted Median		MR-Egger	
		OR (95% CI)	P	OR (95% CI)	P	OR (95% CI)	P

Table S22 Causality of the risk for PM10 in African American or Afro-Caribbean and Neurosurgical multisystem diseases outcomes ($p < 1 \times 10-5.$).

PM10 African American or Afro-Caribbean	Trigeminal neuralgia	0.82 (0.55, 1.21)	0.31	0.68 (0.41, 1.15)	0.15	0.78 (0.22, 2.77)	0.70
	Epilepsy	1.08 (0.96, 1.22)	0.19	1.08 (0.91, 1.27)	0.37	1.02 (0.70, 1.50)	0.91
	Parkinson's disease	0.88 (0.73, 1.05)	0.16	0.91 (0.72, 1.16)	0.45	1.42 (0.79, 2.58)	0.26
	Alzheimer's disease	0.99 (0.93, 1.06)	0.84	0.98 (0.90, 1.08)	0.71	1.25 (1.03, 1.51)	0.04
	Major depressive disorder	0.89 (0.75, 1.07)	0.21	0.96 (0.74, 1.23)	0.72	1.24 (0.58, 2.63)	0.59
	Obsessive Compulsive Disorder	0.87 (0.67, 1.12)	0.27	0.88 (0.61, 1.27)	0.50	0.73 (0.31, 1.69)	0.47
	Stroke	0.95 (0.90, 1.00)	0.07	0.94 (0.87, 1.02)	0.13	1.01 (0.83, 1.22)	0.94
	Intracerebral hemorrhage	0.98 (0.80, 1.21)	0.88	1.00 (0.77, 1.29)	0.98	0.82 (0.42, 1.60)	0.56
	Subarachnoid hemorrhage	1.05 (0.87, 1.25)	0.63	1.05 (0.81, 1.36)	0.70	1.17 (0.66, 2.08)	0.61
	Transient ischemic attack	0.91 (0.80, 1.03)	0.14	0.94 (0.79, 1.11)	0.44	1.18 (0.80, 1.73)	0.42
	Cerebral infarction	1.00 (1.00, 1.00)	0.33	1.00 (1.00, 1.00)	0.82	1.00 (1.00, 1.01)	0.78
	Cerebral aneurysm	1.01 (0.84, 1.22)	0.89	1.00 (0.80, 1.27)	0.97	0.93 (0.51, 1.70)	0.82
	Cervical spondylosis	1.00 (1.00, 1.00)	0.89	1.00 (1.00, 1.00)	0.90	1.00 (0.99, 1.01)	0.99
	Spinal canal stenosis	0.98 (0.88, 1.09)	0.72	0.92 (0.79, 1.07)	0.28	1.00 (0.71, 1.39)	0.98
	spinal meningioma	0.74 (0.28, 1.93)	0.54	0.73 (0.20, 2.63)	0.63	0.81 (0.04, 15.68)	0.89
	Spinal osteochondrosis	0.85 (0.27, 2.71)	0.79	0.90 (0.28, 2.84)	0.85	1.10 (0.03, 46.37)	0.96
	Intracranial and intraspinal abscess	2.32 (0.92, 5.87)	0.08	2.50 (0.71, 8.87)	0.16	0.53 (0.03, 9.29)	0.67
	Cervical spinal cord and nerve injuries	0.71 (0.33, 1.50)	0.37	0.53 (0.20, 1.40)	0.20	0.33 (0.03, 3.60)	0.38
	Glioblastoma	0.64 (0.21, 1.92)	0.42	0.49 (0.10, 2.38)	0.38	0.23 (0.01, 7.81)	0.43
	Benign meningioma	0.93 (0.68, 1.27)	0.65	0.90 (0.57, 1.42)	0.65	1.90 (0.72, 4.96)	0.22
	Malignant meningioma	1.15 (0.76, 1.74)	0.52	1.06 (0.59, 1.92)	0.84	1.41 (0.37, 5.40)	0.62
	Pituitary adenoma and craniopharyngioma	0.90 (0.60, 1.35)	0.62	1.11 (0.63, 1.95)	0.73	0.52 (0.15, 1.87)	0.34

	Benign neoplasm of brain and other parts of CNS	1.25 (0.88, 1.76)	0.21	1.16 (0.74, 1.83)	0.52	1.65 (0.57, 4.80)	0.38
	Malignant neoplasm of brain and other parts of CNS	1.14 (0.49, 2.66)	0.76	0.90 (0.29, 2.76)	0.85	0.67 (0.04, 10.26)	0.78
	Hydrocephalus	1.18 (0.80, 1.73)	0.40	1.31 (0.79, 2.17)	0.30	1.82 (0.56, 5.94)	0.34
	Craniosynostosis	0.90 (0.45, 1.80)	0.76	0.65 (0.31, 1.35)	0.25	0.34 (0.04, 2.91)	0.34
	Concussion	1.00 (0.90, 1.11)	0.98	1.00 (0.86, 1.15)	0.96	1.07 (0.76, 1.50)	0.71
	Diffuse brain injury	0.88 (0.56, 1.39)	0.58	0.89 (0.52, 1.52)	0.68	1.18 (0.27, 5.09)	0.83
	Focal brain injury	1.04 (0.75, 1.44)	0.80	1.11 (0.72, 1.72)	0.64	1.15 (0.42, 3.14)	0.78
	Congenital malformations of the nervous system	0.52 (0.27, 1.02)	0.06	0.69 (0.26, 1.79)	0.44	0.12 (0.02, 0.87)	0.06

Outcome		IVW		Weighted Median		MR-Egger	
		OR (95% CI)	P	OR (95% CI)	P	OR (95% CI)	P
PM10 East Asian	Trigeminal neuralgia	1.03 (0.86, 1.24)	0.77	1.06 (0.82, 1.36)	0.68	1.01 (0.69, 1.48)	0.95

Table S23 Causality of the risk for PM10 in East Asian and Neurosurgical multisystem diseases outcomes ($p < 1 \times 10-5.$).

	Epilepsy	1.04 (0.97, 1.12)	0.29	1.05 (0.95, 1.16)	0.31	0.98 (0.84, 1.16)	0.85
	Parkinson's disease	0.99 (0.90, 1.10)	0.88	1.03 (0.89, 1.19)	0.69	1.13 (0.90. 1.41)	0.33
	Alzheimer's disease	0.98 (0.94, 1.03)	0.38	0.99 (0.95, 1.03)	0.66	0.99 (0.89, 1.10)	0.85
	Major depressive disorder	0.98 (0.86, 1.11)	0.75	1.04 (0.90, 1.19)	0.59	0.97 (0.58, 1.64)	0.92
	Obsessive Compulsive Disorder	1.12 (0.92, 1.37)	0.27	1.14 (0.90, 1.44)	0.29	1.12 (0.64, 1.96)	0.72
	Stroke	1.01 (0.96, 1.06)	0.70	1.05 (1.00, 1.11)	0.05	1.10 (1.00, 1.20)	0.08
	Intracerebral hemorrhage	1.02 (0.92, 1.14)	0.65	1.04 (0.90, 1.20)	0.58	1.05 (0.82, 1.34)	0.73
	Subarachnoid hemorrhage	1.01 (0.90, 1.13)	0.91	1.05 (0.89, 1.23)	0.57	1.03 (0.79, 1.36)	0.81
	Transient ischemic attack	0.98 (0.92, 1.04)	0.46	1.02 (0.94, 1.11)	0.65	0.89 (0.79, 1.01)	0.12
	Cerebral infarction	1.00 (1.00, 1.00)	0.41	1.00 (1.00, 1.00)	0.68	1.00 (1.00, 1.00)	0.63
	Cerebral aneurysm	0.99 (0.89, 1.11)	0.89	0.99 (0.85, 1.16)	0.94	0.96 (0.69, 1.33)	0.82
	Cervical spondylosis	1.00 (1.00, 1.00)	0.04	1.00 (1.00, 1.00)	0.03	1.00 (1.00, 1.00)	0.10
	Spinal canal stenosis	0.99 (0.94, 1.05)	0.84	1.00 (0.93, 1.08)	0.96	1.01 (0.90, 1.15)	0.83
	spinal meningioma	0.98 (0.54, 1.76)	0.94	0.63 (0.30, 1.32)	0.22	0.39 (0.14, 1.14)	0.13
	Spinal osteochondrosis	1.43 (0.98, 2.10)	0.07	1.38 (0.82, 2.32)	0.22	1.14 (0.51, 2.54)	0.75
	Intracranial and intraspinal abscess	1.11 (0.69, 1.79)	0.67	0.97 (0.55, 1.70)	0.92	0.75 (0.27, 2.04)	0.59
	Cervical spinal cord and nerve injuries	1.02 (0.74, 1.41)	0.91	0.98 (0.62, 1.54)	0.93	0.74 (0.38, 1.47)	0.42
	Glioblastoma	1.25 (0.62, 2.53)	0.53	1.05 (0.50, 2.18)	0.90	0.60 (0.14, 2.55)	0.51
	Benign meningioma	1.01 (0.87, 1.18)	0.88	1.03 (0.85, 1.26)	0.76	0.92 (0.66, 1.26)	0.61
	Malignant meningioma	0.92 (0.75, 1.13)	0.44	0.85 (0.65, 1.12)	0.25	0.75 (0.49, 1.15)	0.23
	Pituitary adenoma and craniopharyngioma	1.02 (0.85, 1.24)	0.81	1.10 (0.86, 1.41)	0.44	1.13 (0.76, 1.69)	0.56
	Benign neoplasm of brain and other parts	1.10 (0.92, 1.32)	0.30	1.07 (0.85, 1.34)	0.58	0.95 (0.65, 1.40)	0.82

	of CNS						
	Malignant neoplasm of brain and other parts of CNS	0.97 (0.66, 1.44)	0.89	1.00 (0.60, 1.67)	0.99	1.03 (0.43, 2.46)	0.96
	Hydrocephalus	1.05 (0.87, 1.27)	0.61	0.99 (0.77, 1.27)	0.94	0.80 (0.54, 1.19)	0.30
	Craniosynostosis	0.82 (0.63, 1.05)	0.12	0.84 (0.60, 1.19)	0.32	0.76 (0.45, 1.30)	0.35
	Concussion	1.01 (0.96, 1.07)	0.64	1.03 (0.96, 1.10)	0.48	1.10 (0.98, 1.23)	0.15
	Diffuse brain injury	1.05 (0.81, 1.37)	0.69	0.97 (0.70, 1.35)	0.86	0.70 (0.44, 1.13)	0.19
	Focal brain injury	1.02 (0.87, 1.20)	0.80	0.97 (0.77, 1.21)	0.77	0.83 (0.60, 1.16)	0.31
	Congenital malformations of the nervous system	1.05 (0.65, 1.70)	0.85	0.91 (0.58, 1.43)	0.68	0.97 (0.33, 2.83)	0.95

Outcome		IVW		Weighted Median		MR-Egger	
		OR (95% CI)	P	OR (95% CI)	P	OR (95% CI)	P
PM10 Greater Middle Eastern	Trigeminal neuralgia	1.05 (0.93, 1.20)	0.43	1.03 (0.86, 1.23)	0.77	1.06 (0.80, 1.40)	0.70
	Epilepsy	0.98 (0.94, 1.03)	0.53	1.00 (0.93, 1.07)	0.96	0.96 (0.86, 1.08)	0.51

Table S24 Causality of the risk for PM10 in Greater Middle Eastern and Neurosurgical multisystem diseases outcomes ($p < 1 \times 10-5.$).

	Parkinson's disease	0.92 (0.86, 0.99)	0.02	0.90 (0.82, 0.99)	0.03	0.91 (0.78, 1.05)	0.22
	Alzheimer's disease	0.97 (0.95, 1.00)	0.04	0.99 (0.06, 1.02)	0.40	1.00 (0.95, 1.05)	0.99
	Major depressive disorder	0.97 (0.89, 1.06)	0.54	0.98 (0.88, 1.09)	0.70	1.21 (0.69. 2.11)	0.52
	Obsessive Compulsive Disorder	0.93 (0.83, 1.05)	0.23	0.98 (0.83, 1.15)	0.79	0.96 (0.63, 1.47)	0.87
	Stroke	1.00 (0.97, 1.02)	0.84	1.00 (0.97, 1.04)	0.90	0.97 (0.91, 1.04)	0.43
	Intracerebral hemorrhage	0.96 (0.89, 1.03)	0.26	0.97 (0.88, 1.07)	0.54	0.96 (0.81, 1.13)	0.64
	Subarachnoid hemorrhage	0.94 (0.86, 1.03)	0.16	0.96 (0.85, 1.07)	0.44	1.05 (0.86. 1.28)	0.62
	Transient ischemic attack	1.02 (0.98, 1.06)	0.42	1.01 (0.95, 1.07)	0.74	0.98 (0.90, 1.08)	0.69
	Cerebral infarction	1.00 (1.00, 1.00)	0.82	1.00 (1.00, 1.00)	0.25	1.00 (1.00, 1.00)	0.56
	Cerebral aneurysm	1.05 (0.97, 1.13)	0.26	1.02 (0.92, 1.13)	0.69	1.17 (0.95, 1.44)	0.16
	Cervical spondylosis	1.00 (1.00, 1.00)	0.48	1.00 (1.00, 1.00)	0.19	1.00 (1.00, 1.00)	0.47
	Spinal canal stenosis	0.99 (0.95, 1.03)	0.60	0.98 (0.93, 1.03)	0.40	0.93 (0.85, 1.00)	0.09
	spinal meningioma	1.04 (0.73, 1.49)	0.83	0.93 (0.57, 1.50)	0.76	0.73 (0.33, 1.61)	0.45
	Spinal osteochondrosis	1.07 (0.81, 1.43)	0.63	1.00 (0.66, 1.52)	0.99	2.01 (1.11, 3.62)	0.04
	Intracranial and intraspinal abscess	1.05 (0.76, 1.46)	0.75	0.96 (0.61, 1.51)	0.87	0.94 (0.45, 1.98)	0.88
	Cervical spinal cord and nerve injuries	0.93 (0.74, 1.16)	0.51	0.94 (0.67, 1.31)	0.71	0.97 (0.59, 1.60)	0.91
	Glioblastoma	0.74 (0.50, 1.07)	0.11	0.76 (0.46, 1.28)	0.30	0.70 (0.30, 1.61)	0.41
	Benign meningioma	1.14 (1.02, 1.26)	0.02	1.18 (1.02, 1.38)	0.03	0.96 (0.75, 1.22)	0.73
	Malignant meningioma	1.05 (0.91, 1.21)	0.49	1.07 (0.86, 1.31)	0.55	1.00 (0.73, 1.37)	0.99
	Pituitary adenoma and craniopharyngioma	0.94 (0.79, 1.12)	0.51	0.98 (0.81, 1.19)	0.83	0.92 (0.62, 1.36)	0.68
	Benign neoplasm of brain and other parts of CNS	1.01 (0.89, 1.16)	0.86	1.10 (0.93, 1.30)	0.28	1.00 (0.74, 1.36)	0.98
	Malignant neoplasm of	0.98 (0.76,	0.86	0.88 (0.62,	0.49	0.75 (0.43,	0.34

	brain and other parts of CNS	1.26)		1.26)		1.32)	
	Hydrocephalus	1.11 (0.98, 1.27)	0.11	1.12 (0.92, 1.36)	0.25	1.07 (0.79, 1.44)	0.67
	Craniosynostosis	0.91 (0.76, 1.08)	0.28	0.87 (0.67, 1.13)	0.30	1.00 (0.67, 1.50)	0.98
	Concussion	0.99 (0.95, 1.03)	0.64	1.00 (0.95, 1.06)	0.96	0.99 (0.91, 1.08)	0.86
	Diffuse brain injury	1.01 (0.88, 1.17)	0.87	0.93 (0.77, 1.14)	0.48	0.94 (0.69, 1.29)	0.73
	Focal brain injury	0.93 (0.83, 1.05)	0.25	0.96 (0.82, 1.13)	0.65	0.84 (0.65, 1.08)	0.20
	Congenital malformations of the nervous system	0.92 (0.73, 1.15)	0.45	0.88 (0.64, 1.21)	0.43	0.83 (0.50, 1.38)	0.48

Outcome		IVW		Weighted Median		MR-Egger	
		OR (95% CI)	P	OR (95% CI)	P	OR (95% CI)	P
PM2.5absorbance European	Trigeminal neuralgia	0.38 (5.08e-3, 28.90)	0.66	0.13 (1.08e-3, 14.62)	0.39	0.01 (1.73e-6, 68.05)	0.50
	Epilepsy	2.30 (0.27, 19.64)	0.45	4.77 (1.10, 20.55)	0.04	17.10 (0.26, 1.15e+3)	0.32

Table S25 Causality of the risk for PM2.5absorbance in European and Neurosurgical multisystem diseases outcomes (p < 1 × 10-5.).

	Parkinson's disease	0.18 (0.02, 1.37)	0.10	0.20 (0.02, 2.26)	0.19	0.74 (0.01, 46.49)	0.90
	Alzheimer's disease	1.18 (0.48, 2.86)	0.72	1.41 (0.63, 3.13)	0.41	0.48 (0.07, 3.27)	0.53
	Major depressive disorder	0.02 (3.99e-4, 0.57)	0.02	0.01 (1.52e-4, 1.12)	0.06	0.09 (9.42e-9, 8.08e+5)	0.82
	Obsessive Compulsive Disorder	2.63 (7.62e-2, 90.74)	0.59	11.23 (0.44, 2.89e+2)	0.14	0.16 (3.01e-5, 8.21e+2)	0.71
	Stroke	0.89 (0.47, 1.70)	0.72	1.05 (0.50, 2.22)	0.90	1.35 (0.33, 5.61)	0.75
	Intracerebral hemorrhage	2.91 (0.55, 15.53)	0.21	3.02 (0.48, 18.92)	0.24	4.36 (0.15, 1.30e+2)	0.49
	Subarachnoid hemorrhage	2.02 (0.30, 13.80)	0.47	1.55 (0.20, 11.71)	0.67	0.52 (0.01, 35.87)	0.79
	Transient ischemic attack	1.95 (0.37, 8.13)	0.34	1.74 (0.37, 8.13)	0.48	4.49 (0.28, 71.18)	0.48
	Cerebral infarction	1.01 (1.00, 1.03)	0.13	1.01 (0.99, 1.03)	0.29	0.98 (0.95, 1.02)	0.46
	Cerebral aneurysm	1.66 (0.31, 8.87)	0.55	1.36 (0.31, 5.91)	0.68	0.36 (0.01, 17.75)	0.66
	Cervical spondylosis	0.99 (0.97, 1.00)	0.03	0.99 (0.97, 1.00)	0.06	0.99 (0.96, 1.02)	0.45
	Spinal canal stenosis	0.62 (0.19, 1.99)	0.42	0.91 (0.24, 3.47)	0.89	1.64 (0.15, 17.80)	0.72
	spinal meningioma	0.08 (1.07e-6, 5.78e+3)	0.66	0.01 (1.95e-8, 1.97e+3)	0.43	8.89e-4 (1/23e-13, 6.44e+6)	0.65
	Spinal osteochondrosis	0.12 (1.51e-5, 1.01e+3)	0.65	7.91e-2 (3.50e-6, 1.79e+3)	0.62	2.38e-5 (2.82e-13, 2.00e+3)	0.46
	Intracranial and intraspinal abscess	4.66 (6.57e-5, 3.30e+5)	0.79	3.31 (2.45e-5, 4.48e+5)	0.84	3.15 (3.06e-15, 3.24e+3)	0.44
	Cervical spinal cord and nerve injuries	1.28e+2 (3.61e-3, 4.57e+6)	0.36	3.31e+3 (0.45, 2.45e+7)	0.07	1.80e+5 (2.27e-6, 1.42e+16)	0.52
	Glioblastoma	3.98e-3 (4.40e-9, 3.60e+3)	0.43	2.76e-3 (1.13e-9, 6.75e+3)	0.43	1.12e-10 (6.76e-22, 1.87e+1)	0.33
	Benign meningioma	2.39 (0.06, 88.52)	0.64	1.51 (0.02, 92.70)	0.84	0.23 (1.52e-4, 3.36e+2)	0.76

	Malignant meningioma	27.84 (0.11, 7.27e+3)	0.24	10.51 (0.04, 2.47e+3)	0.40	0.03 (2.02e-6, 5.61e+2)	0.62
	Pituitary adenoma and craniopharyngioma	0.26 (1.34e-3, 49.59)	0.61	0.34 (1.88e-3, 59.85)	0.68	1.02 (4.08e-7, 2.54e+6)	0.99
	Benign neoplasm of brain and other parts of CNS	1.49 (0.03, 81.47)	0.84	1.45 (0.02, 1.27e+2)	0.87	1.00 (1.94e-4, 5.19e+3)	0.99
	Malignant neoplasm of brain and other parts of CNS	0.02 (4.82e-8, 5.19e+3)	0.52	4.12e-4 (1.15e-8, 14.83)	0.15	1.31e-6 (2.01e-19, 8.49e+6)	0.53
	Hydrocephalus	12.03 (0.14, 1.02e+3)	0.27	10.90 (0.08, 1.41e+3)	0.34	7.88 (9,87e-4, 6.29e+4)	0.73
	Craniosynostosis	7.29 (0.01, 3.62e+3)	0.53	21.19 (0.03, 1.67e+4)	0.37	1.29e+2 (8.45e-6, 1.98e+9)	0.67
	Concussion	0.64 (0.17, 2.44)	0.51	0.93 (0.24, 3.68)	0.92	2.50 (0.20, 31.98)	0.61
	Diffuse brain injury	0.10 (1.55e-5, 6.38e+2)	0.61	0.02 (8.57e-5, 3.80)	0.14	3.78e-06 (1.79e-10, 0.08)	0.25
	Focal brain injury	0.27 (2.23e-3, 31.96)	0.59	0.14 (1.95e-3, 10.38)	0.37	7.62e-4 (4.09e-7, 1.42)	0.31
	Congenital malformations of the nervous system	1.30 (6.85e-4, 2.46e+3)	0.95	1.66 (2.34e-4, 1.18e+4)	0.91	4.88 (1.70e-8, 1.40e+9)	0.90
Outcome		IVW		Weighted Median		MR-Egger	
		OR (95% CI)	P	OR (95% CI)	P	OR (95% CI)	P
PM2.5absorbance African American or Afro-Caribbean	Trigeminal neuralgia	0.87 (0.63, 1.21)	0.41	0.90 (0.55, 1.48)	0.68	1.05 (0.44, 2.47)	0.92
	Epilepsy	0.89 (0.79, 1.00)	0.045	0.85 (0.71, 1.02)	0.08	0.96 (0.70, 1.31)	0.80
	Parkinson's disease	1.04 (0.88, 1.24)	0.63	1.01 (0.78, 1.31)	0.95	0.89 (0.57, 1.40)	0.62
	Alzheimer's disease	1.02 (0.97,	0.51	1.01 (0.94,	0.77	0.96 (0.86,	0.56

Table S26 Causality of the risk for PM2.5absorbance in African American or Afro-Caribbean and Neurosurgical multisystem diseases outcomes ($p < 1 \times 10^{-5}$.).

		1.07)		1.09)		1.09)	
	Major depressive disorder	0.91 (0.77, 1.06)	0.23	0.86 (0.71, 1.06)	0.15	0.83 (0.51, 1.35)	0.47
	Obsessive Compulsive Disorder	1.00 (0.778, 1.28)	0.99	1.03 (0.73, 1.44)	0.88	1.15 (0.63, 2.12)	0.65
	Stroke	1.03 (0.98, 1.09)	0.26	1.02 (0.94, 1.11)	0.59	1.01 (0.87, 1.17)	0.87
	Intracerebral hemorrhage	15.58	21	0.79	15.84	22	0.82
	Subarachnoid hemorrhage	0.75 (0.61, 0.92)	0.005	0.75 (0.57, 0.99)	0.04	0.79 (0.46, 1.36)	0.41
	Transient ischemic attack	0.96 (0.86, 1.06)	0.41	1.04 (0.89, 1.21)	0.61	1.07 (0.82, 1.42)	0.62
	Cerebral infarction	1.00 (1.00, 1.00)	0.20	1.00 (1.00, 1.00)	0.19	1.00 (1.00, 1.00)	0.42
	Cerebral aneurysm	0.97 (0.82, 1.14)	0.69	0.96 (0.75, 1.23)	0.75	1.11 (0.70, 1.76)	0.67
	Cervical spondylosis	1.00 (1.00, 1.00)	0.58	1.00 (1.00, 1.00)	0.66	1.00 (1.00, 1.00)	0.88
	Spinal canal stenosis	1.01 (0.92, 1.11)	0.76	1.00 (0.87, 1.16)	0.96	1.03 (0.81, 1.30)	0.80
	spinal meningioma	0.71 (0.31, 1.64)	0.42	1.13 (0.30, 4.24)	0.86	2.59 (0.28, 23.85)	0.41
	Spinal osteochondrosis	0.93 (0.47, 1.82)	0.83	0.74 (0.28, 1.93)	0.53	0.60 (0.10, 3.59)	0.58
	Intracranial and intraspinal abscess	0.60 (0.28, 1.30)	0.19	0.47 (0.15, 1.48)	0.20	0.46 (0.06, 3.52)	0.46
	Cervical spinal cord and nerve injuries	0.76 (0.40, 1.42)	0.38	0.58 (0.26, 1.29)	0.18	0.44 (0.08, 2.37)	0.35
	Glioblastoma	0.77 (0.30, 2.00)	0.59	0.91 (0.23, 3.62)	0.89	0.19 (0.02, 2.45)	0.22
	Benign meningioma	0.97 (0.72,1.31)	0.82	0.90 (0.59, 1.38)	0.62	0.95 (0.42, 2.16)	0.91
	Malignant meningioma	1.04 (0.73, 1.50)	0.82	0.99 (0.56, 1.73)	0.96	1.27 (0.49, 3.35)	0.63
	Pituitary adenoma and craniopharyngioma	1.17 (0.84, 1.64)	0.36	0.99 (0.60, 1.63)	0.97	0.80 (0.33, 1.96)	0.63
	Benign neoplasm of brain and other parts of CNS	0.92 (0.67, 1.28)	0.62	0.70 (0.45, 1.09)	0.11	0.64 (0.27, 1.53)	0.33
	Malignant neoplasm of brain and other parts of CNS	1.23 (0.64, 2.37)	0.54	0.85 (0.32, 2.24)	0.74	0.62 (0.11, 3.64)	0.61

	Hydrocephalus	0.87 (0.62, 1.21)	0.41	0.78 (0.48, 1.25)	0.30	0.60 (0.25, 1.48)	0.28
	Craniosynostosis	0.84 (0.53, 1.32)	0.45	0.73 (0.38, 1.41)	0.35	0.87 (0.26, 2.88)	0.82
	Concussion	0.95 (0.87, 1.05)	0.34	0.96 (0.84, 1.09)	0.52	1.20 (0.93, 1.55)	0.17
	Diffuse brain injury	0.66 (0.45, 0.95)	0.03	0.76 (0.46, 1.26)	0.28	1.62 (0.62, 4.19)	0.33
	Focal brain injury	1.10 (0.83, 1.45)	0.52	1.27 (0.86, 1.88)	0.23	1.56 (0.73, 3.29)	0.26
	Congenital malformations of the nervous system	0.90 (0.40, 2.01)	0.79	0.79 (0.32, 1.94)	0.60	0.20 (0.02, 1.60)	0.14

Outcome		IVW		Weighted Median		MR-Egger	
		OR (95% CI)	P	OR (95% CI)	P	OR (95% CI)	P
PM2.5absorbance South Asian	Trigeminal neuralgia	1.02 (0.68, 1.54)	0.91	0.92 (0.52, 1.65)	0.78	0.85 (0.36, 1.99)	0.71
	Epilepsy	0.91 (0.78, 1.07)	0.26	0.92 (0.73, 1.15)	0.46	0.75 (0.53, 1.05)	0.12
	Parkinson's disease	1.08 (0.84, 1.38)	0.57	1.08 (0.79, 1.46)	0.64	1.14 (0.67, 1.93)	0.63
	Alzheimer's disease	1.06 (0.99, 1.13)	0.09	1.06 (0.96, 1.16)	0.25	1.08 (0.95, 1.23)	0.26

Table S27 Causality of the risk for PM2.5absorbance in South Asian and Neurosurgical multisystem diseases outcomes ($p < 1 \times 10-5.$).

	Major depressive disorder	0.79 (0.57, 1.09)	0.15	0.71 (0.46, 1.09)	0.12	1.29 (0.55, 3.06)	0.57
	Obsessive Compulsive Disorder	0.78 (0.57, 1.06)	0.11	0.88 (0.58, 1.34)	0.55	1.01 (0.52, 1.95)	0.99
	Stroke	1.05 (0.97, 1.13)	0.20	1.07 (0.96, 1.18)	0.20	1.13 (0.97. 1.32)	0.15
	Intracerebral hemorrhage	0.97 (0.77, 1.22)	0.80	0.94 (0.69, 1.30)	0.72	0.76 (0.46, 1.25)	0.30
	Subarachnoid hemorrhage	1.02 (0.80, 1.31)	0.87	1.08 (0.76, 1.52)	0.68	1.26 (0.75, 2.13)	0.40
	Transient ischemic attack	1.02 (0.89, 1.18)	0.73	1.02 (0.85, 1.24)	0.78	0.78 (0.59, 1.02)	0.09
	Cerebral infarction	1.00 (1.00, 1.00)	0.28	1.00 (1.00, 1.00)	0.86	1.00 (1.00, 1.00)	0.89
	Cerebral aneurysm	0.82 (0.63, 1.07)	0.14	0.77 (0.53, 1.12)	0.17	1.22 (0.64, 2.33)	0.56
	Cervical spondylosis	1.00 (1.00, 1.00)	0.22	1.00 (1.00, 1.00)	0.77	1.00 (1.00, 1.00)	0.17
	Spinal canal stenosis	0.93 (0.83, 1.05)	0.24	0.94 (0.79, 1.12)	0.51	0.99 (0.77, 1.28)	0.96
	spinal meningioma	1.35 (0.47, 3.89)	0.58	0.90 (0.20, 4.00)	0.89	0.49 (0.05, 4.45)	0.54
	Spinal osteochondrosis	0.77 (0.28, 2.13)	0.62	1.03 (0.29, 3.70)	0.97	0.32 (0.04, 2.69)	0.32
	Intracranial and intraspinal abscess	1.31 (0.50, 3.44)	0.59	1.25 (0.33, 4.75)	0.74	1.47 (0.20, 11.00)	0.71
	Cervical spinal cord and nerve injuries	0.93 (0.43, 1.97)	0.84	0.92 (0.35, 2.42)	0.87	0.53 (0.11, 2.60)	0.45
	Glioblastoma	0.45 (0.13, 1.49)	0.19	0.64 (0.12, 3.51)	0.61	0.12 (0.01, 1.49)	0.12
	Benign meningioma	0.65 (0.46, 0.92)	0.02	0.57 (0.35, 0.93)	0.02	0.53 (0.25, 1.11)	0.12
	Malignant meningioma	1.04 (0.62, 1.76)	0.88	0.78 (0.41, 1.47)	0.44	0.43 (0.16, 1.16)	0.12
	Pituitary adenoma and craniopharyngioma	0.89 (0.58, 1.36)	0.60	1.00 (0.55, 1.83)	0.99	0.90 (0.37, 2.18)	0.82
	Benign neoplasm of brain and other parts of CNS	1.06 (0.73, 1.55)	0.76	0,86 (0.50, 1.46)	0.57	1.81 (0.82, 3.99)	0.17
	Malignant neoplasm of brain and other parts of CNS	1.93 (0.85, 4.37)	0.11	1.15 (0.39, 3.45)	0.80	1.54 (0.28, 8.44)	0.63
	Hydrocephalus	1.64 (1.07, 2.51)	0.02	1.41 (0.81, 2.46)	0.23	1.08 (0.45, 2.62)	0.86

	Craniosynostosis	1.28 (0.72, 2.27)	0.40	1.01 (0.44, 2.31)	0.98	1.27 (0.39, 4.17)	0.70
	Concussion	1.01 (0.90, 1.14)	0.86	0.98 (0.83, 1.15)	0.79	1.19 (0.93, 1.53)	0.19
	Diffuse brain injury	1.76 (1.12, 2.77)	0.01	1.69 (0.90, 3.17)	0.10	1.22 (0.47, 3.12)	0.69
	Focal brain injury	0.83 (0.58, 1.20)	0.33	0.70 (0.43, 1.13)	0.14	0.60 (0.28, 1.27)	0.20
	Congenital malformations of the nervous system	2.05 (1.00, 4.23)	0.05	1.20 (0.44, 3.27)	0.72	0.93 (0.21, 4.19)	0.93

Outcome		IVW		Weighted Median		MR-Egger	
		OR (95% CI)	P	OR (95% CI)	P	OR (95% CI)	P
PM2.5absorbance East Asian	Trigeminal neuralgia	1.06 (0.86, 1.31)	0.56	1.07 (0.82, 1.40)	0.60	0.93 (0.50, 1.72)	0.82
	Epilepsy	1.05 (0.95, 1.15)	0.34	1.00 (0.89, 1.11)	0.94	1.01 (0.75, 1.37)	0.94
	Parkinson's disease	1.04 (0.93, 1.15)	0.49	1.03 (0.89, 1.18)	0.71	1.06 (0.80, 1.41)	0.70
	Alzheimer's disease	0.96 (0.93, 0.99)	0.008	0.96 (0.93, 1.01)	0.09	0.98 (0.91, 1.06)	0.66
	Major depressive disorder	1.01 (0.91, 1.12)	0.86	0.97 (0.85, 1.11)	0.69	0.89 (0.63, 1.25)	0.52

Table S28 Causality of the risk for PM2.5absorbance in East Asian and Neurosurgical multisystem diseases outcomes (p < 1 × 10-5.).

	Obsessive Compulsive Disorder	1.11 (0.95, 1.29)	0.20	1.11 (0.93, 1.33)	0.24	1.30 (0.88, 1.93)	0.22
	Stroke	0.97 (0.93, 1.01)	0.09	0.97 (0.92, 1.01)	0.16	1.00 (0.90, 1.11)	0.97
	Intracerebral hemorrhage	0.96 (0.86, 1.07)	0.48	0.95 (0.82, 1.09)	0.45	0.92 (0.68, 1.25)	0.61
	Subarachnoid hemorrhage	1.00 (0.89, 1.12)	0.96	1.04 (0.88, 1.21)	0.67	1.04 (0.75, 1.44)	0.83
	Transient ischemic attack	0.96 (0.90, 1.02)	0.20	0.95 (0.87, 1.03)	0.22	0.98 (0.81, 1.20)	0.88
	Cerebral infarction	1.00 (1.00, 1.00)	0.41	1.00 (1.00, 1.00)	0.82	1.00 (1.00, 1.00)	0.44
	Cerebral aneurysm	0.94 (0.84, 1.06)	0.31	0.96 (0.82, 1.13)	0.66	1.02 (0.68, 1.52)	0.93
	Cervical spondylosis	1.00 (1.00, 1.00)	0.37	1.00 (1.00, 1.00)	0.47	1.00 (1.00, 1.00)	0.83
	Spinal canal stenosis	0.96 (0.90, 1.03)	0.25	1.00 (0.92, 1.08)	0.91	1.03 (0.86, 1.23)	0.75
	spinal meningioma	1.63 (0.89, 2.98)	0.12	1.57 (0.74, 3.32)	0.24	0.79 (0.13, 4.89)	0.81
	Spinal osteochondrosis	0.81 (0.53, 1.24)	0.33	0.72 (0.40, 1.30)	0.28	1.95 (0.54, 6.99)	0.33
	Intracranial and intraspinal abscess	1.08 (0.66, 1.76)	0.75	0.97 (0.50, 1.89)	0.93	0.46 (0.11, 1.98)	0.33
	Cervical spinal cord and nerve injuries	0.99 (0.69, 1.43)	0.97	0.88 (0.54, 1.45)	0.62	0.46 (0.16, 1.36)	0.19
	Glioblastoma	0.76 (0.41, 1.39)	0.37	0.76 (0.33, 1.77)	0.52	0.52 (0.08, 3.42)	0.51
	Benign meningioma	0.91 (0.76, 1.10)	0.33	0.80 (0.63, 1.03)	0.08	1.05 (0.59, 1.86)	0.88
	Malignant meningioma	1.00 (0.79, 1.26)	0.99	0.99 (0.73, 1.35)	0.97	1.47 (0.74, 2.92)	0.30
	Pituitary adenoma and craniopharyngioma	0.73 (0.59, 0.90)	0.004	0.76 (0.57, 1.01)	0.06	0.76 (0.40, 1.45)	0.43
	Benign neoplasm of brain and other parts of CNS	0.94 (0.74, 1.19)	0.59	0.99 (0.74, 1.32)	0.94	0.96 (0.46, 2.01)	0.92
	Malignant neoplasm of brain and other parts of CNS	1.09 (0.72, 1.65)	0.68	0.91 (0.52, 1.57)	0.72	0.54 (0.16, 1.85)	0.35
	Hydrocephalus	0.96 (0.77, 1.19)	0.71	0.90 (68, 1.19)	0.45	0.82 (0.43, 1.56)	0.56
	Craniosynostosis	0.99 (0.74, 1.32)	0.95	0.97 (0.67, 1.41)	0.87	1.12 (0.47, 2.64)	0.81

	Concussion	1.02 (0.95, 1.09)	0.59	1.00 (0.92, 1.09)	0.98	0.94 (0.77, 1.15)	0.55
	Diffuse brain injury	1.03 (0.82, 1.29)	0.82	1.07 (0.78, 1.48)	0.68	1.89 (0.95, 3.73)	0.10
	Focal brain injury	1.13 (0.91, 1.40)	0.25	1.01 (0.76, 1.32)	0.97	0.76 (0.41, 1.40)	0.40
	Congenital malformations of the nervous system	1.07 (0.67, 1.73)	0.77	0.94 (0.56, 1.57)	0.81	1.90 (0.44, 8.10)	0.41

Table S29 Sensitivity analyses of MR-Egger intercept regression and Cochrane Q tests.

Exposures	Outcomes	Q_MR.Egger	Q_df_MR.Egger	Q_pval	Q_IVW	Q_df_IVW	Q_pval	Egger_intercept	se	pval
NOx European	Trigeminal neuralgia	5.49	4	0.24	5.59	5	0.35	-0.0509	0.1886	0.8005
	Epilepsy	8.39	4	0.08	9.97	5	0.08	-0.0689	0.0794	0.4342
	Parkinson's disease	3.08	4	0.55	3.59	5	0.61	-0.0490	0.0684	0.5139
	Alzheimer's disease	2.37	4	0.67	3.12	5	0.68	-0.0158	0.0183	0.4366
	Major depressive disorder	11.11	4	0.03	14.26	5	0.01	-0.1682	0.1582	0.3475
	Obsessive Compulsive Disorder	2.23	4	0.69	3.51	5	0.62	0.0909	0.08	0.3209
	Stroke	2.71	4	0.61	2.75	5	0.74	0.0041	0.0204	0.8522
	Intracerebral hemorrhage	5.01	4	0.29	6.94	5	0.23	-0.1016	0.0818	0.2819
	Subarachnoid hemorrhage	9.11	4	0.06	15.96	5	0.01	-0.2032	0.1171	0.1578
	Transient ischemic attack	15.56	4	0.00	15.64	5	0.01	-0.0143	0.1030	0.8966
	Cerebral infarction	1.61	4	0.81	3.70	5	0.59	0.0006	0.0004	0.2219
	Cerebral aneurysm	14.52	4	0.01	14.64	5	0.01	0.0286	0.1564	0.8641
	Cervical	4.70	4	0.32	7.6	5	0.17	-0.0006	0.00	0.18

	spondylosis				8				04	64
	Spinal canal stenosis	4.00	4	0.41	4.30	5	0.51	-0.0212	0.0385	0.6107
	spinal meningioma	4.88	4	0.30	5.12	5	0.40	0.2027	0.4585	0.6812
	Spinal osteochondrosis	5.19	4	0.27	5.73	5	0.33	-0.2492	0.3864	0.5540
	Intracranial and intraspinal abscess	2.97	4	0.56	4.40	5	0.49	-0.4606	0.3846	0.2972
	Cervical spinal cord and nerve injuries	2.10	4	0.72	2.28	5	0.81	-0.122	0.2872	0.6925
	Glioblastoma	1.60	4	0.81	1.72	5	0.89	-0.1691	0.47	0.74
	Benign meningioma	2.64	4	0.62	3.16	5	0.67	-0.0984	0.1355	0.5078
	Malignant meningioma	4.91	4	0.30	5.26	5	0.39	-0.1065	0.2001	0.6226
	Pituitary adenoma and craniopharyngioma	5.50	4	0.24	7.22	5	0.20	0.2210	0.1972	0.3253
	Benign neoplasm of brain and other parts of CNS	7.68	4	0.10	7.79	5	0.17	0.0491	0.2088	0.8258
	Malignant neoplasm of brain and other parts of CNS	1.23	4	0.87	1.24	5	0.94	-0.036	0.3213	0.9151
	Hydrocephalus	1.12	4	0.89	1.63	5	0.90	0.1210	0.1690	0.5137
	Craniosynostosis	5.18	4	0.27	5.33	5	0.38	0.0879	0.2584	0.7509
	Concussion	2.89	4	0.58	3.74	5	0.59	-0.0441	0.0477	0.4081
	Diffuse brain injury	5.42	4	0.25	6.40	5	0.27	0.1791	0.2097	0.4412

	Focal brain injury	3.32	4	0.51	3.43	5	0.63	0.0474	0.1421	0.7553
	Congenital malformations of the nervous system	2.69	4	0.61	3.56	5	0.61	0..2667	0.2867	0.4050

Table S30 Sensitivity analyses of MR-Egger intercept regression and Cochrane Q tests.

Exposures	Outcomes	Q_MR.Egger	Q_df_MR.Egger	Q_pval	Q_IVW	Q_df_IVW	Q_pval	Egger_intercept	se	pval
NOx African American or Afro-Caribbean	Trigeminal neuralgia	6.38	15	0.97	6.61	16	0.98	0.0173	0.0361	0.6386
	Epilepsy	16.02	18	0.59	17.20	19	0.58	-0.0134	0.0123	0.2919
	Parkinson's disease	13.93	18	0.73	14.66	19	0.74	-0.0151	0.0176	0.4047
	Alzheimer's disease	19.02	15	0.21	20.96	16	0.18	-0.0071	0.0058	0.2344
	Major depressive disorder	7.23	15	0.95	8.16	16	0.94	-0.0214	0.0222	0.3495
	Obsessive Compulsive Disorder	21.71	14	0.08	22.46	15	0.10	-0.0197	0.0282	0.4975
	Stroke	15.80	18	0.61	18.53	19	0.49	0.0086	0.0052	0.1159
	Intracerebral hemorrhage	15.00	18	0.66	15.78	19	0.67	-0.0152	0.0172	0.3884
	Subarachnoid hemorrhage	22.44	18	0.21	22.86	19	0.24	0.0120	0.0206	0.5667
	Transient ischemic attack	16.38	15	0.36	16.87	16	0.39	0.0081	0.0121	0.5125
	Cerebral infarction	19.62	18	0.35	19.86	19	0.40	-0.0006	0.0001	0.6433
	Cerebral aneurysm	16.70	18	0.54	20.65	19	0.36	-0.0331	0.0166	0.0624

	Cervical spondylosis	15.51	17	0.56	15.55	18	0.62	0.0002	0.0001	0.8410
	Spinal canal stenosis	25.74	18	0.11	25.82	19	0.14	-0.0028	0.0112	0.8161
	spinal meningioma	12.54	15	0.64	12.59	16	0.70	0.0211	0.0933	0.8241
	Spinal osteochondrosis	12.48	15	0.64	13.90	16	0.61	-0.0897	0.0754	0.2528
	Intracranial and intraspinal abscess	19.03	15	0.21	21.18	16	0.17	0.1254	0.0964	0.2131
	Cervical spinal cord and nerve injuries	11.24	15	0.74	13.46	16	0.64	-0.0952	0.0639	0.1569
	Glioblastoma	14.37	15	0.50	14.39	16	0.57	0.0153	0.1062	0.8870
	Benign meningioma	13.69	15	0.55	13.69	16	0.62	0.0002	0.0304	0.9941
	Malignant meningioma	16.80	15	0.33	16.93	16	0.39	0.0145	0.0428	0.7395
	Pituitary adenoma and craniopharyngioma	22.54	15	0.09	22.56	16	0.13	-0.0054	0.0461	0.9090
	Benign neoplasm of brain and other parts of CNS	8.21	15	0.92	8.87	16	0.92	-0.0274	0.0337	0.4287
	Malignant neoplasm of brain and other parts of CNS	12.22	15	0.66	12.57	16	0.70	-0.0429	0.0721	0.5610
	Hydrocephalus	10.78	15	0.77	11.46	16	0.78	-0.0310	0.0376	0.4227
	Craniosynostosis	7.36	15	0.95	7.46	16	0.96	0.0159	0.0505	0.7581
	Concussion	23.18	15	0.08	23.22	16	0.11	0.0021	0.0132	0.8790
	Diffuse brain injury	23.05	15	0.08	28.18	16	0.03	0.0906	0.0496	0.0878
	Focal brain injury	27.23	15	0.03	28.42	16	0.03	-0.0344	0.0426	0.4321
	Congenital malformations of	17.56	15	0.29	17.76	16	0.34	0.0283	0.0692	0.6886

	the nervous system									

Table S31 Sensitivity analyses of MR-Egger intercept regression and Cochrane Q tests.

Exposures	Outcomes	Q_MR.Egger	Q_df_MR.Egger	Q_pval	Q_IVW	Q_df_IVW	Q_pval	Egger_intercept	se	pval
NOx South Asian	Trigeminal neuralgia	9.21	16	0.90	9.91	17	0.91	-0.0287	0.0343	0.4143
	Epilepsy	18.85	18	0.40	19.27	19	0.44	-0.0079	0.0126	0.5375
	Parkinson's disease	9.66	17	0.92	10.96	18	0.90	0.0210	0.0184	0.2700
	Alzheimer's disease	9.37	16	0.90	9.91	17	0.91	0.0045	0.0061	0.4728
	Major depressive disorder	17.05	18	0.52	17.20	19	0.58	0.0093	0.0240	0.7037
	Obsessive Compulsive Disorder	19.20	18	0.38	23.84	19	0.20	-0.0522	0.0251	0.0516
	Stroke	27.27	17	0.05	27.82	18	0.06	0.0043	0.0073	0.5663
	Intracerebral hemorrhage	15.70	18	0.61	18.00	19	0.52	0.0270	0.0178	0.0147
	Subarachnoid hemorrhage	21.20	18	0.27	21.31	19	0.32	-0.0063	0.0207	0.7635
	Transient ischemic attack	18.17	16	0.31	18.77	17	0.34	0.0085	0.0117	0.4795
	Cerebral infarction	12.61	18	0.81	12.64	19	0.86	-0.0002	0.0001	0.8684
	Cerebral aneurysm	14.35	18	0.71	16.68	19	0.61	-0.0274	0.0180	0.1441
	Cervical	15.16	19	0.71	15.	20	0.77	-0.0000	0.00	0.98

	spondylosis				16				01	78
	Spinal canal stenosis	36.28	18	0.01	36.82	19	0.01	-0.0074	0.0143	0.6120
	spinal meningioma	15.68	16	0.48	16.80	17	0.47	-0.0943	0.0892	0.3058
	Spinal osteochondrosis	13.63	16	0.63	16.28	17	0.50	0.1168	0.0717	0.1229
	Intracranial and intraspinal abscess	13.24	16	0.66	13.92	17	0.67	0.0670	0.0813	0.4224
	Cervical spinal cord and nerve injuries	11.34	16	0.79	11.54	17	0.83	0.0277	0.0608	0.6549
	Glioblastoma	11.54	16	0.63	13.80	17	0.68	-0.0523	0.1015	0.6133
	Benign meningioma	14.74	16	0.54	15.09	17	0.59	0.0171	0.0288	0.5620
	Malignant meningioma	18.39	16	0.30	18.76	17	0.34	0.0233	0.0412	0.5798
	Pituitary adenoma and craniopharyngioma	8.66	16	0.93	8.98	17	0.94	-0.0201	0.0358	0.5817
	Benign neoplasm of brain and other parts of CNS	14.79	16	0.54	15.04	17	0.59	-0.0161	0.0320	0.6209
	Malignant neoplasm of brain and other parts of CNS	29.55	16	0.02	29.58	17	0.03	0.0127	0.0935	0.8933
	Hydrocephalus	26.07	16	0.05	26.15	17	0.07	-0.0102	0.0454	0.8256
	Craniosynostosis	12.94	16	0.71	12.51	17	0.77	0.0061	0.0479	0.9005
	Concussion	21.34	16	0.17	21.39	17	0.21	-0.0022	0.0116	0.8516
	Diffuse brain injury	15.30	16	0.50	16.83	17	0.47	-0.0469	0.0379	0.2334

	Focal brain injury	17.23	16	0.37	18.73	17	0.34	-0.0366	0.0310	0.2544
	Congenital malformations of the nervous system	15.94	16	0.46	15.96	17	0.53	0.0077	0.0606	0.9004

Table S32 Sensitivity analyses of MR-Egger intercept regression and Cochrane Q tests.

Exposures	Outcomes	Q_MR.Egger	Q_df_MR.Egger	Q_pval	Q_IVW	Q_df_IVW	Q_pval	Egger_intercept	se	pval
NOx East Asian	Trigeminal neuralgia	6.06	8	0.64	8.82	9	0.45	-0.0741	0.0446	0.1356
	Epilepsy	5.37	9	0.80	6.45	10	0.78	0.0181	0.0174	0.3255
	Parkinson's disease	8.55	9	0.48	11.87	10	0.29	0.0444	0.0244	0.1016
	Alzheimer's disease	4.02	7	0.78	4.03	8	0.85	0.0005	0.0076	0.9454
	Major depressive disorder	6.21	8	0.62	8.01	9	0.53	-0.0291	0.0217	0.2172
	Obsessive Compulsive Disorder	9.21	6	0.16	9.24	7	0.24	-0.0048	0.0394	0.9065
	Stroke	15.61	7	0.03	19.00	8	0.01	-0.0143	0.0116	0.2576
	Intracerebral hemorrhage	5.77	9	0.76	6.15	10	0.80	-0.0158	0.0257	0.5530
	Subarachnoid hemorrhage	14.32	9	0.11	20.86	10	0.02	0.0700	0.0345	0.0733
	Transient ischemic attack	13.57	8	0.09	14.38	9	0.11	-0.0130	0.0187	0.5069
	Cerebral infarction	4.56	9	0.87	5.42	10	0.86	-0.0002	0.0002	0.3788
	Cerebral aneurysm	3.99	9	0.91	4.69	10	0.91	0.0250	0.0298	0.4244
	Cervical	7.01	9	0.64	7.2	10	0.70	0.0008	0.00	0.60

	spondylosis				9				02	91
	Spinal canal stenosis	4.03	9	0.91	4.17	10	0.94	0.0052	0.0134	0.7094
	spinal meningioma	3.51	8	0.90	3.67	9	0.93	0.0462	0.1161	0.7010
	Spinal osteochondrosis	8.94	8	0.35	11.45	9	0.25	0.1473	0.0983	0.1726
	Intracranial and intraspinal abscess	6.92	8	0.55	8.50	9	0.48	-0.1332	0.1057	0.2431
	Cervical spinal cord and nerve injuries	2.68	8	0.95	2.96	9	0.97	-0.0417	0.0790	0.6122
	Glioblastoma	7.60	8	0.47	9.21	9	0.42	-0.1678	0.1322	0.2399
	Benign meningioma	4.96	8	0.76	5.64	9	0.78	0.0310	0.0376	0.4334
	Malignant meningioma	9.10	8	0.33	11.23	9	0.26	0.0731	0.0533	0.2080
	Pituitary adenoma and craniopharyngioma	9.83	8	0.28	10.39	9	0.32	0.0349	0.0516	0.5179
	Benign neoplasm of brain and other parts of CNS	16.25	8	0.04	18.75	9	0.03	0.0659	0.0594	0.2996
	Malignant neoplasm of brain and other parts of CNS	3.69	8	0.88	7.50	9	0.58	0.1753	0.0896	0.0862
	Hydrocephalus	9.64	8	0.29	9.97	9	0.35	0.0269	0.0507	0.6110
	Craniosynostosis	11.59	8	0.17	13.86	9	0.13	-0.0940	0.0751	0.2458
	Concussion	10.66	8	0.22	13.63	9	0.14	0.0225	0.0151	0.1742
	Diffuse brain injury	9.54	8	0.30	10.09	9	0.34	0.0366	0.0538	0.5162

	Focal brain injury	5.19	8	0.74	5.19	9	0.82	0.0024	0.0389	0.9520
	Congenital malformations of the nervous system	9.90	8	0.27	11.26	9	0.26	-0.0918	0.0876	0.3251

Table S33 Sensitivity analyses of MR-Egger intercept regression and Cochrane Q tests.

Exposures	Outcomes	Q_MR.Egger	Q_df_MR.Egger	Q_pval	Q_IVW	Q_df_IVW	Q_pval	Egger_intercept	se	pval
NOx Greater Middle Eastern	Trigeminal neuralgia	20.85	13	0.08	21.26	14	0.10	-0.0282	0.0560	0.6231
	Epilepsy	30.50	17	0.02	30.54	18	0.03	0.0033	0.0231	0.8876
	Parkinson's disease	24.62	17	0.10	25.43	18	0.11	-0.0217	0.0291	0.4665
	Alzheimer's disease	30.71	17	0.02	30.89	18	0.03	-0.0029	0.0090	0.7541
	Major depressive disorder	10.99	12	0.53	11.05	13	0.61	0.0089	0.0379	0.8190
	Obsessive Compulsive Disorder	10.94	10	0.36	11.07	11	0.44	-0.0132	0.0385	0.7385
	Stroke	7.01	13	0.90	7.01	14	0.93	0.0008	0.0082	0.9924
	Intracerebral hemorrhage	20.95	17	0.23	25.92	18	0.10	-0.0565	0.0281	0.0607
	Subarachnoid hemorrhage	20.23	17	0.26	20.69	18	0.30	-0.0182	0.0295	0.5448
	Transient ischemic attack	11.14	13	0.60	11.29	14	0.66	-0.0054	0.0141	0.7104
	Cerebral infarction	17.91	17	0.39	18.02	18	0.45	0.0005	0.0001	0.7459
	Cerebral aneurysm	14.85	17	0.61	15.21	18	0.65	-0.0162	0.0273	0.5594
	Cervical	14.62	17	0.62	15.	18	0.65	0.0001	0.00	0.44

	spondylosis				23				01	47
	Spinal canal stenosis	7.77	17	0.97	10.46	18	0.92	-0.0219	0.0133	0.1192
	spinal meningioma	11.77	13	0.55	11.79	14	0.62	-0.0149	0.1147	0.8988
	Spinal osteochondrosis	15.32	13	0.29	16.17	14	0.30	0.0847	0.1002	0.4135
	Intracranial and intraspinal abscess	22.61	13	0.05	25.87	14	0.03	0.1889	0.1380	0.1943
	Cervical spinal cord and nerve injuries	13.62	13	0.40	13.63	14	0.48	0.0034	0.0803	0.9668
	Glioblastoma	14.57	13	0.34	14.60	14	0.41	-0.0231	0.1386	0.8700
	Benign meningioma	23.90	13	0.03	24.07	14	0.04	0.0154	0.0504	0.7654
	Malignant meningioma	19.79	13	0.10	19.81	14	0.14	0.0068	0.0610	0.9124
	Pituitary adenoma and craniopharyngioma	10.42	13	0.66	10.69	14	0.71	-0.0239	0.0462	0.6137
	Benign neoplasm of brain and other parts of CNS	8.95	13	0.78	12.07	14	0.60	-0.0727	0.0412	0.1009
	Malignant neoplasm of brain and other parts of CNS	11.75	13	0.55	11.75	14	0.63	0.0015	0.8858	0.9865
	Hydrocephalus	10.68	13	0.64	12.18	14	0.59	0.0562	0.0459	0.2420
	Craniosynostosis	22.79	13	0.04	23.89	14	0.05	-0.0646	0.0818	0.4435
	Concussion	10.11	13	0.69	10.64	14	0.71	0.0096	0.0130	0.4763
	Diffuse brain injury	8.62	13	0.80	9.61	14	0.79	-0.0486	0.0489	0.3384

	Focal brain injury	15.03	13	0.31	15.12	14	0.37	0.0113	0.0415	0.7892
	Congenital malformations of the nervous system	9.80	13	0.71	11.66	14	0.63	0.1061	0.0779	0.1962

Table S34 Sensitivity analyses of MR-Egger intercept regression and Cochrane Q tests.

Exposures	Outcomes	Q_MR.Egger	Q_df_MR.Egger	Q_pval	Q_IVW	Q_df_IVW	Q_pval	Egger_intercept	se	pval
NO_2 European	Trigeminal neuralgia	0.93	2	0.63	1.76	3	0.62	0.1457	0.1602	0.4591
	Epilepsy	2.67	2	0.26	5.20	3	0.16	-0.0775	0.0563	0.3025
	Parkinson's disease	9,13	3	0.03	11.75	4	0.02	-0.1285	0.1384	0.4215
	Alzheimer's disease	3.90	2	0.14	5.62	3	0.13	0.0324	0.0345	0.4472
	Major depressive disorder	0.44	2	0.80	7,74	3	0.05	0.2622	0.0970	0.1140
	Obsessive Compulsive Disorder	0.33	2	0.85	0.33	3	0.95	-0.0064	0.1054	0.9571
	Stroke	2.08	2	0.35	3.31	3	0.35	-0.0248	0.0228	0.3904
	Intracerebral hemorrhage	3.19	2	0.20	8.65	3	0.03	0.1584	0.0856	0.2054
	Subarachnoid hemorrhage	5.79	2	0.06	6.01	3	0.11	0.0348	0.1249	0.8066
	Transient ischemic attack	1.05	2	0.59	3.31	3	0.35	-0.0775	0.0516	0.2721
	Cerebral infarction	1.67	2	0.43	6.84	3	0.08	-0.0014	0.0006	0.1508
	Cerebral aneurysm	5.81	2	0.05	6.06	3	0.11	0.0296	0.1007	0.7964
	Cervical	0.85	2	0.65	0.8	3	0.84	-0.0000	0.00	0.99

	spondylosis				5				05	93
	Spinal canal stenosis	0.14	2	0.93	0.18	3	0.98	-0.0087	0.0451	0.8649
	spinal meningioma	0.34	2	0.84	1.11	3	0.78	-0.3604	0.4118	0.4737
	Spinal osteochondrosis	2.66	2	0.26	3.94	3	0.27	-0.3813	0.3883	0.4297
	Intracranial and intraspinal abscess	5.26	2	0.07	6.17	3	0.10	-0.3632	0.6155	0.6149
	Cervical spinal cord and nerve injuries	0.15	2	0.93	0.37	3	0.95	0.1342	0.2845	0.6836
	Glioblastoma	0.87	2	0.65	2.08	3	0.56	0.5136	0.4661	0.3854
	Benign meningioma	2.73	2	0.25	2.90	3	0.41	0.0550	0.1571	0.7598
	Malignant meningioma	7.59	2	0.02	10.07	3	0.02	0.2819	0.3480	0.5030
	Pituitary adenoma and craniopharyngioma	4.99	2	0.08	7.24	3	0.06	-0.2473	0.2602	0.4422
	Benign neoplasm of brain and other parts of CNS	2.08	2	0.35	3.29	3	0.35	0.1639	0.1518	0.3931
	Malignant neoplasm of brain and other parts of CNS	1.57	2	0.46	2.64	3	0.45	-0.3281	0.3175	0.4100
	Hydrocephalus	1.48	2	0.48	1.50	3	0.68	0.0190	0.1672	0.9200
	Craniosynostosis	0.16	2	0.92	0.73	3	0.87	0.1699	0.2244	0.5279
	Concussion	0.59	2	0.74	0.73	3	0.87	0.175	0.0472	0.7466
	Diffuse brain injury	2.27	2	0.32	2.68	3	0.44	0.1132	0.1897	0.6111

	Focal brain injury	12.03	2	0.00	13.21	3	0.00	0.1530	0.3452	0.7010
	Congenital malformations of the nervous system	1.02	2	0.60	1.02	3	0.80	-0.0018	0.2843	0.9956

Table S35 Sensitivity analyses of MR-Egger intercept regression and Cochrane Q tests.

Exposures	Outcomes	Q_MR.Egger	Q_df_MR.Egger	Q_pval	Q_IVW	Q_df_IVW	Q_pval	Egger_intercept	se	pval
NO_2 African American or Afro-Caribbean	Trigeminal neuralgia	15.33	25	0.93	15.33	26	0.95	0.0002	0.0271	0.9956
	Epilepsy	25.71	29	0.64	25.72	30	0.69	0.0011	0.0089	0.9048
	Parkinson's disease	20.98	29	0.86	22.48	30	0.84	0.0170	0.0138	0.2300
	Alzheimer's disease	13.57	27	0.99	14.39	28	0.98	0.0039	0.0044	0.3765
	Major depressive disorder	17.47	20	0.62	18.16	21	0.64	0.0146	0.0176	0.4185
	Obsessive Compulsive Disorder	13.35	20	0.86	14.58	21	0.84	0.0222	0.0200	0.2803
	Stroke	37.43	28	0.11	37.45	29	0.14	0.0005	0.0049	0.9137
	Intracerebral hemorrhage	24.55	29	0.70	24.93	30	0.73	0.0079	0.0127	0.5402
	Subarachnoid hemorrhage	35.64	29	0.18	35.71	30	0.22	0.0035	0.0151	0.8164
	Transient ischemic attack	20.36	25	0.73	20.79	26	0.75	-0.0057	0.0086	0.5155
	Cerebral infarction	39.29	29	0.10	39.32	30	0.12	-0.0000	0.0001	0.8835
	Cerebral aneurysm	22.37	29	0.80	23.79	30	0.78	0.0137	0.0115	0.2426
	Cervical	18.47	28	0.91	18.	29	0.93	0.0000	0.00	0.59

	spondylosis				75				00	75
	Spinal canal stenosis	30.54	29	0.39	30.57	30	0.44	0.0013	0.0080	0.8674
	spinal meningioma	26.51	25	0.38	26.59	26	0.43	0.0199	0.0723	0.7859
	Spinal osteochondrosis	23.87	25	0.53	25.58	26	0.49	0.0736	0.0564	0.2037
	Intracranial and intraspinal abscess	22.98	25	0.58	22.98	26	0.63	-0.0020	0.0641	0.9752
	Cervical spinal cord and nerve injuries	23.13	25	0.57	23.20	26	0.62	-0.0128	0.0479	0.7919
	Glioblastoma	22.65	25	0.60	23.45	26	0.61	-0.0718	0.0803	0.3797
	Benign meningioma	11.63	25	0.99	12.15	26	0.99	-0.0164	0.0227	0.4764
	Malignant meningioma	19.80	25	0.76	19.96	26	0.79	-0.0119	0.0302	0.6965
	Pituitary adenoma and craniopharyngioma	22.60	25	0.61	23.16	26	0.62	-0.0212	0.0284	0.4610
	Benign neoplasm of brain and other parts of CNS	26.86	25	0.36	29.86	26	0.27	-0.0437	0.0261	0.1067
	Malignant neoplasm of brain and other parts of CNS	23.77	25	0.53	24.07	26	0.57	0.0292	0.0543	0.5951
	Hydrocephalus	19.38	25	0.78	20.98	26	0.74	-0.0352	0.0279	0.2185
	Craniosynostosis	28.84	25	0.27	29.30	26	0.30	0.0257	0.0405	0.5320
	Concussion	21.14	25	0.69	23.83	26	0.59	-0.0131	0.0080	0.1131
	Diffuse brain injury	16.99	25	0.88	17.51	26	0.89	0.0214	0.0299	0.4812

	Focal brain injury	16.64	25	0.89	16.96	26	0.91	-0.0134	0.0236	0.5759
	Congenital malformations of the nervous system	25.89	25	0.41	28.39	26	0.34	0.0754	0.0485	0.1326

Table S36 Sensitivity analyses of MR-Egger intercept regression and Cochrane Q tests.

Exposures	Outcomes	Q_MR.Egger	Q_df_MR.Egger	Q_pval	Q_IVW	Q_df_IVW	Q_pval	Egger_intercept	se	pval
NO_2 South Asian	Trigeminal neuralgia	16.82	18	0.54	17.35	19	0.57	-0.0311	0.0423	0.4726
	Epilepsy	15.00	21	0.82	15.12	22	0.86	-0.0055	0.0157	0.7323
	Parkinson's disease	21.23	21	0.45	24.61	22	0.32	-0.0402	0.0220	0.0817
	Alzheimer's disease	24.83	20	0.21	27.25	21	0.16	-0.0105	0.0075	0.1788
	Major depressive disorder	20.29	17	0.26	20.74	18	0.29	0.0192	0.0314	0.5495
	Obsessive Compulsive Disorder	13.89	14	0.46	14.51	15	0.49	0.0252	0.0321	0.4459
	Stroke	12.10	16	0.74	12.41	17	0.77	0.0041	0.0073	0.5857
	Intracerebral hemorrhage	19.85	20	0.47	19.88	21	0.53	-0.0040	0.0232	0.8636
	Subarachnoid hemorrhage	16.87	21	0.72	17.17	22	0.75	-0.0133	0.0239	0.5850
	Transient ischemic attack	18.09	18	0.45	21.29	19	0.32	0.0244	0.0137	0.0911
	Cerebral infarction	10.17	20	0.96	10.76	21	0.97	0.0001	0.0002	0.4518
	Cerebral aneurysm	10.95	21	0.9	10.96	22	0.98	-0.0018	0.0241	0.9423
	Cervical	31.39	20	0.05	32.	21	0.05	0.0001	0.00	0.39

	spondylosis				55				02	93
	Spinal canal stenosis	22.31	21	0.38	30.18	22	0.11	0.0341	0.0125	0.0128
	spinal meningioma	10.36	18	0.92	10.38	19	0.94	0.0184	0.1102	0.8693
	Spinal osteochondrosis	20.43	18	0.31	20.43	19	0.37	-0.0000	0.0943	0.9994
	Intracranial and intraspinal abscess	20.93	18	0.28	21.71	19	0.30	-0.0884	0.1084	0.4256
	Cervical spinal cord and nerve injuries	18.84	18	0.40	20.26	19	0.38	0.0896	0.0768	0.2588
	Glioblastoma	14.08	18	0.72	14.33	19	0.76	0.0626	0.1248	0.6218
	Benign meningioma	12.56	18	0.82	12.68	19	0.85	0.0124	0.0357	0.7330
	Malignant meningioma	17.04	18	0.52	17.05	19	0.59	-0.0050	0.0475	0.9168
	Pituitary adenoma and craniopharyngioma	8.43	18	0.97	8.50	19	0.98	0.0116	0.0440	0.7949
	Benign neoplasm of brain and other parts of CNS	18.71	18	0.41	19.03	19	0.45	-0.0224	0.0404	0.5862
	Malignant neoplasm of brain and other parts of CNS	22.11	18	0.23	22.14	19	0.28	-0.0143	0.0942	0.8810
	Hydrocephalus	19.87	18	0.34	21.89	19	0.29	-0.0628	0.0464	0.1927
	Craniosynostosis	15.68	18	0.61	18.65	19	0.48	-0.1026	0.0596	0.1021
	Concussion	16.07	18	0.59	18.31	19	0.50	-0.0187	0.0125	0.1518
	Diffuse brain injury	27.86	18	0.06	28.51	19	0.07	0.0379	0.0585	0.5247

	Focal brain injury	22.05	18	0.23	23.23	19	0.23	0.0403	0.0411	0.3395
	Congenital malformations of the nervous system	19.70	18	0.35	20.48	19	0.37	-0.0663	0.0788	0.4107

Table S37 Sensitivity analyses of MR-Egger intercept regression and Cochrane Q tests.

Exposures	Outcomes	Q_MR.Egger	Q_df_MR.Egger	Q_pval	Q_IVW	Q_df_IVW	Q_pval	Egger_intercept	se	pval
NO_2 East Asian	Trigeminal neuralgia	5.65	9	0.77	5.65	10	0.84	0.0035	0.0415	0.9355
	Epilepsy	7.02	12	0.86	7.22	13	0.89	0.0064	0.0143	0.6648
	Parkinson's disease	12.99	12	0.37	13.30	13	0.42	0.0106	0.0197	0.6022
	Alzheimer's disease	8.95	9	0.44	10.03	10	0.44	-0.0065	0.0063	0.3254
	Major depressive disorder	4.67	9	0.86	5.85	10	0.83	0.0181	0.0166	0.3048
	Obsessive Compulsive Disorder	5.77	7	0.57	6.54	8	0.59	0.0248	0.0282	0.4078
	Stroke	9.14	10	0.52	9.24	11	0.60	0.0022	0.0069	0.7603
	Intracerebral hemorrhage	15.18	12	0.23	16.28	13	0.23	-0.0204	0.0219	0.3692
	Subarachnoid hemorrhage	12.85	12	0.38	12.85	13	0.46	0.0010	0.0214	0.9640
	Transient ischemic attack	5.21	9	0.82	5.65	10	0.84	0.0088	0.0133	0.5240
	Cerebral infarction	10.62	12	0.56	10.67	13	0.64	-0.0000	0.0001	0.8351
	Cerebral aneurysm	16.82	12	0.16	16.82	13	0.21	-0.0012	0.0261	0.9650
	Cervical	8.53	12	0.74	10.	13	0.64	0.0002	0.00	0.16

	spondylosis				70				01	60
	Spinal canal stenosis	10.61	12	0.56	11.31	13	0.59	0.0089	0.0107	0.4196
	spinal meningioma	13.05	9	0.16	13.29	10	0.21	0.0532	0.1295	0.6910
	Spinal osteochondrosis	7.83	9	0.55	7.89	10	0.64	-0.0204	0.0863	0.8183
	Intracranial and intraspinal abscess	11.63	9	0.23	11.76	10	0.30	0.0345	0.1117	0.7647
	Cervical spinal cord and nerve injuries	6.57	9	0.68	7.22	10	0.70	0.0589	0.0733	0.4425
	Glioblastoma	8.68	9	0.47	8.71	10	0.56	0.0204	0.1223	0.8712
	Benign meningioma	8.34	9	0.50	9.82	10	0.46	-0.0425	0.0350	0.2553
	Malignant meningioma	10.47	9	0.31	14.47	10	0.15	-0.0931	0.0502	0.0968
	Pituitary adenoma and craniopharyngioma	12.07	9	0.21	12.47	10	0.25	0.0273	0.0501	0.5986
	Benign neoplasm of brain and other parts of CNS	8.77	9	0.46	8.98	10	0.53	0.0176	0.0387	0.6599
	Malignant neoplasm of brain and other parts of CNS	17.40	9	0.04	17.95	10	0.06	0.0616	0.1156	0.6071
	Hydrocephalus	8.18	9	0.52	8.21	10	0.61	0.0070	0.0431	0.8739
	Craniosynostosis	12.08	9	0.21	13.42	10	0.20	0.0673	0.0673	0.3437
	Concussion	4.26	9	0.89	5.42	10	0.86	0.0132	0.0122	0.3082
	Diffuse brain injury	5.81	9	0.76	5.83	10	0.83	0.0053	0.0459	0.9101

	Focal brain injury	8.54	9	0.48	8.68	10	0.56	0.0135	0.0362	0.7174
	Congenital malformations of the nervous system	10.16	9	0.34	10.25	10	0.42	-0.0233	0.0783	0.7728

Table S38 Sensitivity analyses of MR-Egger intercept regression and Cochrane Q tests.

Exposures	Outcomes	Q_MR.Egger	Q_df_MR.Egger	Q_pval	Q_IVW	Q_df_IVW	Q_pval	Egger_intercept	se	pval
NO_2 Greater Middle Eastern and Ne	Trigeminal neuralgia	20.77	18	0.29	21.49	19	0.31	-0.0336	0.0426	0.4398
	Epilepsy	24.20	22	0.34	24.58	23	0.37	-0.0095	0.0161	0.5602
	Parkinson's disease	18.38	22	0.68	20.29	23	0.62	-0.0280	0.0203	0.1801
	Alzheimer's disease	18.77	20	0.54	22.42	21	0.38	-0.0117	0.0061	0.0707
	Major depressive disorder	17.97	20	0.59	18.11	21	0.64	0.0114	0.0306	0.7127
	Obsessive Compulsive Disorder	13.49	16	0.64	14.07	17	0.66	0.0232	0.0306	0.4579
	Stroke	12.39	16	0.72	13.11	17	0.73	-0.0066	0.0078	0.4071
	Intracerebral hemorrhage	14.91	22	0.87	20.38	23	0.62	-0.0515	0.0220	0.0289
	Subarachnoid hemorrhage	28.49	22	0.16	28.83	23	0.19	0.0136	0.0266	0.6129
	Transient ischemic attack	12.06	18	0.84	13.10	19	0.83	-0.0130	0.0127	0.3196
	Cerebral infarction	23.72	22	0.36	24.73	23	0.36	-0.0001	0.0001	0.3436
	Cerebral aneurysm	21.28	22	0.50	25.66	23	0.32	-0.0547	0.0261	0.0480
	Cervical	21.42	19	0.31	24.	20	0.21	0.0002	0.00	0.10

	spondylosis				66				01	63
	Spinal canal stenosis	17.14	22	0.76	17.71	23	0.77	-0.0085	0.0112	0.4576
	spinal meningioma	14.55	18	0.69	14.84	19	0.73	0.0545	0.1027	0.6020
	Spinal osteochondrosis	11.99	18	0.85	12.16	19	0.88	-0.0341	0.0826	0.6849
	Intracranial and intraspinal abscess	20.15	18	0.32	20.16	19	0.38	0.0113	0.0994	0.9109
	Cervical spinal cord and nerve injuries	31.70	18	0.02	31.92	19	0.03	-0.0336	0.0935	0.7233
	Glioblastoma	8.28	18	0.97	8.40	19	0.98	-0.0396	0.1168	0.7387
	Benign meningioma	26.58	18	0.09	26.83	19	0.11	-0.0167	0.0405	0.6863
	Malignant meningioma	14.35	18	0.71	17.39	19	0.56	-0.077	0.044	0.098
	Pituitary adenoma and craniopharyngioma	22.46	18	0.21	26.52	19	0.12	-0.0833	0.0461	0.0878
	Benign neoplasm of brain and other parts of CNS	17.89	18	0.46	18.52	19	0.49	0.0295	0.0370	0.4352
	Malignant neoplasm of brain and other parts of CNS	18.56	18	0.42	24.40	19	0.18	0.1918	0.0806	0.0285
	Hydrocephalus	20.41	18	0.31	21.01	19	0.34	0.0318	0.0438	0.4769
	Craniosynostosis	11.35	18	0.88	11.42	19	0.91	-0.0151	0.0554	0.7886
	Concussion	10.45	18	0.92	11.23	19	0.92	-0.010	0.0116	0.3876
	Diffuse brain injury	11.15	18	0.89	16.88	19	0.60	-0.1051	0.0439	0.0278

	Focal brain injury	15.56	18	0.62	15.70	19	0.68	-0.0132	0.0346	0.7070
	Congenital malformations of the nervous system	18.19	18	0.44	20.17	19	0.38	0.0986	0.0704	0.1783

Table S39 Sensitivity analyses of MR-Egger intercept regression and Cochrane Q tests.

Exposures	Outcomes	Q_MR.Egger	Q_df_MR.Egger	Q_pval	Q_IVW	Q_df_IVW	Q_pval	Egger_intercept	se	pval
PM2.5 European	Trigeminal neuralgia	5.05	4	0.28	5.07	5	0.41	0.0050	0.0429	0.9130
	Epilepsy	16.42	4	0.00	116.46	5	0.01	-0.0024	0.0266	0.9316
	Parkinson's disease	4.20	4	0.38	4.55	5	0.47	-0.0116	0.0203	0.5983
	Alzheimer's disease	4.38	4	0.36	4.49	5	0.48	0.0028	0.0087	0.7634
	Major depressive disorder	26.02	3	0.00	27.04	4	0.00	0.0638	0.1853	0.7534
	Obsessive Compulsive Disorder	3.00	3	0.39	3.01	4	0.56	-0.0033	0.0293	0.9169
	Stroke	6.11	4	0.19	8.36	5	0.14	0.0090	0.0075	0.2920
	Intracerebral hemorrhage	2.64	4	0.62	2.68	5	0.75	0.0040	0.0188	0.8416
	Subarachnoid hemorrhage	9.90	4	0.04	10.21	5	0.07	-0.0112	0.0317	0.7419
	Transient ischemic attack	5.26	4	0.26	5.75	5	0.33	0.0086	0.0140	0.5729
	Cerebral infarction	2.92	3	0.40	3.38	4	0.50	0.0002	0.0004	0.5486
	Cerebral aneurysm	5.44	4	0.24	5.91	5	0.32	0.0127	0.0217	0.5898
	Cervical	9.79	5	0.08	12.	6	0.05	-0.0004	0.00	0.29

	spondylosis				45				04	61
	Spinal canal stenosis	8.81	4	0.07	11.29	5	0.05	0.0175	0.0165	0.3488
	spinal meningioma	6.57	4	0.16	6.59	5	0.25	-0.0146	0.1271	0.9143
	Spinal osteochondrosis	2.91	4	0.57	3.37	5	0.64	0.0542	0.0799	0.5350
	Intracranial and intraspinal abscess	4.41	4	0.35	7.62	5	0.18	0.1630	0.0954	0.1627
	Cervical spinal cord and nerve injuries	2.00	4	0.74	2.89	5	0.72	0.0635	0.0673	0.3992
	Glioblastoma	1.32	4	0.86	1.36	5	0.93	0.0206	0.1122	0.8634
	Benign meningioma	2.74	4	0.60	2.86	5	0.72	-0.0111	0.0320	0.7460
	Malignant meningioma	4.74	4	0.32	6.11	5	0.30	0.0501	0.0465	0.3420
	Pituitary adenoma and craniopharyngioma	6.02	4	0.20	8.75	5	0.12	-0.0656	0.0487	0.2491
	Benign neoplasm of brain and other parts of CNS	5.51	4	0.24	10.02	5	0.07	-0.0756	0.0418	0.1447
	Malignant neoplasm of brain and other parts of CNS	1.20	4	0.88	3.50	5	0.62	-0.1159	0.0764	0.2037
	Hydrocephalus	0.08	4	1.00	2.46	5	0.78	0.0609	0.0394	0.1976
	Craniosynostosis	1.23	4	0.87	2.03	5	0.84	0.0479	0.0534	0.4209
	Concussion	5.62	4	0.23	5.64	5	0.34	-0.0016	0.0133	0.9090
	Diffuse brain injury	12.60	4	0.01	12.89	5	0.02	-0.0225	0.0745	0.7781

	Focal brain injury	1.94	4	0.75	2.29	5	0.81	0.0198	0.0331	0.5823
	Congenital malformations of the nervous system	3.50	4	0.48	3.50	5	0.62	-0.0029	0.0673	0.9675

Table S40 Sensitivity analyses of MR-Egger intercept regression and Cochrane Q tests.

Exposures	Outcomes	Q_MR.Egger	Q_df_MR.Egger	Q_pval	Q_IVW	Q_df_IVW	Q_pval	Egger_intercept	se	pval
PM2.5 African American or Afro-Caribbean	Trigeminal neuralgia	20.58	21	0.48	21.04	22	0.52	-0.0252	0.0374	0.5077
	Epilepsy	20.18	22	0.57	20.39	23	0.62	-0.0061	0.0134	0.6543
	Parkinson's disease	24.14	22	0.34	24.97	23	0.35	0.0170	0.0195	0.3937
	Alzheimer's disease	15.80	21	0.78	16.79	22	0.77	0.0055	0.0055	0.3307
	Major depressive disorder	24.32	21	0.28	24.50	22	0.32	0.0076	0.0190	0.6953
	Obsessive Compulsive Disorder	26.54	20	0.15	26.95	21	0.17	-0.0148	0.0266	0.5834
	Stroke	27.16	21	0.17	27.32	22	0.20	0.0023	0.0064	0.7259
	Intracerebral hemorrhage	16.56	22	0.79	16.68	23	0.82	0.0066	0.0189	0.7313
	Subarachnoid hemorrhage	28.53	22	0.16	28.62	23	0.19	-0.0061	0.0230	0.7916
	Transient ischemic attack	18.33	21	0.63	18.60	22	0.67	-0.0062	0.0120	0.6095
	Cerebral infarction	18.78	22	0.66	18.79	23	0.71	0.0000	0.0001	0.9361
	Cerebral aneurysm	20.13	22	0.57	20.36	23	0.62	-0.0093	0.0195	0.6364
	Cervical	31.24	22	0.09	31.	23	0.11	0.0000	0.00	0.69

	spondylosis				47				01	13
	Spinal canal stenosis	20.49	22	0.55	20.66	23	0.60	-0.0043	0.0104	0.6827
	spinal meningioma	17.60	21	0.67	19.47	22	0.62	-0.1322	0.0967	0.1859
	Spinal osteochondrosis	21.00	21	0.46	21.09	22	0.52	0.0233	0.0778	0.7678
	Intracranial and intraspinal abscess	17.02	21	0.71	17.23	22	0.75	0.0413	0.0887	0.6466
	Cervical spinal cord and nerve injuries	24.99	21	0.25	25.45	22	0.28	0.0450	0.0721	0.5390
	Glioblastoma	15.08	21	0.82	16.58	22	0.79	0.1351	0.1103	0.2339
	Benign meningioma	26.23	21	0.20	26.24	22	0.24	-0.0031	0.0352	0.9303
	Malignant meningioma	18.72	21	0.60	19.05	22	0.64	-0.0241	0.0419	0.5715
	Pituitary adenoma and craniopharyngioma	11.86	21	0.94	12.52	22	0.95	0.0316	0.0390	0.4267
	Benign neoplasm of brain and other parts of CNS	24.39	21	0.27	25.03	22	0.30	0.0280	0.0376	0.4646
	Malignant neoplasm of brain and other parts of CNS	26.10	21	0.20	26.24	22	0.24	0.0277	0.0834	07428
	Hydrocephalus	19.55	21	0.55	20.29	22	0.56	0.0335	0.0388	0.3985
	Craniosynostosis	14.65	21	0.84	14.78	22	0.87	0.0185	0.0523	0.7269
	Concussion	11.68	21	0.95	16.43	22	0.79	-0.0239	0.0110	0.0408
	Diffuse brain injury	19.83	21	0.53	23.20	22	0.39	-0.0759	0.0414	0.0805

	Focal brain injury	17.18	21	0.70	17.89	22	0.71	-0.0274	0.0326	0.4099
	Congenital malformations of the nervous system	38.36	21	0.01	42.06	22	0.01	0.1273	0.0894	0.1692

Table S41 Sensitivity analyses of MR-Egger intercept regression and Cochrane Q tests.

Exposures	Outcomes	Q_MR.Egger	Q_df_MR.Egger	Q_pval	Q_IVW	Q_df_IVW	Q_pval	Egger_intercept	se	pval
PM2.5 South Asian	Trigeminal neuralgia	20.66	24	0.66	20.71	25	0.71	0.0068	0.0283	0.8127
	Epilepsy	20.32	26	0.78	21.43	27	0.77	-0.0110	0.0105	0.3013
	Parkinson's disease	27.53	26	0.38	30.46	27	0.29	0.0241	0.0145	0.1083
	Alzheimer's disease	51.44	23	0.00	51.44	24	0.00	0.0001	0.0064	0.9841
	Major depressive disorder	19.10	23	0.70	19.14	24	0.74	-0.0037	0.0184	0.8406
	Obsessive Compulsive Disorder	26.97	21	0.17	30.47	22	0.11	0.0394	0.0238	0.1133
	Stroke	28.92	24	0.22	29.48	25	0.24	0.0033	0.0049	0.5030
	Intracerebral hemorrhage	19.45	26	0.82	19.59	27	0.85	0.0055	0.0148	0.7107
	Subarachnoid hemorrhage	39.86	26	0.04	39.93	27	0.05	0.0039	0.0195	0.8412
	Transient ischemic attack	35.99	24	0.06	38.26	25	0.04	-0.0137	0.0111	0.2302
	Cerebral infarction	24.48	26	0.55	24.52	27	0.60	0.0000	0.0000	0.8463
	Cerebral aneurysm	19.39	26	0.82	19.41	27	0.85	0.0027	0.0167	0.8744
	Cervical	35.49	26	0.10	36.	27	0.10	-0.0000	0.00	0.31

	spondylosis				93				00	38
	Spinal canal stenosis	42.97	26	0.02	43.94	27	0.02	0.0077	0.0100	0.4513
	spinal meningioma	19.89	24	0.70	19.93	25	0.75	-0.0146	0.0732	0.8435
	Spinal osteochondrosis	27.89	24	0.26	28.92	25	0.27	0.0599	0.0636	0.3552
	Intracranial and intraspinal abscess	20.16	24	0.69	23.61	25	0.54	0.1243	0.0660	0.0757
	Cervical spinal cord and nerve injuries	23.28	24	0.50	23.90	25	0.53	0.0393	0.0500	0.4389
	Glioblastoma	31.34	24	0.14	32.61	25	0.14	-0.0939	0.0952	0.3339
	Benign meningioma	30.90	24	0.16	34.89	25	0.09	0.0476	0.0270	0.0908
	Malignant meningioma	24.12	24	0.45	25.28	25	0.45	0.0342	0.0318	0.2931
	Pituitary adenoma and craniopharyngioma	20.16	24	0.69	22.03	25	0.63	0.0403	0.0295	0.1850
	Benign neoplasm of brain and other parts of CNS	16.72	24	0.86	16.91	25	0.89	0.0113	0.0264	0.6710
	Malignant neoplasm of brain and other parts of CNS	16.91	24	0.85	21.98	25	0.64	0.1273	0.0565	0.0338
	Hydrocephalus	37.84	24	0.04	41.48	25	0.02	-0.0560	0.0369	0.1420
	Craniosynostosis	26.72	24	0.32	26.72	25	0.37	-0.003	0.042	0.953
	Concussion	11.69	24	0.98	11.71	25	0.99	0.0010	0.0083	0.9058
	Diffuse brain injury	23.69	24	0.48	23.84	25	0.53	-0.0119	0.0313	0.7074

	Focal brain injury	22.71	24	0.54	26.54	25	0.38	-0.0484	0.0247	0.0622
	Congenital malformations of the nervous system	19.82	24	0.71	23.26	25	0.56	-0.0929	0.0501	0.0760

Table S42 Sensitivity analyses of MR-Egger intercept regression and Cochrane Q tests.

Exposures	Outcomes	Q_MR.Egger	Q_df_MR.Egger	Q_pval	Q_IVW	Q_df_IVW	Q_pval	Egger_intercept	se	pval
PM2.5 East Asian	Trigeminal neuralgia	7.53	10	0.68	8.01	11	0.71	-0.0293	0.0422	0.5027
	Epilepsy	10.16	11	0.52	11.72	12	0.47	0.0186	0.0149	0.2384
	Parkinson's disease	5.64	11	0.90	6.21	12	0.91	0.0157	0.0207	0.4651
	Alzheimer's disease	6.49	10	0.77	6.51	11	0.84	0.0009	0.0066	0.8886
	Major depressive disorder	9.91	10	0.45	11.81	11	0.38	0.0259	0.0188	0.1977
	Obsessive Compulsive Disorder	5.21	8	0.73	6.50	9	0.69	-0.0342	0.0302	0.2894
	Stroke	5.41	10	0.86	5.42	11	0.91	-0.0005	0.0066	0.9314
	Intracerebral hemorrhage	14.04	11	0.23	15.64	12	0.21	-0.0263	0.0235	0.2868
	Subarachnoid hemorrhage	11.50	11	0.40	11.98	12	0.45	0.0153	0.0226	0.5120
	Transient ischemic attack	16.15	10	0.10	16.86	11	0.11	0.0113	0.0172	0.5236
	Cerebral infarction	7.39	11	0.77	9.48	12	0.66	-0.0002	0.0001	0.1766
	Cerebral aneurysm	10.13	11	0.52	10.61	12	0.56	-0.0155	0.0225	0.5056
	Cervical	11.42	11	0.41	13.	12	0.34	0.0002	0.00	0.19

	spondylosis				37			01	87
	Spinal canal stenosis	8.44	11	0.67	8.47	12	0.75	0.0019	0.0116	0.8711
	spinal meningioma	8.95	10	0.54	11.92	11	0.37	0.1883	0.1093	0.1156
	Spinal osteochondrosis	6.37	11	0.85	7.17	12	0.85	0.0745	0.0831	0.3890
	Intracranial and intraspinal abscess	17.48	10	0.06	17.67	11	0.09	0.0435	0.1321	0.7490
	Cervical spinal cord and nerve injuries	7.01	10	0.72	7.35	11	0.77	0.0434	0.0746	0.5737
	Glioblastoma	5.41	10	0.86	6.21	11	0.86	-0.1114	0.1248	0.3930
	Benign meningioma	5.61	10	0.85	5.64	11	0.90	0.0058	0.0354	0.8738
	Malignant meningioma	6.69	10	0.75	8.05	11	0.71	0.0552	0.0472	0.2695
	Pituitary adenoma and craniopharyngioma	6.90	10	0.74	7.78	11	0.73	-0.0414	0.0441	0.3692
	Benign neoplasm of brain and other parts of CNS	7.41	10	0.69	8.35	11	0.68	0.0380	0.0393	0.3562
	Malignant neoplasm of brain and other parts of CNS	4.05	10	0.95	7.38	11	0.77	0.1541	0.0845	0.0981
	Hydrocephalus	10.52	10	0.40	10.52	11	0.48	0.0021	0.0448	0.9634
	Craniosynostosis	16.06	10	0.10	16.18	11	0.13	-0.0197	0.0747	0.7969
	Concussion	17.11	10	0.07	17.11	11	0.10	-0.0000	0.0162	0.9983
	Diffuse brain injury	9.70	10	0.47	11.66	11	0.39	0.0653	0.0466	0.1917

	Focal brain injury	8.52	10	0.58	8.61	11	0.66	-0.0110	0.0367	0.7707
	Congenital malformations of the nervous system	10.32	10	0.41	11.67	11	0.39	0.0865	0.0755	0.2785

Table S43 Sensitivity analyses of MR-Egger intercept regression and Cochrane Q tests.

Exposures	Outcomes	Q_MR.Egger	Q_df_MR.Egger	Q_pval	Q_IVW	Q_df_IVW	Q_pval	Egger_intercept	se	pval
PM2.5 Greater Middle Eastern	Trigeminal neuralgia	22.06	26	0.69	25.73	27	0.53	-0.0725	0.0378	0.0663
	Epilepsy	36.46	29	0.16	38.07	30	0.15	-0.0183	0.0162	0.2680
	Parkinson's disease	25.75	29	0.64	27.15	30	0.62	-0.0246	0.0208	0.2462
	Alzheimer's disease	31.06	27	0.27	32.17	28	0.27	0.0071	0.0072	0.3340
	Major depressive disorder	23.79	22	0.36	25.13	23	0.34	0.0449	0.0404	0.2776
	Obsessive Compulsive Disorder	16.77	19	0.61	17.28	20	0.64	-0.0278	0.0391	0.4858
	Stroke	33.98	24	0.08	39.54	25	0.03	0.0178	0.0090	0.0592
	Intracerebral hemorrhage	24.02	29	0.73	24.26	30	0.76	-0.0103	0.0214	0.6324
	Subarachnoid hemorrhage	41.36	29	0.06	45.99	30	0.03	0.0492	0.0273	0.0820
	Transient ischemic attack	31.69	26	0.20	32.04	27	0.23	-0.0071	0.0133	0.5972
	Cerebral infarction	28.72	29	0.48	28.75	30	0.53	-0.0000	0.0002	0.8597
	Cerebral aneurysm	18.38	29	0.94	18.41	30	0.95	-0.0037	0.0228	0.8728
	Cervical	35.09	28	0.17	39.	29	0.09	0.0003	0.00	0.07

	spondylosis				45				02	28
	Spinal canal stenosis	38.83	29	0.10	39.14	30	0.12	-0.0064	0.0132	0.6306
	spinal meningioma	11.49	26	0.99	11.79	27	0.99	-0.0534	0.0982	0.5915
	Spinal osteochondrosis	17.96	26	0.88	20.32	27	0.82	-0.1213	0.0790	0.1369
	Intracranial and intraspinal abscess	26.85	26	0.42	27.56	27	0.43	-0.0759	0.0910	0.4117
	Cervical spinal cord and nerve injuries	33.82	26	0.14	33.85	27	0.17	0.0114	0.0762	0.8827
	Glioblastoma	19.28	26	0.82	20.68	27	0.80	0.1321	0.1117	0.2478
	Benign meningioma	20.45	26	0.77	20.76	27	0.80	-0.0178	0.0318	0.5802
	Malignant meningioma	16.85	26	0.91	18.24	27	0.90	-0.0500	0.0424	0.2488
	Pituitary adenoma and craniopharyngioma	22.00	26	0.69	22.02	27	0.74	0.0042	0.0394	0.9150
	Benign neoplasm of brain and other parts of CNS	24.21	26	0.56	24.32	27	0.61	-0.0120	0.0352	0.7357
	Malignant neoplasm of brain and other parts of CNS	27.00	26	0.41	27.37	27	0.44	0.0463	0.0773	0.5546
	Hydrocephalus	36.07	26	0.09	36.07	27	0.11	-0.0023	0.0460	0.9597
	Craniosynostosis	26.90	26	0.41	28.47	27	0.39	-0.0659	0.0535	0.2289
	Concussion	15.63	26	0.94	15.91	27	0.95	-0.0058	0.0110	0.6040
	Diffuse brain injury	29.14	26	0.30	29.14	27	0.35	-0.0016	0.0441	0.9711

	Focal brain injury	37.27	26	0.07	38.31	27	0.07	0.0334	0.0393	0.4023
	Congenital malformations of the nervous system	20.45	26	0.77	20.46	27	0.81	0.0062	0.0663	0.9265

Table S44 Sensitivity analyses of MR-Egger intercept regression and Cochrane Q tests.

Exposures	Outcomes	Q_MR.Egger	Q_df_MR.Egger	Q_pval	Q_IVW	Q_df_IVW	Q_pval	Egger_intercept	se	pval
PM2.5－10 European	Trigeminal neuralgia	27.47	36	0.85	29.19	37	0.82	0.0252	0.0192	0.1987
	Epilepsy	48.91	39	0.13	48.91	40	0.16	0.0003	0.0068	0.9670
	Parkinson's disease	51.83	39	0.08	51.87	40	0.10	0.0020	0.0119	0.8642
	Alzheimer's disease	28.18	37	0.85	28.20	38	0.88	-0.0006	0.0045	0.8889
	Major depressive disorder	31.67	32	0.48	33.53	33	0.44	-0.0396	0.0290	0.1818
	Obsessive Compulsive Disorder	23.75	31	0.82	23.89	32	0.85	-0.0086	0.0230	0.7111
	Stroke	40.44	30	0.10	41.46	31	0.10	-0.0063	0.0072	0.3910
	Intracerebral hemorrhage	39.42	39	0.45	41.15	40	0.42	-0.0114	0.0087	0.1988
	Subarachnoid hemorrhage	39.77	39	0.44	39.80	40	0.48	0.0017	0.0094	0.8589
	Transient ischemic attack	42.19	36	0.22	43.96	37	0.20	-0.0082	0.0067	0.2269
	Cerebral infarction	28.33	38	0.87	28.90	39	0.88	-0.0009	0.0001	0.4547
	Cerebral aneurysm	36.16	39	0.60	37.96	40	0.56	0.0104	0.0077	0.1880
	Cervical	51.77	37	0.05	53.	38	0.05	0.0001	0.00	0.32

	spondylosis				18				01	19
	Spinal canal stenosis	30.76	39	0.82	31.76	40	0.82	-0.0059	0.0059	0.3206
	spinal meningioma	39.60	36	0.31	40.05	37	0.34	0.0340	0.0527	0.5229
	Spinal osteochondrosis	20.28	36	0.98	20.28	37	0.99	-0.0002	0.0403	0.9960
	Intracranial and intraspinal abscess	23.21	36	0.95	23.34	37	0.96	-0.0160	0.0456	0.7277
	Cervical spinal cord and nerve injuries	35.47	36	0.49	36.82	37	0.48	-0.0398	0.0342	0.2521
	Glioblastoma	31.85	36	0.67	32.43	37	0.68	0.0434	0.0569	0.4506
	Benign meningioma	37.69	36	0.39	39.42	37	0.36	-0.0213	0.0166	0.2068
	Malignant meningioma	27.72	36	0.84	29.12	37	0.82	-0.0255	0.0216	0.2453
	Pituitary adenoma and craniopharyngioma	35.16	36	0.51	35.91	37	0.52	0.0173	0.0200	0.3924
	Benign neoplasm of brain and other parts of CNS	40.40	36	0.28	40.40	37	0.32	-0.0012	0.0190	0.9498
	Malignant neoplasm of brain and other parts of CNS	22.50	36	0.96	22.51	37	0.97	0.0050	0.0387	0.8974
	Hydrocephalus	32.60	36	0.63	32.65	37	0.67	-0.0046	0.0199	0.8173
	Craniosynostosis	44.44	36	0.16	45.30	37	0.16	0.0249	0.0299	0.4107
	Concussion	40.60	36	0.27	40.63	37	0.31	0.0010	0.0060	0.8707
	Diffuse brain injury	27.45	36	0.85	32.13	37	0.70	-0.0460	0.0213	0.0371

	Focal brain injury	50.41	36	0.06	50.51	37	0.07	0.0053	0.0199	0.7910
	Congenital malformations of the nervous system	48.18	36	0.08	48.18	37	0.10	-0.0008	0.0393	0.9842

Table S45 Sensitivity analyses of MR-Egger intercept regression and Cochrane Q tests.

Exposures	Outcomes	Q_MR.Egger	Q_df_MR.Egger	Q_pval	Q_IVW	Q_df_IVW	Q_pval	Egger_intercept	se	pval
PM2.5 – 10 African American or Afro-Caribbean	Trigeminal neuralgia	11.29	17	0.84	11.29	18	0.88	-0.0039	0.0581	0.9467
	Epilepsy	14.18	19	0.77	15.38	20	0.75	-0.0231	0.0211	0.2878
	Parkinson's disease	21.17	19	0.33	22.11	20	0.33	-0.0293	0.0319	0.3697
	Alzheimer's disease	9.88	17	0.91	9.90	18	0.94	-0.0013	0.0098	0.8965
	Major depressive disorder	11.42	15	0.72	11.88	16	0.75	0.0264	0.0388	0.5074
	Obsessive Compulsive Disorder	11.95	15	0.68	12.08	16	0.74	-0.0161	0.0445	0.7224
	Stroke	12.79	17	0.75	15.81	18	0.61	0.0160	0.0092	0.1003
	Intracerebral hemorrhage	27.57	19	0.09	28.74	20	0.09	-0.0328	0.0364	0.3795
	Subarachnoid hemorrhage	22.35	19	0.27	23.25	20	0.28	-0.0307	0.0350	0.3919
	Transient ischemic attack	24.84	17	0.10	25.08	18	0.12	-0.0091	0.0225	0.6921
	Cerebral infarction	7.67	19	0.99	7.78	20	0.99	0.0007	0.0002	0.7434
	Cerebral aneurysm	14.70	19	0.74	16.69	20	0.67	-0.0430	0.0305	0.1747
	Cervical	12.62	19	0.86	13.	20	0.87	-0.0001	0.00	0.49

	spondylosis				10				02	56
	Spinal canal stenosis	18.71	19	0.48	20.95	20	0.40	-0.0252	0.0168	0.1514
	spinal meningioma	9.76	17	0.91	10.72	18	0.91	-0.1484	0.1511	0.3398
	Spinal osteochondrosis	8.90	17	0.94	9.51	18	0.95	-0.095	0.1214	0.4446
	Intracranial and intraspinal abscess	12.87	17	0.74	12.92	18	0.80	0.0305	0.1380	0.8277
	Cervical spinal cord and nerve injuries	12.77	17	0.75	15.55	18	0.62	0.1718	0.1030	0.1138
	Glioblastoma	15.72	17	0.54	18.39	18	0.43	-0.2816	0.1722	0.1203
	Benign meningioma	17.87	17	0.40	20.92	18	0.28	-0.0853	0.0501	0.1067
	Malignant meningioma	11.88	17	0.81	14.01	18	0.73	-0.0951	0.0651	0.1622
	Pituitary adenoma and craniopharyngioma	14.80	17	0.61	15.25	18	0.64	-0.0407	0.0607	0.5115
	Benign neoplasm of brain and other parts of CNS	9.90	17	0.91	10.50	18	0.91	-0.0420	0.0542	0.4491
	Malignant neoplasm of brain and other parts of CNS	20.43	17	0.25	22.32	18	0.22	0.1603	0.1279	0.2270
	Hydrocephalus	17.66	17	0.41	17.68	18	0.48	0.0082	0.0614	0.8947
	Craniosynostosis	7.91	17	0.97	8.15	18	0.98	-0.0403	0.0812	0.6265
	Concussion	15.96	17	0.53	18.08	18	0.45	0.0249	0.0171	0.1633
	Diffuse brain injury	18.47	17	0.36	19.27	18	0.38	0.0577	0.0671	0.4017

	Focal brain injury	13.61	17	0.69	16.74	18	0.54	0.0897	0.0507	0.0948
	Congenital malformations of the nervous system	13.89	17	0.67	13.93	18	0.73	-0.0203	0.1025	0.8451

Table S46 Sensitivity analyses of MR-Egger intercept regression and Cochrane Q tests.

Exposures	Outcomes	Q_MR.Egger	Q_df_MR.Egger	Q_pval	Q_IVW	Q_df_IVW	Q_pval	Egger_intercept	se	pval
PM2.5－10 South Asian	Trigeminal neuralgia	10.77	10	0.38	10.81	11	0.46	0.0118	0.0591	0.8460
	Epilepsy	8.47	12	0.75	9,84	13	0.71	-0.0232	0.0198	0.2640
	Parkinson's disease	8.44	12	0.75	8.46	13	0.81	-0.0039	0.0283	0.8927
	Alzheimer's disease	13.10	11	0.29	13.11	12	0.36	-0.0012	0.0094	0.9028
	Major depressive disorder	5.53	6	0.48	5.66	7	0.58	-0.0152	0.0426	0.7335
	Obsessive Compulsive Disorder	8.19	7	0.32	9.41	8	0.31	0.0434	0.0425	0.3414
	Stroke	8.28	8	0.41	8.30	9	0.50	0.0015	0.0098	0.8780
	Intracerebral hemorrhage	15.43	12	0.22	15.72	13	0.26	-0.0152	0.0315	0.6386
	Subarachnoid hemorrhage	8.98	12	0.70	9.78	13	0.71	-0.0264	0.0296	0.3903
	Transient ischemic attack	3.77	10	0.96	4.07	11	0.97	0.0101	0.0183	0.5926
	Cerebral infarction	7.52	12	0.82	7.94	13	0.85	0.0001	0.0002	0.5302
	Cerebral aneurysm	11.61	12	0.48	12.24	13	0.51	0.0230	0.0292	0.4451
	Cervical	11.61	12	0.48	12.	13	0.51	0.0230	0.02	0.44

	spondylosis				24				92	51
	Spinal canal stenosis	18.84	12	0.09	19.59	13	0.11	0.0138	0.0199	0.5014
	spinal meningioma	5.08	10	0.89	5.32	11	0.91	-0.0726	0.1482	0.6347
	Spinal osteochondrosis	15.81	10	0.11	16.48	11	0.12	0.0971	0.1494	0.5307
	Intracranial and intraspinal abscess	4.83	10	0.90	8.04	11	0.71	0.2417	0.1350	0.1037
	Cervical spinal cord and nerve injuries	6.11	10	0.81	6.12	11	0.87	0.0073	0.1009	0.9441
	Glioblastoma	5.06	10	0.89	5.06	11	0.93	0.0073	0.1684	0.9661
	Benign meningioma	17.92	10	0.06	18.69	11	0.07	0.0422	0.0640	0.5248
	Malignant meningioma	4.91	10	0.90	5.79	11	0.89	0.0599	0.0638	0.3696
	Pituitary adenoma and craniopharyngioma	11.17	10	0.34	11.18	11	0.43	0.0066	0.0628	0.9190
	Benign neoplasm of brain and other parts of CNS	10.32	10	0.41	10.72	11	0.47	-0.0339	0.0540	0.5445
	Malignant neoplasm of brain and other parts of CNS	7.22	10	0.70	8.41	11	0.68	-0.1245	0.1143	0.3017
	Hydrocephalus	9.22	10	0.51	9.80	11	0.55	0.0448	0.0590	0.4654
	Craniosynostosis	3.61	10	0.96	3.70	11	0.98	-0.0229	0.0798	0.7804
	Concussion	8.22	10	0.61	8.60	11	0.66	-0.0103	0.0167	0.5509
	Diffuse brain injury	13.08	10	0.22	13.84	11	0.24	0.0550	0.0721	0.4633

	Focal brain injury	20.14	10	0.03	20.22	11	0.04	-0.0136	0.0705	0.8511
	Congenital malformations of the nervous system	9.25	10	0.51	9.25	11	0.60	-0.0006	0.1007	0.9955

Table S47 Sensitivity analyses of MR-Egger intercept regression and Cochrane Q tests.

Exposures	Outcomes	Q_MR.Egger	Q_df_MR.Egger	Q_pval	Q_IVW	Q_df_IVW	Q_pval	Egger_intercept	se	pval
PM2.5－10 East Asian	Trigeminal neuralgia	4.93	7	0.67	5.22	8	0.73	0.0189	0.0351	0.6069
	Epilepsy	9.44	6	0.15	9.51	7	0.22	-0.0037	0.0174	0.8398
	Parkinson's disease	3.55	6	0.74	4.63	7	0.71	-0.0210	0.0202	0.3385
	Alzheimer's disease	2.02	6	0.92	4.36	7	0.74	-0.0094	0.0061	0.1769
	Major depressive disorder	3.73	6	0.71	4.14	7	0.76	-0.0111	0.0174	0.5474
	Obsessive Compulsive Disorder	2.95	4	0.57	3.55	5	0.62	0.0243	0.0314	0.4823
	Stroke	5.79	5	0.33	5.81	6	0.44	0.0011	0.0072	0.8866
	Intracerebral hemorrhage	5.00	7	0.66	7.21	8	0.51	0.0281	0.0189	0.1805
	Subarachnoid hemorrhage	2.96	7	0.89	3.34	8	0.91	0.0124	0.0202	0.5578
	Transient ischemic attack	5.80	7	0.56	5.87	8	0.66	-0.0029	0.0112	0.8058
	Cerebral infarction	6.49	7	0.48	6.71	8	0.57	0.0007	0.0001	0.6480
	Cerebral aneurysm	4.73	7	0.69	8.55	8	0.38	-0.0400	0.0204	0.0915
	Cervical	7.37	7	0.39	7.7	8	0.46	-0.0007	0.00	0.58

	spondylosis				2				01	18
	Spinal canal stenosis	1.98	6	0.92	3.11	7	0.87	-0.0120	0.0113	0.3293
	spinal meningioma	6.50	7	0.48	9.37	8	0.31	0.1544	0.0910	0.1337
	Spinal osteochondrosis	9.83	7	0.20	9.84	8	0.28	-0.0085	0.0870	0.9247
	Intracranial and intraspinal abscess	6.43	7	0.49	6.74	8	0.57	-0.0460	0.0832	0.5972
	Cervical spinal cord and nerve injuries	6.71	7	0.46	7.64	8	0.47	0.0597	0.0621	0.3690
	Glioblastoma	4.76	7	0.69	5.11	8	0.75	-0.0610	0.1037	0.5749
	Benign meningioma	12.14	7	0.10	18.21	8	0.02	0.0727	0.0388	0.1034
	Malignant meningioma	13.15	7	0.07	14.56	8	0.07	0.0467	0.0538	0.4143
	Pituitary adenoma and craniopharyngioma	10.26	7	0.17	10.78	8	0.21	-0.0264	0.0443	0.5699
	Benign neoplasm of brain and other parts of CNS	10.14	7	0.18	11.53	8	0.17	0.0386	0.0394	0.3601
	Malignant neoplasm of brain and other parts of CNS	9.87	7	0.20	11.85	8	0.16	0.0990	0.0835	0.2744
	Hydrocephalus	6.50	7	0.48	8.97	8	0.34	-0.0572	0.0364	0.1596
	Craniosynostosis	1.04	7	0.99	1.07	8	0.99	-0.0084	0.0490	0.8686
	Concussion	6.29	7	0.51	6.62	8	0.58	0.0059	0.0103	0.5846
	Diffuse brain injury	3.91	7	0.79	3.93	8	0.86	-0.0059	0.0388	0.8841

	Focal brain injury	6.99	7	0.43	7.27	8	0.51	-0.0162	0.0306	0.6123
	Congenital malformations of the nervous system	4.75	7	0.69	4.77	8	0.78	0.0075	0.0619	0.9064

Table S48 Sensitivity analyses of MR-Egger intercept regression and Cochrane Q tests.

Exposures	Outcomes	Q_MR.Egger	Q_df_MR.Egger	Q_pval	Q_IVW	Q_df_IVW	Q_pval	Egger_intercept	se	pval
PM2.5－10 Greater Middle Eastern	Trigeminal neuralgia	16.70	23	0.82	16.70	24	0.86	-0.0023	0.0291	0.9389
	Epilepsy	35.45	25	0.08	35.91	26	0.09	-0.0071	0.0124	0.5710
	Parkinson's disease	30.30	25	0.21	30.47	26	0.25	0.0066	0.0173	0.7086
	Alzheimer's disease	39.70	23	0.02	39.70	24	0.02	-0.0005	0.0068	0.9948
	Major depressive disorder	16.22	15	0.37	17.25	16	0.37	-0.0402	0.0412	0.3453
	Obsessive Compulsive Disorder	16.05	15	0.38	16.61	16	0.41	0.0276	0.0381	0.4803
	Stroke	16.73	21	0.73	20.81	22	0.53	0.0129	0.0064	0.0562
	Intracerebral hemorrhage	18.49	25	0.82	21.53	26	0.71	0.0261	0.0150	0.0932
	Subarachnoid hemorrhage	20.84	25	0.70	21.92	26	0.69	-0.0167	0.0161	0.3093
	Transient ischemic attack	24.50	23	0.38	25.15	24	0.40	-0.0076	0.0096	0.4409
	Cerebral infarction	31.20	23	0.12	35.02	24	0.07	-0.0002	0.0001	0.1068
	Cerebral aneurysm	22.81	25	0.59	23.51	26	0.60	-0.0119	0.0142	0.4127
	Cervical	20.35	23	0.62	20.	24	0.65	0.0007	0.00	0.52

	spondylosis				78				01	10
	Spinal canal stenosis	24.00	25	0.52	24.94	26	0.52	0.0085	0.0088	0.3407
	spinal meningioma	24.65	23	0.37	27.27	24	0.29	0.1227	0.0784	0.1313
	Spinal osteochondrosis	19.24	23	0.69	19.28	24	0.74	-0.0120	0.0610	0.8459
	Intracranial and intraspinal abscess	16.97	23	0.81	16.98	24	0.85	0.0028	0.0691	0.9675
	Cervical spinal cord and nerve injuries	26.12	23	0.30	27.05	24	0.30	0.0498	0.0550	0.3746
	Glioblastoma	29.96	23	0.15	29.96	24	0.19	-0.0041	0.0984	0.9675
	Benign meningioma	22.34	23	0.50	24.13	24	0.45	0.0327	0.0245	0.1943
	Malignant meningioma	23.02	23	0.46	23.03	24	0.52	0.0031	0.0326	0.9244
	Pituitary adenoma and craniopharyngioma	22.62	23	0.48	23.23	24	0.51	0.0238	0.0304	0.4408
	Benign neoplasm of brain and other parts of CNS	34.83	23	0.05	37.87	24	0.04	-0.0474	0.0334	0.1697
	Malignant neoplasm of brain and other parts of CNS	24.16	23	0.39	24.34	24	0.44	-0.0249	0.0599	0.6809
	Hydrocephalus	10.30	23	0.99	10.33	24	0.99	0.0049	0.0302	0.8719
	Craniosynostosis	23.82	23	0.41	23.85	24	0.47	0.0069	0.0414	0.8694
	Concussion	41.60	23	0.01	41.69	24	0.01	0.0026	0.0115	0.8218
	Diffuse brain injury	28.79	23	0.19	28.85	24	0.23	-0.0075	0.0361	0.8376

	Focal brain injury	34.69	23	0.06	35.40	24	0.06	0.0213	0.0312	0.5008
	Congenital malformations of the nervous system	16.89	23	0.81	17.58	24	0.82	-0.0430	0.0515	0.4128

Table S49 Sensitivity analyses of MR-Egger intercept regression and Cochrane Q tests.

Exposures	Outcomes	Q_MR.Egger	Q_df_MR.Egger	Q_pval	Q_IVW	Q_df_IVW	Q_pval	Egger_intercept	se	pval
PM10 European	Trigeminal neuralgia	20.20	18	0.32	20.25	19	0.38	0.0100	0.0488	0.8402
	Epilepsy	27.98	18	0.06	28.00	19	0.08	-0.0022	0.0216	0.9205
	Parkinson's disease	23.07	18	0.19	23.69	19	0.21	0.0193	0.0279	0.4977
	Alzheimer's disease	39.21	18	0.00	45.18	19	0.00	0.0183	0.0110	0.1151
	Major depressive disorder	4.95	14	0.99	4.98	15	0.99	-0.0051	0.0341	0.8839
	Obsessive Compulsive Disorder	8.38	15	0.91	8.40	16	0.94	0.0053	0.0349	0.8813
	Stroke	25.75	16	0.06	26.52	17	0.07	0.0075	0.0108	0.4979
	Intracerebral hemorrhage	7.99	18	0.98	8.14	19	0.99	0.0097	0.0253	0.7053
	Subarachnoid hemorrhage	21.28	18	0.27	22.35	19	0.27	0.0280	0.0294	0.3545
	Transient ischemic attack	12.28	18	0.83	14.47	19	0.76	-0.0219	0.0148	0.1562
	Cerebral infarction	26.08	18	0.10	26.47	19	0.12	0.0001	0.0002	0.6096
	Cerebral aneurysm	16.20	18	0.58	16.63	19	0.61	-0.0176	0.0268	0.5195
	Cervical	28.49	18	0.05	28.	19	0.07	0.0009	0.00	0.66

	spondylosis				81				02	00
	Spinal canal stenosis	16.29	18	0.57	16.63	19	0.61	0.0080	0.0137	0.5650
	spinal meningioma	17.10	18	0.52	17.11	19	0.58	-0.0055	0.1199	0.9638
	Spinal osteochondrosis	14.04	18	0.73	14.30	19	0.77	-0.0494	0.0967	0.6159
	Intracranial and intraspinal abscess	20.60	18	0.30	21.54	19	0.31	0.1065	0.1172	0.3757
	Cervical spinal cord and nerve injuries	17.16	18	0.51	17.17	19	0.58	-0.0077	0.0818	0.9265
	Glioblastoma	13.77	18	0.74	14.43	19	0.76	0.1105	0.1357	0.4260
	Benign meningioma	24.78	18	0.13	26.35	19	0.12	0.0486	0.0454	0.2987
	Malignant meningioma	19.76	18	0.35	21.95	19	0.29	0.0764	0.0541	0.1747
	Pituitary adenoma and craniopharyngioma	14.15	18	0.72	14.17	19	0.77	0.0069	0.0480	0.8871
	Benign neoplasm of brain and other parts of CNS	14.27	18	0.71	15.33	19	0.70	-0.0444	0.0431	0.3164
	Malignant neoplasm of brain and other parts of CNS	17.38	18	0.50	18.10	19	0.52	-0.0785	0.0926	0.4078
	Hydrocephalus	13.17	18	0.78	14.80	19	0.74	0.0611	0.0478	0.2175
	Craniosynostosis	19.12	18	0.38	19.33	19	0.44	-0.0297	0.0667	0.6617
	Concussion	24.20	18	0.15	24.66	19	0.17	-0.0092	0.0157	0.5671
	Diffuse brain injury	20.57	18	0.30	22.02	19	0.28	0.0611	0.0544	0.2757

	Focal brain injury	25.69	18	0.11	25.75	19	0.14	0.0104	0.0480	0.8311
	Congenital malformations of the nervous system	19.26	18	0.38	19.26	19	0.44	-0.0011	0.0843	0.9897

Table S50 Sensitivity analyses of MR-Egger intercept regression and Cochrane Q tests.

Exposures	Outcomes	Q_MR.Egger	Q_df_MR.Egger	Q_pval	Q_IVW	Q_df_IVW	Q_pval	Egger_intercept	se	pval
PM10 African American or Afro-Caribbean	Trigeminal neuralgia	13.51	11	0.26	13.52	12	0.33	0.0059	0.0701	0.9345
	Epilepsy	11.09	14	0.68	11.19	15	0.74	0.0065	0.0208	0.7597
	Parkinson's disease	8.15	14	0.88	11.02	15	0.75	-0.0531	0.0313	0.1122
	Alzheimer's disease	7.90	12	0.79	13.83	13	0.39	-0.0238	0.0098	0.0315
	Major depressive disorder	15.16	14	0.37	15.99	15	0.38	-0.0297	0.0340	0.3962
	Obsessive Compulsive Disorder	9.22	12	0.68	9.41	13	0.74	0.0188	0.0436	0.6735
	Stroke	4.59	12	0.97	5.02	13	0.97	-0.0062	0.0094	0.5252
	Intracerebral hemorrhage	21.67	14	0.09	22.18	15	0.10	0.0209	0.0363	0.5736
	Subarachnoid hemorrhage	8.42	14	0.87	8.58	15	0.90	-0.0124	0.0313	0.6970
	Transient ischemic attack	12.03	11	0.36	14.22	12	0.29	-0.0298	0.0211	0.1846
	Cerebral infarction	11.19	14	0.67	11.19	15	0.74	0.0000	0.0002	0.9965
	Cerebral aneurysm	21.81	14	0.08	21.94	15	0.11	0.0096	0.0333	0.7769
	Cervical	22.65	14	0.07	22.	15	0.09	0.0001	0.00	0.96

	spondylosis				65				03	49
	Spinal canal stenosis	13.83	14	0.46	13.84	15	0.54	-0.0017	0.0177	0.9268
	spinal meningioma	6.57	11	0.83	6.58	12	0.88	-0.0099	0.1637	0.9528
	Spinal osteochondrosis	26.66	11	0.01	26.71	12	0.01	-0.0293	0.2058	0.8892
	Intracranial and intraspinal abscess	12.18	11	0.35	13.43	12	0.34	0.1678	0.1575	0.3094
	Cervical spinal cord and nerve injuries	15.19	11	0.17	15.79	12	0.20	0.0866	0.1314	0.5232
	Glioblastoma	11.72	11	0.38	12.11	12	0.44	0.1161	0.1938	0.5611
	Benign meningioma	7.13	11	0.79	9.49	12	0.66	-0.0813	0.0530	0.1534
	Malignant meningioma	12.01	11	0.36	12.12	12	0.44	-0.0237	0.0739	0.7542
	Pituitary adenoma and craniopharyngioma	12.33	11	0.34	13.21	12	0.35	0.0622	0.0702	0.3947
	Benign neoplasm of brain and other parts of CNS	4.78	11	0.94	5.08	12	0.96	-0.0320	0.0589	0.5976
	Malignant neoplasm of brain and other parts of CNS	15.45	11	0.16	15.68	12	0.21	0.0609	0.1504	0.6935
	Hydrocephalus	4.90	11	0.94	5.49	12	0.94	-0.0501	0.0652	0.4583
	Craniosynostosis	20.16	11	0.04	21.78	12	0.04	0.1123	0.1192	0.3663
	Concussion	4.25	11	0.96	4.42	12	0.97	-0.0076	0.0186	0.6926
	Diffuse brain injury	14.58	11	0.20	14.81	12	0.25	-0.0336	0.0806	0.6851

	Focal brain injury	10.38	11	0.50	10.42	12	0.58	-0.0115	0.0552	0.8387
	Congenital malformations of the nervous system	10.11	11	0.52	12.52	12	0.40	0.1727	0.1112	0.1488

Table S51 Sensitivity analyses of MR-Egger intercept regression and Cochrane Q tests.

Exposures	Outcomes	Q_MR.Egger	Q_df_MR.Egger	Q_pval	Q_IVW	Q_df_IVW	Q_pval	Egger_intercept	se	pval
PM10 East Asian	Trigeminal neuralgia	2.59	7	0.92	2.60	8	0.96	0.0042	0.0446	0.9281
	Epilepsy	7.49	8	0.48	8.03	9	0.53	0.0124	0.0168	0.4833
	Parkinson's disease	6.75	8	0.56	8.28	9	0.51	-0.0300	0.0242	0.2515
	Alzheimer's disease	15.75	7	0.03	15.84	8	0.04	-0.0023	0.0115	0.8469
	Major depressive disorder	12.76	7	0.08	12.76	8	0.12	0.0009	0.0445	0.9837
	Obsessive Compulsive Disorder	8.25	5	0.14	8.25	6	0.22	0.0005	0.0522	0.9927
	Stroke	7.79	7	0.35	12.29	8	0.14	-0.0177	0.0088	0.0842
	Intracerebral hemorrhage	5.02	8	0.75	5.06	9	0.83	-0.0047	0.0250	0.8569
	Subarachnoid hemorrhage	5.67	7	0.58	5.71	8	0.68	-0.0062	0.0287	0.8341
	Transient ischemic attack	3.61	7	0.82	6.32	8	0.61	0.0235	0.0143	0.1441
	Cerebral infarction	8.72	8	0.37	8.75	9	0.46	-0.0003	0.0002	0.8650
	Cerebral aneurysm	3.78	8	0.88	3.82	9	0.92	0.0060	0.0293	0.8419
	Cervical	3.18	8	0.92	4.3	9	0.89	-0.0002	0.00	0.31

	spondylosis				0				002	94
	Spinal canal stenosis	1.09	8	0.99	1.2 1	9	0.99	-0.0047	0.01 34	0.73 30
	spinal meningioma	8.01	7	0.33	12. 21	8	0.14	0.2372	0.12 38	0.09 70
	Spinal osteochondro sis	5.10	7	0.65	5.5 0	8	0.70	0.0588	0.09 21	0.54 78
	Intracranial and intraspinal abscess	8.61	7	0.28	9.5 7	8	0.30	0.1034	0.11 72	0.40 67
	Cervical spinal cord and nerve injuries	4.88	7	0.67	5.9 6	8	0.65	0.0819	0.07 90	0.33 446
	Glioblastoma	11.47	7	0.12	13. 57	8	0.09	0.1906	0.16 84	0.29 50
	Benign meningioma	1.62	7	0.98	2.1 0	8	0.98	0.0260	0.03 75	0.51 10
	Malignant meningioma	3.45	7	0.84	4.6 1	8	0.80	0.0536	0.04 99	0.31 79
	Pituitary adenoma and craniopharyn gioma	5.49	7	0.60	5.8 0	8	0.67	-0.0263	0.04 65	0.58 96
	Benign neoplasm of brain and other parts of CNS	7.96	7	0.34	8.7 5	8	0.36	0.0369	0.04 44	0.43 30
	Malignant neoplasm of brain and other parts of CNS	9.11	7	0.24	9.1 4	8	0.33	-0.0135	0.10 19	0.89 82
	Hydrocephalu s	4.83	7	0.68	7.2 1	8	0.51	0.0713	0.04 62	0.16 69
	Craniosynost osis	3.47	7	0.84	3.5 6	8	0.89	0.0188	0.06 23	0.77 16
	Concussion	1.02	7	0.99	3.5 0	8	0.90	-0.0206	0.01 31	0.15 95
	Diffuse brain injury	8.73	7	0.27	13. 32	8	0.10	0.1055	0.05 50	0.09 67

	Focal brain injury	4.74	7	0.69	6.67	8	0.57	0.0540	0.0388	0.2075
	Congenital malformations of the nervous system	17.61	7	0.01	17.68	8	0.02	0.0205	0.1250	0.8744

Table S52 Sensitivity analyses of MR-Egger intercept regression and Cochrane Q tests.

Exposures	Outcomes	Q_MR.Egger	Q_df_MR.Egger	Q_pval	Q_IVW	Q_df_IVW	Q_pval	Egger_intercept	se	pval
PM10 Greater Middle Eastern	Trigeminal neuralgia	6.17	14	0.96	6.18	15	0.98	-0.0012	0.0343	0.9734
	Epilepsy	13.65	14	0.48	13.85	15	0.54	0.0060	0.0135	0.6644
	Parkinson's disease	14.17	14	0.44	14.22	15	0.51	0.0043	0.0182	0.8176
	Alzheimer's disease	17.73	14	0.22	19.47	15	0.19	-0.0071	0.0060	0.2615
	Major depressive disorder	14.60	10	0.15	15.48	11	0.16	-0.0405	0.0523	0.4566
	Obsessive Compulsive Disorder	11.53	11	0.40	11.56	12	0.48	-0.0074	0.0427	0.8648
	Stroke	16.49	13	0.22	17.30	14	0.24	0.0061	0.0076	0.4388
	Intracerebral hemorrhage	11.97	14	0.61	11.97	15	0.68	-0.0003	0.0193	0.9878
	Subarachnoid hemorrhage	17.83	14	0.21	19.90	15	0.18	-0.0295	0.0232	0.2233
	Transient ischemic attack	13.61	14	0.48	14.34	15	0.50	0.0095	0.0111	0.4064
	Cerebral infarction	21.41	15	0.12	21.86	16	0.15	-0.0008	0.0001	0.5820
	Cerebral aneurysm	6.93	14	0.94	8.28	15	0.91	-0.0261	0.0226	0.2658
	Cervical	20.17	15	0.17	20.	16	0.20	-0.0006	0.00	0.64

	spondylosis				47				01	35
	Spinal canal stenosis	11.55	14	0.64	14.85	15	0.46	0.0182	0.0100	0.0909
	spinal meningioma	16.23	14	0.30	17.35	15	0.30	0.0944	0.0959	0.3418
	Spinal osteochondrosis	12.04	14	0.60	17.48	15	0.29	-0.1668	0.0715	0.0351
	Intracranial and intraspinal abscess	17.11	14	0.25	17.25	15	0.30	0.0299	0.0900	0.7448
	Cervical spinal cord and nerve injuries	10.76	14	0.70	10.81	15	0.77	-0.0126	0.0607	0.8391
	Glioblastoma	11.13	14	0.68	11.15	15	0.74	0.0135	0.1012	0.8960
	Benign meningioma	9.05	14	0.83	11.50	15	0.72	0.0453	0.0289	0.1396
	Malignant meningioma	10.37	14	0.74	10.50	15	0.79	0.0139	0.0385	0.7238
	Pituitary adenoma and craniopharyngioma	25.33	14	0.03	25.37	15	0.05	0.0070	0.0480	0.8853
	Benign neoplasm of brain and other parts of CNS	18.49	14	0.19	18.49	15	0.24	0.0024	0.0369	0.9489
	Malignant neoplasm of brain and other parts of CNS	8.27	14	0.88	9.32	15	0.86	0.0704	0.0687	0.3228
	Hydrocephalus	14.06	14	0.45	14.16	15	0.51	0.0110	0.0358	0.7644
	Craniosynostosis	11.61	14	0.64	11.93	15	0.68	-0.0275	0.0483	0.5775
	Concussion	13.49	14	0.49	13.49	15	0.56	-0.0004	0.0101	0.9671
	Diffuse brain injury	9.43	14	0.80	9.66	15	0.84	0.0185	0.0382	0.6357

	Focal brain injury	15.26	14	0.36	16.22	15	0.37	0.0293	0.0314	0.3662
	Congenital malformations of the nervous system	8.19	14	0.88	8.38	15	0.91	0.0265	0.0614	0.6724

Table S53 Sensitivity analyses of MR-Egger intercept regression and Cochrane Q tests.

Exposures	Outcomes	Q_MR.Egger	Q_df_MR.Egger	Q_pval	Q_IVW	Q_df_IVW	Q_pval	Egger_intercept	se	pval
PM2.5absorbance European	Trigeminal neuralgia	0.64	1	0.42	1.48	2	0.48	0.0948	0.1032	0.5268
	Epilepsy	5.97	2	0.05	9.43	3	0.02	-0.0618	0.0574	0.3941
	Parkinson's disease	0.95	2	0.62	1.55	3	0.67	-0.0376	0.0484	0.5186
	Alzheimer's disease	3.81	2	0.15	5.85	3	0.12	0.0210	0.0203	0.4100
	Major depressive disorder	0.32	1	0.57	0.37	2	0.83	-0.0248	0.1128	0.8622
	Obsessive Compulsive Disorder	4.19	2	0.12	5.29	3	0.15	0.0642	0.0889	0.5450
	Stroke	1.22	1	0.27	1.76	2	0.41	-0.0111	0.0167	0.6255
	Intracerebral hemorrhage	0.26	2	0.88	0.34	3	0.95	-0.0126	0.0471	0.8142
	Subarachnoid hemorrhage	2.69	2	0.26	3.40	3	0.33	0.0423	0.0584	0.5442
	Transient ischemic attack	0.05	1	0.82	0.52	2	0.77	-0.0223	0.0328	0.6194
	Cerebral infarction	0.22	2	0.89	3.89	3	0.27	0.0007	0.0004	0.1954
	Cerebral aneurysm	3.24	2	0.20	4.43	3	0.22	0.0543	0.0633	0.4813
	Cervical	0.18	2	0.91	0.1	3	0.98	-0.0004	0.00	0.91

	spondylosis				9				03	73
	Spinal canal stenosis	0.03	2	0.99	0.87	3	0.83	-0.0251	0.0273	0.4554
	spinal meningioma	0.59	1	0.44	0.79	2	0.67	0.1186	0.2664	0.7334
	Spinal osteochondrosis	0.01	1	0.91	1.13	2	0.57	0.2293	0.2170	0.4824
	Intracranial and intraspinal abscess	0.00	1	0.98	2.38	2	0.30	0.3805	0.2466	0.3662
	Cervical spinal cord and nerve injuries	2.61	1	0.11	3.72	2	0.16	-0.1912	0.2938	0.6326
	Glioblastoma	0.00	1	0.95	2.31	2	0.32	0.4536	0.2990	0.3710
	Benign meningioma	0.13	1	0.72	0.66	2	0.72	0.0629	0.0864	0.5995
	Malignant meningioma	0.25	1	0.62	2.68	2	0.26	0.1791	0.1149	0.3632
	Pituitary adenoma and craniopharyngioma	2.59	1	0.11	2.71	2	0.26	-0.0363	0.1725	0.8679
	Benign neoplasm of brain and other parts of CNS	1.12	1	0.29	1.13	2	0.57	0.0105	0.1013	0.9340
	Malignant neoplasm of brain and other parts of CNS	2.86	1	0.09	4.33	2	0.11	0.2490	0.3465	0.6033
	Hydrocephalus	0.19	1	0.67	0.20	2	0.91	0.0113	0.1062	0.9328
	Craniosynostosis	1.86	1	0.17	2.14	2	0.34	-0.0775	0.1978	0.7623
	Concussion	0.80	1	0.37	2.27	2	0.32	-0.0365	0.0301	0.4395
	Diffuse brain injury	1.08	1	0.30	6.84	2	0.03	0.2707	0.1173	0.2604

	Focal brain injury	0.21	1	0.64	3.29	2	0.19	0.1559	0.0889	0.3297
	Congenital malformations of the nervous system	1.63	1	0.20	1.67	2	0.43	-0.0357	0.2329	0.9032

Table S54 Sensitivity analyses of MR-Egger intercept regression and Cochrane Q tests.

Exposures	Outcomes	Q_MR.Egger	Q_df_MR.Egger	Q_pval	Q_IVW	Q_df_IVW	Q_pval	Egger_intercept	se	pval
PM2.5absorbance African American or Afro-Caribbean	Trigeminal neuralgia	19.51	20	0.49	19.71	21	0.54	-0.0169	0.0382	0.6629
	Epilepsy	20.00	21	0.52	20.28	22	0.57	-0.0071	0.0136	0.6078
	Parkinson's disease	23.86	21	0.30	24.48	22	0.32	0.0150	0.0202	0.4654
	Alzheimer's disease	15.77	20	0.73	16.64	21	0.73	0.0052	0.0056	0.3603
	Major depressive disorder	24.31	20	0.23	24.47	21	0.27	0.0072	0.0197	0.7167
	Obsessive Compulsive Disorder	26.53	19	0.12	26.89	20	0.14	-0.0142	0.0278	0.6148
	Stroke	27.06	20	0.13	27.17	21	0.17	0.0019	0.0067	0.7786
	Intracerebral hemorrhage	15.58	21	0.79	15.84	22	0.82	0.0097	0.0192	0.6172
	Subarachnoid hemorrhage	28.45	21	0.13	28.51	22	0.16	-0.0051	0.0238	0.8310
	Transient ischemic attack	15.14	20	0.77	15.91	21	0.77	-0.0108	0.0123	0.3890
	Cerebral infarction	14.22	21	0.86	14.33	22	0.89	-0.0004	0.0001	0.7431
	Cerebral aneurysm	18.95	21	0.59	19.34	22	0.62	-0.0121	0.0196	0.5430
	Cervical	27.15	21	0.17	27.	22	0.21	0.0000	0.00	0.94

	spondylosis				16				01	31
	Spinal canal stenosis	18.75	21	0.60	18.78	22	0.66	-0.0016	0.0106	0.8830
	spinal meningioma	17.36	20	0.63	18.88	21	0.59	-0.1220	0.0989	0.2314
	Spinal osteochondrosis	19.88	20	0.47	20.14	21	0.51	0.0408	0.0796	0.6135
	Intracranial and intraspinal abscess	16.37	20	0.69	16.46	21	0.74	0.0261	0.0907	0.7765
	Cervical spinal cord and nerve injuries	24.77	20	0.21	25.36	21	0.23	0.0516	0.0752	0.5007
	Glioblastoma	15.03	20	0.77	16.35	21	0.75	0.1296	0.1128	0.2640
	Benign meningioma	25.76	20	0.17	25.76	21	0.22	0.0015	0.0365	0.9677
	Malignant meningioma	18.37	20	0.56	18.56	21	0.61	-0.0189	0.0429	0.6657
	Pituitary adenoma and craniopharyngioma	11.61	20	0.93	12.42	21	0.93	0.0358	0.0399	0.3793
	Benign neoplasm of brain and other parts of CNS	23.66	20	0.26	24.59	21	0.27	0.0343	0.0388	0.3863
	Malignant neoplasm of brain and other parts of CNS	21.06	20	0.39	21.76	21	0.41	0.0637	0.0785	0.4264
	Hydrocephalus	19.55	20	0.49	20.28	21	0.50	0.0340	0.0397	0.4025
	Craniosynostosis	10.98	20	0.95	10.98	21	0.96	-0.0029	0.0535	0.9572
	Concussion	10.69	20	0.95	14.40	21	0.85	-0.0216	0.0112	0.0686
	Diffuse brain injury	18.85	20	0.53	22.86	21	0.35	-0.0847	0.0423	0.0590

	Focal brain injury	16.59	20	0.68	17.56	21	0.68	-0.0328	0.0334	0.3375
	Congenital malformations of the nervous system	37.37	20	0.01	41.73	21	0.01	0.1413	0.0924	0.1421

Table S55 Sensitivity analyses of MR-Egger intercept regression and Cochrane Q tests.

Exposures	Outcomes	Q_MR.Egger	Q_df_MR.Egger	Q_pval	Q_IVW	Q_df_IVW	Q_pval	Egger_intercept	se	pval
PM2.5absorbance South Asian	Trigeminal neuralgia	10.41	13	0.66	10.65	14	0.71	0.0225	0.0460	0.6337
	Epilepsy	11.39	13	0.58	13.10	14	0.52	0.0224	0.0172	0.2134
	Parkinson's disease	18.37	13	0.14	18.46	14	0.19	-0.0071	0.0284	0.8066
	Alzheimer's disease	10.26	13	0.67	10.41	14	0.73	-0.0027	0.0071	0.7052
	Major depressive disorder	4.83	10	0.90	6.27	11	0.85	-0.0365	0.0305	0.2584
	Obsessive Compulsive Disorder	8.83	13	0.79	10.04	14	0.76	0.0270	0.0246	0.2924
	Stroke	12.90	12	0.38	14.11	13	0.37	-0.0084	0.0079	0.3084
	Intracerebral hemorrhage	8.83	13	0.79	10.04	14	0.76	0.0270	0.0246	0.2924
	Subarachnoid hemorrhage	11.77	13	0.55	12.60	14	0.56	-0.0238	0.0263	0.3812
	Transient ischemic attack	10.62	13	0.64	15.75	14	0.33	0.0334	0.0148	0.0413
	Cerebral infarction	9.08	13	0.77	9.29	14	0.81	-0.0008	0.0002	0.6589
	Cerebral aneurysm	8.14	13	0.83	9.90	14	0.77	-0.0371	0.0280	0.2075
	Cervical	8.49	13	0.81	9.4	14	0.80	0.0001	0.00	0.34

	spondylosis				6				01	30
	Spinal canal stenosis	13.85	13	0.38	14.21	14	0.43	-0.0080	0.0137	0.5702
	spinal meningioma	8.28	13	0.82	9.33	14	0.81	0.1221	0.1192	0.3244
	Spinal osteochondrosis	18.63	13	0.14	19.84	14	0.14	0.1056	0.1149	0.3752
	Intracranial and intraspinal abscess	10.06	13	0.69	10.08	14	0.76	-0.0143	0.1090	0.8977
	Cervical spinal cord and nerve injuries	14.65	13	0.33	15.35	14	0.35	0.0682	0.0865	0.4446
	Glioblastoma	10.92	13	0.62	12.26	14	0.59	0.1575	0.1356	0.2663
	Benign meningioma	13.87	13	0.38	14.27	14	0.43	0.0244	0.0399	0.5524
	Malignant meningioma	13.98	13	0.38	18.26	14	0.20	0.1066	0.0534	0.0673
	Pituitary adenoma and craniopharyngioma	10.90	13	0.62	10.90	14	0.69	-0.0013	0.0478	0.9791
	Benign neoplasm of brain and other parts of CNS	10.07	13	0.69	12.34	14	0.58	-0.0645	0.0429	0.1561
	Malignant neoplasm of brain and other parts of CNS	11.39	13	0.58	11.48	14	0.65	0.0272	0.0921	0.7720
	Hydrocephalus	4.14	13	0.99	5.25	14	0.98	0.0501	0.0478	0.3130
	Craniosynostosis	12.08	13	0.52	12.08	14	0.60	0.0006	0.0643	0.9922
	Concussion	9.05	13	0.77	11.27	14	0.66	-0.0201	0.0135	0.1605
	Diffuse brain injury	8.61	13	0.80	9.38	14	0.81	0.0448	0.0510	0.3957

	Focal brain injury	13.35	13	0.42	14.36	14	0.42	0.0405	0.0408	0.3388
	Congenital malformations of the nervous system	11.76	13	0.55	13.14	14	0.52	0.0957	0.0814	0.2609

Table S56 Sensitivity analyses of MR-Egger intercept regression and Cochrane Q tests.

Exposures	Outcomes	Q_MR.Egger	Q_df_MR.Egger	Q_pval	Q_IVW	Q_df_IVW	Q_pval	Egger_intercept	se	pval
PM2.5absorbance East Asian	Trigeminal neuralgia	5.44	9	0.79	5.64	10	0.84	0.0277	0.0612	0.6610
	Epilepsy	16.04	9	0.07	16.13	10	0.10	0.0067	0.0288	0.8213
	Parkinson's disease	4.01	9	0.91	4.03	10	0.95	-0.0046	0.0290	0.8769
	Alzheimer's disease	7.06	9	0.63	7.52	10	0.68	-0.0056	0.0082	0.5161
	Major depressive disorder	9.52	9	0.39	10.13	10	0.43	0.0247	0.0325	0.4674
	Obsessive Compulsive Disorder	12.48	9	0.19	13.59	10	0.19	-0.0372	0.0416	0.3944
	Stroke	13.85	9	0.13	14.60	10	0.15	-0.0077	0.0110	0.5025
	Intracerebral hemorrhage	3.83	9	0.92	3.92	10	0.95	0.0090	0.2990	0.7703
	Subarachnoid hemorrhage	7.93	9	0.54	8.00	10	0.63	-0.0081	0.0317	0.8044
	Transient ischemic attack	6.65	9	0.67	6.73	10	0.75	-0.0056	0.0197	0.7824
	Cerebral infarction	7.22	9	0.61	7.47	10	0.68	-0.0001	0.0002	0.6244
	Cerebral aneurysm	10.53	9	0.31	10.71	10	0.38	-0.0140	0.0358	0.7060
	Cervical	3.51	9	0.94	3.9	10	0.95	-0.0001	0.00	0.53

	spondylosis				2				02	59
	Spinal canal stenosis	11.14	9	0.27	11.96	10	0.29	-0.0146	0.0180	0.4383
	spinal meningioma	12.01	9	0.21	12.92	10	0.23	0.1502	0.1822	0.4310
	Spinal osteochondrosis	5.73	9	0.77	7.78	10	0.65	-0.1826	0.1274	0.1855
	Intracranial and intraspinal abscess	8.42	9	0.49	9.90	10	0.45	0.1760	0.1448	0.2550
	Cervical spinal cord and nerve injuries	5.72	9	0.77	7.90	10	0.64	0.1595	0.1080	0.1739
	Glioblastoma	9.84	9	0.36	10.03	10	0.44	0.0783	0.1880	0.6870
	Benign meningioma	11.26	9	0.26	11.57	10	0.31	-0.0286	0.0574	0.6307
	Malignant meningioma	4.62	9	0.87	5.98	10	0.82	-0.0796	0.0685	0.2750
	Pituitary adenoma and craniopharyngioma	2.35	9	0.98	2.37	10	0.99	-0.0106	0.0637	0.8720
	Benign neoplasm of brain and other parts of CNS	15.00	9	0.09	15.01	10	0.13	-0.0056	0.0735	0.9412
	Malignant neoplasm of brain and other parts of CNS	5.55	9	0.78	6.95	10	0.73	0.1446	0.1222	0.2671
	Hydrocephalus	6.23	9	0.72	6.49	10	0.77	0.0325	0.0637	0.6219
	Craniosynostosis	4.13	9	0.90	4.21	10	0.94	-0.0246	0.0857	0.7805
	Concussion	10.97	9	0.28	11.85	10	0.30	0.0170	0.0200	0.4173
	Diffuse brain injury	4.64	9	0.86	8.07	10	0.62	-0.1258	0.0679	0.0971

	Focal brain injury	11.74	9	0.23	14.13	10	0.17	0.0830	0.0613	0.2087
	Congenital malformations of the nervous system	15.92	9	0.07	17.10	10	0.07	-0.1177	0.1444	0.4360
